# Federation of European Laboratory Animal Science Associations recommendations of best practices for the health management of ruminants and pigs used for scientific and educational purposes

**DOI:** 10.1177/0023677220944461

**Published:** 2020-08-09

**Authors:** FELASA Working Group on Farm Animals: Corina Mihaela Berset (Convenor), Maria Emiliana Caristo, Fabienne Ferrara, Patrick Hardy, Marianne Oropeza-Moe, Ryan Waters

**Affiliations:** 1 Animal Welfare and 3R Department, University of Zurich, Switzerland; 2 Experimental Research Center, Catholic University of Sacred Heart, Italy; 3 Consulting and Training in Laboratory Animal Science, Germany; 4 Veterinary and Professional Services, Allentown France, France; 5 Norwegian University of Life Sciences (NMBU), Norway; 6 The Pirbright Institute, UK

**Keywords:** FELASA, ruminants, pigs, organisms and models, recommendations, health

## Abstract

Most ruminants and pigs used for scientific and educational aims are bred not for these purposes but in a farm environment. Given the wide range of diseases that these species might have, ensuring that the animals’ health status is appropriate can be complex and challenging. The Federation of European Laboratory Animal Science Associations has previously published recommendations for the health monitoring of experimental colonies of pigs (1998) and, respectively, calves, sheep and goats (2000). Unfortunately, the uptake of those recommendations was poor and insufficiently reported in scientific publications. These new recommendations for best practice focus on the main species of ruminants (cattle, sheep and goats) and pigs. They provide general and specific information helpful for designing a health management programme for the suppliers and for the user establishments, as well as guidance on animal procurement. Critical thinking based on the fields of use of the animals is promoted, aiming to help in taking informed decisions rather than establishing an exhaustive exclusion list for pathogens. Implementing the best health and welfare management practices should be done under the guidance of a competent attending veterinarian, with expertise and sufficient authority to take the appropriate action, doubled by excellent communication skills. It is strongly recommended that the user establishment’s veterinarian works in close collaboration with the supplier’s veterinarian.

## Résumé

La plupart des ruminants et des porcs utilisés à des fins scientifiques et éducatives ne sont pas élevés à ces fins, mais dans un environnement agricole. Compte tenu de la vaste gamme de maladies que ces espèces pourraient contracter, il peut être complexe et difficile de s’assurer que l’état de santé des animaux soit approprié. La FELASA a déjà publié des recommandations de surveillance sanitaire des colonies expérimentales de porcs (1998) et, respectivement, de veaux, de moutons et de chèvres (2000). Malheureusement, l’application de ces recommandations était médiocre et insuffisamment rapportée dans les publications scientifiques. Ces nouvelles recommandations de meilleures pratiques portent sur les principales espèces de ruminants (bovins, ovins et caprins) et de porcs. Elles fournissent des informations générales et spécifiques utiles à la conception d’un programme de gestion de la santé pour les fournisseurs, les établissements utilisateurs, ainsi que des conseils sur les achats d’animaux. La pensée critique basée sur les domaines d’utilisation des animaux est encouragée, visant à aider à prendre des décisions éclairées, plutôt qu’à établir une liste exhaustive d’exclusion des agents pathogènes. La mise en œuvre des meilleures pratiques de gestion de la santé et du bien-être devrait avoir lieu sous la direction d’un vétérinaire traitant compétent, ayant l’expertise et l’autorité suffisante pour prendre les mesures appropriées, ainsi que d’excellentes compétences en communication. Il est fortement recommandé que le vétérinaire de l’établissement utilisateur travaille en étroite collaboration avec le vétérinaire du fournisseur.

## Abstract (de)

Die meisten Wiederkäuer und Schweine, die zu wissenschaftlichen und Ausbildungszwecken dienen, werden nicht eigens dafür, sondern in einem landwirtschaftlichen Umfeld gezüchtet. Angesichts des breiten Spektrums potenzieller Krankheiten bei diesen Tierarten kann die Gewährleistung eines adäquaten Gesundheitszustands der Tiere durchaus komplex und schwierig sein. FELASA hat bereits früher Empfehlungen für die Gesundheitsüberwachung von Versuchskolonien von Schweinen (1998) bzw. Kälbern, Schafen und Ziegen (2000) veröffentlicht. Leider stießen diese Empfehlungen auf mangelndes Echo und wissenschaftliche Publikationen berichteten diesbezüglich nur unzureichend. Die vorliegenden neuen Empfehlungen für beste Praxis konzentrieren sich auf die wichtigsten Arten von Wiederkäuern (Rinder, Schafe und Ziegen) sowie auf Schweine. Sie enthalten allgemeine und spezifische Informationen, die für die Gestaltung eines Gesundheitsmanagementprogramms für die Lieferanten und für die Verwendereinrichtungen nützlich sind, ebenso wie Hinweise zur Tierbeschaffung. Kritisches Denken auf der Grundlage der Anwendungsbereiche der Tiere, das darauf abzielt, fundierte Entscheidungen zu treffen, anstatt eine erschöpfende Ausschlussliste für Krankheitserreger zu erstellen, wird unterstützt. Die Umsetzung der besten Gesundheits- und Tierschutzmanagementpraktiken sollte unter der Anleitung eines kompetenten behandelnden Tierarztes erfolgen, der über Fachwissen und genügend Autorität sowie über ausgezeichnete Kommunikationsfähigkeiten verfügen, um die entsprechenden Maßnahmen zu ergreifen und zu vermitteln. Eine enge Zusammenarbeit zwischen dem Tierarzt der Verwendereinrichtung und dem Tierarzt des Lieferanten wird dringend empfohlen.

## Resumen

La mayoría de rumiantes y cerdos utilizados para objetivos científicos y educativos no son criados para estos fines, sino que se crían en una granja. Dado el amplio abanico de enfermedades que estas especies pueden tener, garantizar que la salud de estos animales sea adecuada puede ser algo complejo y complicado. FELASA anteriormente ha publicados recomendaciones para la gestión de la salud de colonias de cerdos para fines experimentales (1998) y, respectivamente, terneros, ovejas y cabras (2000). Lamentablemente, la implementación de estas recomendaciones no fue muy buena y dichas recomendaciones no aparecieron en muchas publicaciones científicas. Estas nuevas recomendaciones de mejores prácticas se centran en las principales especies de rumiantes (bovinos, ovejas y cabras) y cerdos. Estos ofrecen información general y específica de gran utilidad para diseñar un buen programa de gestión de la salud para los proveedores y para los establecimientos de los usuarios y sirven de guía para la adquisición de animales. Se fomenta un pensamiento crítico basado en los campos de uso de los animales, que trata de ayudar a tomar decisiones documentadas, en lugar de establecer una lista de exclusión exhaustiva para patógenos. Implementar las mejores prácticas sobre gestión de bienestar y salud debería hacerse siguiendo la guía de un veterinario competente y responsable, con experiencia y suficiente autoridad para llevar a cabo las acciones necesarias, junto con gran experiencia en comunicación. Se recomienda encarecidamente que el veterinario del establecimiento del usuario trabaje en estrecha colaboración con el veterinario del proveedor.

## 1. Introduction

Ruminants and pigs are frequently used in a wide array of scientific research areas, including agricultural and veterinary research where they represent the target species. Other areas such as biomedical research employ ruminants and pigs as model species for basic or translational studies or for other scientific purposes. The use of agricultural animals for educational purposes is done in all areas.^1–7^

In contrast to small animal species, ruminants and pigs (except for miniature pigs) are often not bred purposefully for scientific and educational aims. They instead exist as part of the food production system and are acquired from farm premises. They are also obtained from specific breeders outside the livestock business. Indeed, in the EU, there is no legal requirement to acquire ruminants and pigs from suppliers which produce them specifically for scientific and educational purposes (unlike the species listed in Annex I to Directive 2010/63).^8^

A central component of all previous Federation of European Laboratory Animal Science Associations (FELASA) recommendations on health monitoring in breeding and experimental units has been the ‘pathogen list’ – a list of microbiological agents which were first interpreted as an exclusion list.^9^ Revisions to the rodent FELASA recommendations^10^ have clarified that most agents to be monitored should vary depending on what effect they have on animal health and on the specific studies being undertaken.

The overall actual practical implementation of the previous FELASA recommendations for monitoring the health of experimental units of calves, sheep and goats,^11^ and pigs,^12^ remains largely unknown, as it was not reported in scientific publications. The only information about the relative poor uptake of these recommendations was obtained via two recent European surveys.^13,14^ This apparently lower adherence is likely due to the practically impossible screening of all agents which were listed in those recommendations in a farm environment.

The main goal of these new FELASA recommendations is to provide practical guidance for an optimal health management programme for ruminants and pigs used for scientific and educational purposes. This is a very complex and challenging task, considering the wide range of facilities from which these animals are sourced and the heterogeneity of suppliers and of the user establishments. Furthermore, farm animals may also be carrying zoonotic organisms, which is relevant to occupational health in user establishments.^15^

By stimulating critical thinking based on the fields of use of the animals, these new recommendations aim to help in taking professional and informed decisions rather than establishing an exhaustive exclusion list of all known pathogens. Defining a health management and monitoring programme should be a prerequisite to any planning of future studies.^16^ Subsequently, adequate reporting of this in scientific publications should be common practice in order to help improve the reproducibility of in vivo studies.^17^

A successful, comprehensive and relevant health management and monitoring programme relies on expert professional judgement and cannot be based on ‘recipes’. In consequence, a prerequisite of paramount importance applicable to a successful and relevant health management programme is to be designed by a competent and skilled attending veterinarian and conducted under her/his supervision. The attending veterinarian should also have sufficient authority to take the appropriate action, accompanied by excellent communication skills.

Noteworthy, these recommendations do not address all farm animal species; they only focus on the main species of ruminants (cattle, sheep and goats) and pigs. Additional information (details and examples) is provided as Supplemental Material. This document is addressed to suppliers and destination facilities for ruminants and pigs used for scientific and educational purposes. These recommendations are not meant only for EU member states. In all cases, the national and international relevant legislation should prevail.

For the purpose of this report, the term ‘specific pathogen-free (SPF)’ will not be used, as it is deemed too imprecise. The terms ‘high confidence/stringency in health status’ will be used instead.

## 2. General information on the health management of ruminants and pigs

For practical and economic reasons, when a comprehensive health definition is required (i.e. more demanding than a simple demonstration of good clinical condition, free from any clinical evidence of disease), the vast majority of animals used in research are not ‘gnotoxenic’ (with a ‘positive’ and exhaustive definition of their microbiota, including any infectious, opportunistic or commensal agent) but rather are defined as ‘agnotoxenic’ (with no positive and comprehensive definition of their microbiota) and defined according to an ‘exclusion list’ of undesirable agents (high stringency in health status).

It is important to appreciate the difference between the *screening* list and the *exclusion* list. The former can be purely informative and may include the monitoring of resident microbiota as an indication of the efficiency of the bio-exclusion precautions. The exclusion list is of more immediate scientific importance because if a positive result is found, this may invalidate experimental results and perhaps may lead to a major eradication programme, or even replacing the colony with all related ethical, practical and economic consequences.

The key factors to be considered for the health management of animals are:

1. An appropriate ‘health standard’ or ‘microbiological status’ of the animal. This implies the absence of specific pathogenic microorganisms and the presence of an associated microbiota which is fully consistent with the desired characteristics of the animal model. This includes its specific and non-specific immunological competence in relation to the research application in order to guarantee the absence of interfering factors. Biosecurity safeguards and a health monitoring programme tailored to verify the specified health standard of animals concerned must be put in place.
2. Existence of a policy on testing (entire herd/representative sample size/animals shortlisted for being supplied, depending on the customer’s needs). The nature, the sample size and the frequency of health monitoring of a herd, colony or group of animals should be tailored locally and according to many considerations, such as the source of the animals, the housing conditions, the health management programme (with physical and procedural barriers), the history and the assessed risk of contamination on site, the intended research use of the animals and the related exclusion list.
3. Clear communication and good collaboration with the various suppliers (of animals, consumables, equipment, services, etc.) or research partners. It is highly recommended to make sure that the animal supplier complies not only with the regulations in force but also with key requirements such as quality management and organisation, adequacy of resources (quantitative and qualitative, in various expertise fields) and the implementation of adequate animal welfare standards in terms of procedures and practices. This can be achieved by visiting/auditing the facilities and/or by relying on accreditation schemes (such as AAALAC International – which can be instrumental in setting up a customer audit). As housing conditions and care in the breeding facility may influence the animal model and the study outcome, their understanding is also very useful to the investigator. In some cases, it may be critical to develop a real partnership with the supplier, including discussing and setting particular specifications.
4. Adequately trained staff with ample experience with the relevant species.

### 2.1. Quality and technical agreement

It is strongly recommended to issue and sign a quality and technical agreement with the breeder. A quality and technical agreement is a contractual document defining the ‘quality’ requirements (quality system) and other ‘technical’ aspects (specifications related to the animal) of the agreement between the user establishment and the breeding institution/supplier with regard to the breeding and care, testing and quality assurance operations of animal breeding or any other critical supply or service. It is established according to the user establishment’s needs, including animal welfare and 3Rs (reduce-refine-replace) requirements and to the commitments and obligations of both parties. It includes all key items such as responsibilities, resources, communication, documentation, change control, deviation and complaint management, audit and so on.

Generally, the core of the document includes a general quality policy requirement between the two parties, and one or several appendix/appendices cover the ‘technical specifications’, each addressing one category of service (e.g. one type of supply) contracted and details all the technical information necessary for its proper execution (e.g. the detailed health definition of animals in a breeding colony, the husbandry, genetic management and monitoring programmes, the shipment conditions etc.).

## 3. Health management considerations for animal procurement

### 3.1. Animal procurement from dedicated breeding colonies

#### 3.1.1. General principles

**Figure 1.**
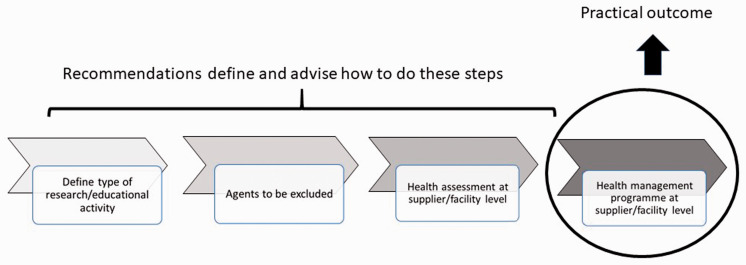
Steps for the establishment of a health management programme for farm animals used for research or educational purposes.

Each specialised breeder or user should establish, validate and assure a more or less restrictive definition of ‘high confidence/stringency in health status’ which matches the specific expectations and scientific/educational activities for which the animals are to be used and which clearly references the methods used to control exclusion and to monitor the microbiological status of the animals (Figure 1). On the exclusion list, the agents subject to national and international animal health programmes should be marked as such because there will be information on the animals health status available through other means. One should pay attention to the risk related to *healthy carriers*, which could host (and intermittently shed) pathogenic, parasitic or zoonotic agents without displaying any clinical signs or lesion. A relevant health monitoring programme based on serology and/or direct demonstration of the agent is the best method in the attempt to guarantee the absence of carriers.

The specialised breeder or supplier is expected to provide: (a) the list of agents for which screening is carried out, the frequency of testing and sampling strategy and methods; and (b) the exclusion list, with a predefined policy for each agent which may be identified. For example, policies may include immediate termination or recycle of the colony if the contaminating agent is pathogenic or may interfere substantially with scientific use; delayed or planned recycle of the colony for agents which interfere only in a minor way, for example if there are very few experimental projects or research activities which would be affected; or no action if the microorganisms are generally harmless opportunistic agents.

Some studies may require the use of animals free of specific antibodies. This is usually the case for research and development of veterinary vaccines and research of infectious diseases. In this case, serological screening is not only a means of monitoring the absence of the microorganisms in the breeding colony, but also a prerequisite for some categories of vaccine efficacy and safety studies.

#### 3.1.2. Health management and monitoring sampling procedure

Different colonies – even from the same commercial vendor – may have been raised under differing husbandry and environmental influences, resulting in differing incidences of infections. Each institution should establish an agricultural animal care and use programme with clearly designated lines of authority in accordance with the applicable governmental laws, regulations and institutional policies. Differences in environmental and microbial conditions between commercial breeders and between production facilities within a commercial breeding operation can be substantial and may affect study outcomes depending on the types of end points studied. Therefore, they should be reported in scientific publications.^17^

#### 3.1.3. Bio-exclusion, bio-containment and health monitoring

Maintenance of a defined health standard requires a properly implemented and comprehensive ‘bio-exclusion’ programme, aiming at ‘zero contamination’ of the animal colonies. It requires careful facility design, including suitable finishes of floors, walls, ceilings, housing, caging and handling equipment, and rigorous application of procedures associated with education and training of personnel. A health-monitoring programme should be designed, kept under review and duly implemented. Microbiologically undefined or contaminated animals should be kept under adequate ‘bio-containment’ or quarantine conditions by applying appropriate safety measures in order to prevent the spreading of undesirable or hazardous infective agents to cleaner animals or to humans.

#### 3.1.4. Colony termination and recycling policy

There are occasions when it is necessary to terminate, or to re-derive a colony. Examples are: (1) as quickly as possible when a major colonisation with infectious agent(s) not compatible with the animal health and welfare or with research-related key requirements, as defined when the exclusion list was established at colony foundation. The most appropriate technique depends on the number and the nature of the agent(s) to be eliminated and on the expected use of the animals. In cases of agents transmitted from animal to animal, removing only the animals that were tested positive is not considered effective elimination of the agent from the colony (although it may reduce the infection pressure and the prevalence substantially). If the agents are transmitted via vectors (such as insects), the elimination of the animals tested positive might be sufficient (only if the vectors are also eliminated). (2) Less urgently if curative/preventive measures are available to eliminate the agents, in case of minor colonisation(s) with infectious agent(s) is/are not associated with overt pathology and with key research requirements. The possible outcomes of the treatment should be part of the evaluation.

### 3.2. Animal procurement from agricultural premises (farm suppliers)

Any disease process – whether nutritional, environmental, neoplastic or infectious – needs to be controlled for. Given the relatively uncontrolled environment under which they are produced and the intensive nature of their productivity, it is much more difficult to control these factors than with rodent models.

A thorough review of on-farm management, any site-associated diseases and thorough periodic clinical examination are necessary to ensure the provision of healthy animals. Moreover, the typical lifespan of agriculture animals is often limited in connection to their normal use. If the animals need to be kept much longer for research purposes, unusual clinical observations or lesions may be observed.

#### 3.2.1. Criteria for selection of farm suppliers

Agricultural settings may differ very significantly according to their main activity (genetic selection, breeding, growing), their location, outdoor or indoor housing and so on. Farm visits should also be undertaken. The first visit is very important in order to examine how the animals are housed and the biosecurity measures in place and to ask questions which may be relevant to specific uses of the animals. If the user establishment then decides to collaborate with the farm, periodic visits should be carried out, and a collaboration agreement should be prepared and put in place. When relevant, the collaboration agreement may include a financial and technical contribution of the user establishment, allowing improvements of animal housing conditions and working practices. In many countries, livestock farms can participate in quality programmes aimed at food safety and animal welfare. These programmes can also be used for an initial selection of suppliers.

**Figure 2.**
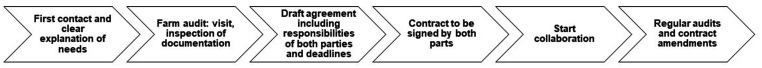
Steps for selecting a farm supplier for ruminants and pigs used for biomedical research.

The main criteria to be considered for evaluation of a potential supplier are:

- the type of facility and animal housing conditions, and the probability of whether the supplier maintains a health management programme (protection against wild animals and pest, suitable animal flows on site, possibility to conduct adequate cleaning and disinfection practices etc.);
- the health status history of the farm and number and type of checks that are performed (herd health monitoring plan, including information on clinical examination,^5,18–21^ body condition scoring (BCS)^18,22^);
- verifying if and how often the breeder introduces new animals (coming from other suppliers) or biological material and checking the health reports accordingly;
- the proximity of the farm to the research centre where animals will be used;
- the availability of facilities that allow the separation of animals in case of need (quarantine, treatments etc.);
- checking the means by which the animals are transported and the duration between loading and delivery;
- clear and transparent communication and mutual trust are essential for a good collaboration (awareness that notifiable diseases found unexpectedly must be reported to the competent authorities and, as a component of the quality and technical agreement, that any significant event impacting the animals and colony management should be reported to the research institution);
- formalising the response to specifications in a collaborative agreement, signed by both parties, which will help defining reciprocal obligations, clarifying future communication and helping conducting periodic audits (Figure 2); and
- the capacity of the farm – the ability to deliver the number of qualified animals requested at the time requested.

It is essential that every potential supplier has its health management system reviewed by a veterinarian from the user establishment (named veterinary surgeon, designated veterinarian or equivalent) whose objective is to assess the current health and welfare status, preferably together with the farm veterinary practitioner. The common documentation which should be reviewed would include: (a) a herd health plan, (b) a farm biosecurity policy, (c) any specific/official health certificates the farm may hold and related records and (d) records related to the use of veterinary pharmaceuticals.

#### 3.2.2. Criteria for animal selection

Once any herd/flock-wide health-monitoring programmes have been defined/implemented, individual assessments of the animals which have been identified for inclusion in the study (preselected) can be explored. Of primary importance is that the animals being selected are not those which are considered of least market value to the farm. These animals should be free of any clinical disease and of good general condition. A thorough clinical examination (performed by a veterinarian) and BCS are easy to perform and provide very valuable information and should be conducted in the first phase of the animal selection process. Any weight data/feed intake data which are available could also be requested. A variety of individual health screening tests could be applied to the animals to help give further assurance that there are no ongoing subclinical health problems or infections which may affect the downstream research. When considering these tests, applicable national legal constraints (e.g. when invasive sampling is considered a regulated procedure) should be taken into account. The types of screening can be classified into two categories: (a) general health screening and (b) microorganism-specific screening.

##### 3.2.2.1. General health screening

A complete clinical examination, checking for clinical abnormalities and predisposing disease risk factors, can lead to a problem-orientated method based on the complete examination and differential diagnoses generated by the findings. A complete clinical examination will include signalment of the patient, history of the patient(s), the farm history, observation of the environment, the animals at a distance, detailed observations of the animals and examination of selected animals.^23^ The animals should be examined from the snout to the tail, using inspection, palpation and auscultation.

The clinical examinations should be performed in a stringently uniform manner and documented on an individual score sheet:

- Body posture
- Inspection and palpation of the head, including the mandible, teeth and oral cavity, neck, left thorax and abdomen, right thorax and abdomen, tail end, vaginal examination, rectal examination, udder or, in male animals, external genitalia
- Consistency of the faeces (formed or smooth, colour, odour, anal region and dirty tail)
- Auscultation of the lungs, heart and stomach/intestines
- Heart and respiration rate
- Respiration type (abdominal/costal)
- Symmetry of locomotion
- BCS

BCS is an important tool for flock management. BCS of pigs is based on palpation of the ribs, hips, and backbone, and ranges from a BCS of 1 (excessively thin) to a BCS of 5 (excessively fat).^20^ For cattle, sheep and goats, the scoring systems most frequently used for BCS are numerical rating scales, with five-,^24,25^ six-^26–29^ or eight-point^30^ scales. These scoring systems are usually divided into intermediate scores (0.25 or 0.5) that result in 13- to 21-point scoring systems.^31^

In addition to organism specific testing, it may be useful to assess the physiological health of the animals by examining their haematological/biochemical status, for instance by analysing blood samples taken for specific disease investigation. A haematological and biochemical profile may not only pick up evidence of an undetected ongoing infectious process, it may also detect other physiological derangements. The relevant literature should be consulted.^32–34^

##### 3.2.2.2. Agent-specific screening

Viruses, bacteria, fungi, endo- and ectoparasites will all be present in an on-farm environment, and it will be impossible to stop incursions of these organisms, given the open nature of agricultural practice. Consideration must be given to which infectious organisms the researcher requires animals to be free of. As stated, any animals which have a history of clinical disease signs will not be utilised for use on a study, and thus all individual screening will be to give assurance of any underlying infection/disease not clinically apparent. It must be noted from a fundamental perspective that all infections, whether clinical or subclinical, will lead to biological variability. Also, the normal microbiome and its variations could influence model performance. Hence, the farm should inform about aspects of animal management and changes that may influence this (e.g. diet and medication). However, it is essential that any active diseases present within a herd/flock are thoroughly investigated – an arrangement around which the researcher and farm/farm veterinarian can be made with respect to subsidising any diagnostic procedures such as laboratory tests. The actual pathogens being tested for will be either based on its likely potential to damage animal health or be an uncontrolled and significant confounding factor in experimental studies and/or its zoonotic potential.

Screening methods and laboratory diagnostic tests are very dynamic, being continuously developed and improved. Therefore, no specific recommendation is made with respect to which method can be used because the methods might become obsolete very fast. In regard to the periodicity of screening and the number of animals sampled, very different strategies may be used, depending on the context and the objective. In some cases, an entire group of animals may have to be individually and repeatedly sampled, for example before introduction in an established colony benefiting from defined and suitable health definition. In other cases, the sampling may be conducted periodically (at a predefined frequency) and limited to a representative sample, on a limited and defined number of individuals, for example for a routine/periodic health checks in stable closed colony, with no suspicion of infection or contamination. Conducting a risk assessment is of paramount importance for designing the health management programme.

It is very important to have appropriate written documentation, including the detailed description of the health management programme, health certificates listing the agents for which the animals were tested/screened, the number of animals tested and the number of positive results, historical results and measures taken during the past 18 months to address positive findings, the laboratory method used for testing/screening, the name of the laboratory where the tests have been performed. A good template for the health certificate can be found in other FELASA Working Group Reports.^10^

##### 3.2.2.3. Gestation status check

For studies with pregnant animals, appropriate details should be given regarding the source of the animals (procured from external breeding sources or bred internally). Furthermore, exposure to adverse effects resulting from the experiment during the last third of gestation would imply that also the foetuses are subjected to regulated procedures, so defined in Article 1 paragraph 3 (a)(ii) of Directive 2010/63/EU. In the EU, the transport of animals near the expected parturition date is forbidden. Breeding conditions and gestational age at shipment before experimental use are important information to provide, as well as details about litter sizes. The transport of very early pregnant females, although not being forbidden, should be avoided. Implantation should be safe to prevent early resorption of valuable foetuses. Experimental results can be strongly influenced by breeding, especially for end points and physiological states that are dependent on endocrine factors.^35^ Good communication between supplier and user are essential. Gestation/barren state can be diagnosed using various methods, depending on the gestational age, including blood tests for the measurement of progesterone (in sows), and of the pregnancy-associated glycoprotein (in ruminants), rectal palpation (in sows and cows) and ultrasound examination (in sows and ruminants).

## 4. Transport from supplier to user establishment

The transport of pigs and ruminants should be organised and conducted in a manner that is compliant with the relevant legislation and takes into account animal welfare, physical safety considerations and biosecurity.^36^ If the hygienic and health status are the same, it would be preferable to choose a supplier as close as possible to the user establishment in order to reduce the stress of the transported animals. If purchasing animals includes importation of animals, the current legislation of the exporting, transit and importing countries must be followed. Public holidays of all these countries should be considered to avoid unforeseeable interruptions of the transport process.

## 5. User establishment

The same health management strategies that apply for the suppliers also apply to the user establishments. Any effort should be undertaken to continue or increase the standard when the animals have arrived in the user facility, as it will be described further in this article.

Housing farm animals in experimental facilities is a great challenge, as the following aspects have to be considered: (a) separation of animals from different origin farms and securing of adequate quarantine procedures; (b) separation of different species, sexes and ages; and (c) meeting legal requirements for keeping ruminants and pigs. When using agriculture animals for research purposes, the applicable regulation and standard are Directive 2010/63/EU^8^ and Convention ETS 123, even if animals were originating from agriculture settings implementing regulations applicable to farm animals. Agriculture standards could be applicable for scientific reasons if it can be demonstrated that the purposes of the study require it to be conducted in farming conditions. Finally, in addition to these European regulations and standards applicable to the welfare of farms animals, the FASS Guide for the Care and Use of Agricultural Animals in Research and Teaching^37^ provides interesting recommendations. Accommodation of farm animals should allow the execution of their ‘natural behavior, in particular the need to graze or forage, exercise and socialize’. Group housing of compatible animals is particularly important, as they are social animals with a strong motivation to interact with conspecifics.

Foot-care management, parasite control measures (considering potential resistance to anti-parasitic treatments) and regular review of production indices and BCS^18,22,24–28,30,31^ are very important. The detailed description of these is beyond the scope of this report – relevant, recent publications should be consulted. All procedures and findings should be documented, and a facility database should be created and kept up to date for further reference and appropriate reporting in scientific publications.

Documented and authorised standard operating procedures are highly recommended, as they represent valuable resources for reference and training of (new) employees. Regarding the experimental housing of domestic pigs and ruminants as a model for human diseases, a basic standard monitoring programme and barrier system must be developed for each research institution. In accordance with the specific research field and study settings, it is important to set up a more individual approach to health and welfare assessment and management. Appropriate measures to ensure the animals’ well-being (good physical and mental health) should be considered (environmental enrichment, where positive interactions with other animals and staff play a very important role).

Barrier systems must be implemented based on a risk assessment of cross-infection and could range from only changes of clothes, wearing face masks and/or shoe covers or special air filter systems, such as gravimetric or high-efficiency particulate air (HEPA) filters, combined with air pressure differentials as needed. Data from a query in Germany, Switzerland and Austria^14^ clearly show that in most research institutions, the barrier systems only include changes of clothes and wearing face masks and shoe covers.

The hygiene and husbandry procedures should be based on a risk assessment and should be proportional to the type of experiment being performed and its biosafety level. All surfaces should allow appropriate decontamination and at the same time be safe for the animals and the facility’s personnel. Furthermore, cross-contamination by waste handling should be prevented.

### 5.1. Acclimatisation and quarantine

#### 5.1.1. Acclimatisation

The shipping process is stressful for animals and disrupts their normal environment. The purpose of acclimatisation is to allow the animals to recover from the stress of transport and to adjust to the new environment (housing and caging conditions, social group, watering system and food, staff, etc.). During this period, all efforts should be made to minimise the impact of the new environment by initially retaining the same social groups, litter material or food and only gradually introducing the materials which will be used during the study. The time for acclimatisation (from the moment the animal arrives at the research center until it is used) depends on various factors, such as the age of the animal, the type and duration of transportation, geographic considerations (e.g. climate, altitude),^38,39^ the type of research (acute or chronic projects) and the age the animals need to be for the study (e.g. post-weaning piglets). Another very important factor that determines the acclimatisation time is the relationship of trust and confidence that the animals must acquire with the people who will take care of them at all levels. The acclimatisation process should be completed prior to the experimental use of the animals.

#### 5.1.2. Quarantine

Quarantine is a procedure that requires the isolation of groups of animals awaiting outcomes of health assessment, in particular to protect the health of animals already in the facility. Where a facility takes only one batch of animals (of the same age from the same supplier) at a time and applies all-in/all-out with sanitation in between, the animals should be housed in the same holding room during quarantine and experimental procedures. Similarly, if there are multiple holding rooms that can be properly isolated, these could serve as holding rooms during quarantine and subsequently during experimental use, without moving the animals again and thus mitigating stress. The quarantine requirements can be met by a variety of combinations of physical provisions and procedures. Quarantine allows subclinical disease to manifest after exposure to the new environment at the user establishment before the animals are used for studies where these confounding phenomena could jeopardise the validity of the research. During the quarantine period, the health of the animals is monitored clinically (individual clinical score sheet), and a screen (diagnostic tests) is performed before a decision is made about whether to introduce them to the experimental setting. The quarantine duration depends on the incubation period of the microorganism(s) for which the animals will be monitored and screened. The length of the incubation period may pose significant challenges. Therefore, it is very important to implement a robust health-monitoring programme at the supplier’s farm or facility. At the end of the quarantine period, the animals found positive for the disease agents which are on the exclusion list should be taken out of the user establishment (except for some specific cases in which treatment could be considered, such as some types of parasitic infestation). Efforts should be undertaken to bring them back to the supplier or to find any other farm or use for them (before they are culled). The cadavers should be disposed of in a way that avoids spreading disease agents and is compliant with national legislation. When judged appropriate (depending on the incubation time of the disease), the quarantine period might be extended. In the case of unexpected deaths or unplanned euthanasia, diagnostic necropsy is very important and informative.^40^

### 5.2. Personnel

The importance of an experienced, competent attending veterinarian who has clinical experience with the species of interest and is aware of the requirements arising from the research programme is paramount for the optimal functioning of the facility.^41,42^ All personnel working in the facility should be able to recognise clinical signs of disease, pain and distress, assess emergencies and have adequate knowledge and understanding of the species involved and the hygienic management of the research facility. They should receive appropriate initial training with regular refreshers, in which the attending veterinarian should be actively involved. Animal health problems should be reported immediately to the attending veterinarian on call, while inherent logistical or organisational problems should be reported to the manager of the structure. Both the attending veterinarian and the animal facility manager are responsible for solving the reported problems in a timely manner. Regular, frequent interactions and good, clear communication between researchers, animal facility personnel and the attending veterinarian are very important for good functioning of the research facility. All personnel should understand the need to comply with the facility’s hygiene rules and to use adequate personal protective equipment, which they should agree to use and be able to use it correctly.

### 5.3. Health management programme

The health management programme depends largely on the type of study performed and on its duration (acute or chronic studies), and it should be designed in a way that makes it cross-applicable with the one at the supplier’s facility. The main points to be considered are listed below (taking into account the involvement of all personnel described in the previous paragraphs).

#### 5.3.1. Risk assessment and mitigation

It is essential to evaluate the risks of introducing agents through inanimate vectors (material) or live vectors (pests, other animals, humans) and to establish control policies for these risks. For example:

1. Adequate facility engineering controls should be commensurate with the risks (air pressure hierarchy, air filtration, provisions for cleaning and disinfecting holding rooms and equipment, access facilities such as air locks and provisions to change into protective clothing).
2. Personnel protective equipment should be available at all times.
3. Special precautions should be taken (communicated, understood and implemented) by employees having livestock at home (hygiene – showering, dedicated clothing for work).
4. A clear visitor access policy should be in place. Hygienic and quarantine rules should be communicated in advance, and written records should be kept (signed documentation that the facility’s rules have been understood and adhered to by all visitors, visitors’ book or similar documentation for tracking traffic and contamination risks). Registration of persons and also vehicles that enter and exit the premises may be a national regulatory requirement.

#### 5.3.2. Monitoring of the animals

1. Regular clinical health checks of the animals should be performed (using individual clinical score sheets).^43^
2. Periodic screening should be done to ensure that the animals enrolled in the study remain free from unwanted agents (that the inclusion and exclusion criteria are still met). Diagnostics on diseased animals or unexpected deaths should also provide information for the monitoring programme.
3. If the end of the study coincides with the sacrifice of the animal, necropsy should be performed. Relevant gross lesions and histopathology findings should be reported and taken into consideration when drawing the conclusions. Additional tests might be needed for diagnostic procedures.

#### 5.3.3. Contingency plan

1. A predefined action plan is needed in case of unexpected positive results, depending on their impact on people and animals (zoonoses), studies (interfering agents, major pathogens) and facility (outbreaks of notifiable diseases) – for example, retest (confirm the results), inform all stakeholders, including relevant authorities, separate the animals, sacrifice, decontaminate, environmental sampling and repopulation.
2. A disaster plan is required in case of physical damage to the facility that requires transfer of the animals to other premises.

## Supplemental Material

sj-pdf-1-lan-10.1177_0023677220944461 - Supplemental material for Federation of European Laboratory Animal Science Associations recommendations of best practices for the health management of ruminants and pigs used for scientific and educational purposes Click here for additional data file. Supplemental material, sj-pdf-1-lan-10.1177_0023677220944461 for Federation of European Laboratory Animal Science Associations recommendations of best practices for the health management of ruminants and pigs used for scientific and educational purposes by FELASA Working Group on Farm Animals: Corina Mihaela Berset (Convenor), Maria Emiliana Caristo, Fabienne Ferrara, Patrick Hardy, Marianne Oropeza-Moe and Ryan Waters in Laboratory Animals

sj-pdf-2-lan-10.1177_0023677220944461 - Supplemental material for Federation of European Laboratory Animal Science Associations recommendations of best practices for the health management of ruminants and pigs used for scientific and educational purposes Click here for additional data file. Supplemental material, sj-pdf-2-lan-10.1177_0023677220944461 for Federation of European Laboratory Animal Science Associations recommendations of best practices for the health management of ruminants and pigs used for scientific and educational purposes by FELASA Working Group on Farm Animals: Corina Mihaela Berset (Convenor), Maria Emiliana Caristo, Fabienne Ferrara, Patrick Hardy, Marianne Oropeza-Moe and Ryan Waters in Laboratory Animals

sj-pdf-3-lan-10.1177_0023677220944461 - Supplemental material for Federation of European Laboratory Animal Science Associations recommendations of best practices for the health management of ruminants and pigs used for scientific and educational purposes Click here for additional data file. Supplemental material, sj-pdf-3-lan-10.1177_0023677220944461 for Federation of European Laboratory Animal Science Associations recommendations of best practices for the health management of ruminants and pigs used for scientific and educational purposes by FELASA Working Group on Farm Animals: Corina Mihaela Berset (Convenor), Maria Emiliana Caristo, Fabienne Ferrara, Patrick Hardy, Marianne Oropeza-Moe and Ryan Waters in Laboratory Animals

sj-pdf-4-lan-10.1177_0023677220944461 - Supplemental material for Federation of European Laboratory Animal Science Associations recommendations of best practices for the health management of ruminants and pigs used for scientific and educational purposes Click here for additional data file. Supplemental material, sj-pdf-4-lan-10.1177_0023677220944461 for Federation of European Laboratory Animal Science Associations recommendations of best practices for the health management of ruminants and pigs used for scientific and educational purposes by FELASA Working Group on Farm Animals: Corina Mihaela Berset (Convenor), Maria Emiliana Caristo, Fabienne Ferrara, Patrick Hardy, Marianne Oropeza-Moe and Ryan Waters in Laboratory Animals

sj-pdf-5-lan-10.1177_0023677220944461 - Supplemental material for Federation of European Laboratory Animal Science Associations recommendations of best practices for the health management of ruminants and pigs used for scientific and educational purposes Click here for additional data file. Supplemental material, sj-pdf-5-lan-10.1177_0023677220944461 for Federation of European Laboratory Animal Science Associations recommendations of best practices for the health management of ruminants and pigs used for scientific and educational purposes by FELASA Working Group on Farm Animals: Corina Mihaela Berset (Convenor), Maria Emiliana Caristo, Fabienne Ferrara, Patrick Hardy, Marianne Oropeza-Moe and Ryan Waters in Laboratory Animals

sj-pdf-6-lan-10.1177_0023677220944461 - Supplemental material for Federation of European Laboratory Animal Science Associations recommendations of best practices for the health management of ruminants and pigs used for scientific and educational purposes Click here for additional data file. Supplemental material, sj-pdf-6-lan-10.1177_0023677220944461 for Federation of European Laboratory Animal Science Associations recommendations of best practices for the health management of ruminants and pigs used for scientific and educational purposes by FELASA Working Group on Farm Animals: Corina Mihaela Berset (Convenor), Maria Emiliana Caristo, Fabienne Ferrara, Patrick Hardy, Marianne Oropeza-Moe and Ryan Waters in Laboratory Animals

sj-pdf-7-lan-10.1177_0023677220944461 - Supplemental material for Federation of European Laboratory Animal Science Associations recommendations of best practices for the health management of ruminants and pigs used for scientific and educational purposes Click here for additional data file. Supplemental material, sj-pdf-7-lan-10.1177_0023677220944461 for Federation of European Laboratory Animal Science Associations recommendations of best practices for the health management of ruminants and pigs used for scientific and educational purposes by FELASA Working Group on Farm Animals: Corina Mihaela Berset (Convenor), Maria Emiliana Caristo, Fabienne Ferrara, Patrick Hardy, Marianne Oropeza-Moe and Ryan Waters in Laboratory Animals

sj-pdf-8-lan-10.1177_0023677220944461 - Supplemental material for Federation of European Laboratory Animal Science Associations recommendations of best practices for the health management of ruminants and pigs used for scientific and educational purposes Click here for additional data file. Supplemental material, sj-pdf-8-lan-10.1177_0023677220944461 for Federation of European Laboratory Animal Science Associations recommendations of best practices for the health management of ruminants and pigs used for scientific and educational purposes by FELASA Working Group on Farm Animals: Corina Mihaela Berset (Convenor), Maria Emiliana Caristo, Fabienne Ferrara, Patrick Hardy, Marianne Oropeza-Moe and Ryan Waters in Laboratory Animals

sj-pdf-9-lan-10.1177_0023677220944461 - Supplemental material for Federation of European Laboratory Animal Science Associations recommendations of best practices for the health management of ruminants and pigs used for scientific and educational purposes Click here for additional data file. Supplemental material, sj-pdf-9-lan-10.1177_0023677220944461 for Federation of European Laboratory Animal Science Associations recommendations of best practices for the health management of ruminants and pigs used for scientific and educational purposes by FELASA Working Group on Farm Animals: Corina Mihaela Berset (Convenor), Maria Emiliana Caristo, Fabienne Ferrara, Patrick Hardy, Marianne Oropeza-Moe and Ryan Waters in Laboratory Animals

sj-pdf-10-lan-10.1177_0023677220944461 - Supplemental material for Federation of European Laboratory Animal Science Associations recommendations of best practices for the health management of ruminants and pigs used for scientific and educational purposes Click here for additional data file. Supplemental material, sj-pdf-10-lan-10.1177_0023677220944461 for Federation of European Laboratory Animal Science Associations recommendations of best practices for the health management of ruminants and pigs used for scientific and educational purposes by FELASA Working Group on Farm Animals: Corina Mihaela Berset (Convenor), Maria Emiliana Caristo, Fabienne Ferrara, Patrick Hardy, Marianne Oropeza-Moe and Ryan Waters in Laboratory Animals

sj-pdf-11-lan-10.1177_0023677220944461 - Supplemental material for Federation of European Laboratory Animal Science Associations recommendations of best practices for the health management of ruminants and pigs used for scientific and educational purposes Click here for additional data file. Supplemental material, sj-pdf-11-lan-10.1177_0023677220944461 for Federation of European Laboratory Animal Science Associations recommendations of best practices for the health management of ruminants and pigs used for scientific and educational purposes by FELASA Working Group on Farm Animals: Corina Mihaela Berset (Convenor), Maria Emiliana Caristo, Fabienne Ferrara, Patrick Hardy, Marianne Oropeza-Moe and Ryan Waters in Laboratory Animals

sj-pdf-12-lan-10.1177_0023677220944461 - Supplemental material for Federation of European Laboratory Animal Science Associations recommendations of best practices for the health management of ruminants and pigs used for scientific and educational purposes Click here for additional data file. Supplemental material, sj-pdf-12-lan-10.1177_0023677220944461 for Federation of European Laboratory Animal Science Associations recommendations of best practices for the health management of ruminants and pigs used for scientific and educational purposes by FELASA Working Group on Farm Animals: Corina Mihaela Berset (Convenor), Maria Emiliana Caristo, Fabienne Ferrara, Patrick Hardy, Marianne Oropeza-Moe and Ryan Waters in Laboratory Animals
