# Supplementary material for: Federation of European Laboratory Animal Science Associations recommendations of best practices for the health management of ruminants and pigs used for scientific and educational purposes
Source: Lab Anim. 2020 Aug 9;55(2):117–28. doi: 10.1177/0023677220944461 (PMC8044623; doi:10.1177/0023677220944461)
Supplement: sj-pdf-1-lan-10.1177_0023677220944461 - Supplemental material for Federation of European Laboratory Animal Science Associations recommendations of best practices for the health management of ruminants and pigs used for scientific and educational purposes [file sj-pdf-1-lan-10.1177_0023677220944461.pdf]

Appendix 1. Legal requirements and recommendations

**Animal welfare (scientific purposes)**
Directive 2010/63/EU on the protection of animals used for scientific purposes *
European Convention ETS 123 (1986) for the protection of vertebrate animals used for experimental and other scientific purposes *
Appendix A (2006) of the European Convention ETS 123 for the protection of vertebrate animals used for experimental and other scientific purposes – Guidelines for accommodation and care of animals (article 5 of the Convention) *
Guide for the Care and Use of Laboratory Animals, 8th edition, National Research Council (2011)
Guide for the Care and Use of Agricultural Animals in Research and Teaching Federation of Animal Science Societies, 3rd edition (2010)
The UFAW Handbook on the Care and Management of Laboratory and Other Research Animals, 8th edition (2010)
Handbook Good Laboratory Practice, quality practices for regulated non-clinical research and development, 2nd edition (2009), Special Programme for Research and Training in Tropical Diseases
**Animal welfare (agriculture or general)**
Directive 98/58/EC concerning the protection of animals kept for farming purposes
Council Directive 2008/119/EC of 18 December 2008 laying down minimum standards for the protection of calves
Council Directive 2008/120/EC laying down minimum standards for the protection of pigs
Commission decision amending the Annex to Directive 91/629/EEC laying down minimum standards for the protection of calves
Regulation (EC) N° 1099/2009 on the protection of animals at the time of killing
Commission decision (1999) concerning minimum requirements for the inspection of holdings on which animals are kept for farming purposes
European Convention ETS 87 for the Protection of animals kept for farming purposes (reflecting the 5 Freedoms)
The 5 Freedoms of Animal Welfare (1979, UK Farm Animal Welfare Council), adopted by OIE in its Terrestrial Animal Health Code and European Convention ETS 87
OIE Terrestrial Animal Health Code (2018) Volume 1 – section 7 – Animal Welfare
European Commission Directorate D (animal health and welfare) - General guidance on EU import and transit rules for live animals and animal<br>products from third countries (for information purposes only, has not been<br>adopted or approved by the European Commission)
European Food Safety Authority (EFSA) - Scientific advice on all aspects of animal diseases and well-being of food producing-animals during breeding, rearing, transportation and slaughter (https://www.efsa.europa.eu/en/topics/topic/animal-health-and-welfare)
**Animal health (agriculture)**
Council Directive 82/894/EEC of 21 December 1982 on the notification of animal diseases within the Community (end of validity on 20/04/2021, repealed and replaced by Regulation (EU) 2016/429
Regulation (EU) 2016/429 on transmissible animal diseases and amending and repealing certain acts in the area of animal health (‘Animal Health Law’)
OIE list of notifiable animal disease (listed diseases, infections & infestations in force (current annual revision)
OIE Terrestrial Animal Health Code (2018)
European Commission Directorate D (animal health and welfare) - General guidance on EU import and transit rules for live animals and animal products from third countries (for information purposes only, has not been adopted or approved by the European Commission)

**Animal transport (health)**
Directive 90/425/EEC concerning veterinary and zootechnical checks applicable in intra-Community trade in certain live animals and products with a view to the completion of the internal market
Directive 92/65/EEC laying down animal health requirements governing trade in and imports into the Community of animals, semen, ova and embryos not subject to animal health requirements laid down in specific Community rules referred to in Annex A (I) to Directive 90/425/EEC
Regulation (EC) N° 1282/2002 amending Annexes to Council Directive 92/65/EEC laying down animal health requirements governing trade in and imports into the Community of animals, semen, ova and embryos not subject to animal health requirements laid own in specific Community rules referred to in Annex A(1) to Directive 90/425/EEC
Directive 2004/68/EC laying down animal health rules for the importation into and transit through the Community of certain live ungulate animals, amending Directives 90/426/EEC and 92/65/EEC and repealing Directive 72/462/EEC
Commission implementing decision (2012) amending Annex E to Directive 92/65/EEC as regards the model health certificates for animals from holdings and animals, semen, ova and embryos from approved bodies, institutes or centers
General guidance on EU import and transit rules for live animals and animal products from third countries (2009)
Commission Decision 003/24/EC (2002) concerning the development of an integrated computerized veterinary system
Commission Decision 2003/623/EC (2003) concerning the development of an integrated computerized veterinary system known as TRACES
TRACES (TRAde Control & Expert System) - European Commission's multilingual online management tool for all sanitary requirements on intra-EU trade and importation of animals, semen and embryo (common veterinary entry documents for animals and products entering the European Union & intra-EU trade certificates for animals and products)
**Animal transport (welfare)**
Regulation (EC) n° 1255/97 concerning Community criteria for staging points and amending the route plan referred to in the Annex to Directive 91 /628/EEC
Regulation (EC) No 1040/2003 amending Regulation (EC) No 1255/97 as regards the use of staging points
Regulation (EC) N° 1/2005 on the protection of animals during transport and related operations, amending Directives 64/432/EEC and 93/119/EC and Regulation (EC) N° 1255/97
Convention ETS 65 for the Protection of Animals during International Transport amended by ETS 103 (additional protocol to the European Convention for the Protection of Animals during International Transport)
OIE Terrestrial Animal Health Code (2018) Volume 1 - section 7 – Animal Welfare
**Biotechnology products from animal origin**
EMEA CPMP/ICH/295/95 - ICH harmonized tripartite guidelines, viral safety evaluation if biotechnology products derived from cell lines or human if animal origin, Q5A(R1) current Step 4 version (1999)
EMA/CHMP/BWP/457920/2012 rev 1 - Guideline on the use of bovine serum in the manufacture of human biological medicinal products (2013)
**Health & Safety**
Directive 2000/54/EC on the protection of workers from risks related to exposure to biological agents at work
WHO Laboratory Biosafety Manual, 3rd edition (2004)
Occupational Health and Safety in the Care and Use of Research Animals, National Research Council (1997)

* Implementation of Directive 2010/63/EU and Convention ETS 123 is highly recommended in Europe, even when not applicable to the animal use.
