# Supplementary material for: Federation of European Laboratory Animal Science Associations recommendations of best practices for the health management of ruminants and pigs used for scientific and educational purposes
Source: Lab Anim. 2020 Aug 9;55(2):117–28. doi: 10.1177/0023677220944461 (PMC8044623; doi:10.1177/0023677220944461)
Supplement: sj-pdf-2-lan-10.1177_0023677220944461 - Supplemental material for Federation of European Laboratory Animal Science Associations recommendations of best practices for the health management of ruminants and pigs used for scientific and educational purposes [file sj-pdf-2-lan-10.1177_0023677220944461.pdf]

Appendix 2. Roles and responsibilities in the user establishment

Task	Responsible persons
**Defining components of a health management programme**	
Health status (exclusion list)	Veterinarian & Study Direction
Health management	Veterinarian & Facility Manager
Health monitoring	Veterinarian & Facility Manager
**Programme management & implementation**	
Programme management	Facility Manager
Programme implementation	Animal Technicians<br>Study Directors<br>Research Technicians
**Management & execution of health monitoring**	Veterinarian
	Veterinary Technicians
**Decision making**	
Positive, negative results or re-test	Veterinarian
Study invalidation / recycling	Veterinarian<br>Study Directors
Recycling plan	Veterinarian & Facility Manager
