# Supplementary material for: Federation of European Laboratory Animal Science Associations recommendations of best practices for the health management of ruminants and pigs used for scientific and educational purposes
Source: Lab Anim. 2020 Aug 9;55(2):117–28. doi: 10.1177/0023677220944461 (PMC8044623; doi:10.1177/0023677220944461)
Supplement: sj-pdf-3-lan-10.1177_0023677220944461 - Supplemental material for Federation of European Laboratory Animal Science Associations recommendations of best practices for the health management of ruminants and pigs used for scientific and educational purposes [file sj-pdf-3-lan-10.1177_0023677220944461.pdf]

Appendix 3. Quality and Technical Agreement

Appendix 3.1. Example of table of content

1 Purpose
1.1 Scope
1.2 Duration
1.3 Confidentiality - Restriction of use

2 Service definition

3 Applicable guidelines and standards

4 Quality management system
4.1 Deviation management and investigations
4.2 Change control
4.3 Documentation and records
4.4 Outcome of regulatory inspections
4.5 Archives (documents and samples)
4.6 Audits and reciprocal responsibilities

5 Personnel and HSE
5.1 Personnel resources, organization chart, competence, roles and responsibilities
5.2 Health, Safety and Environment

6 Physical plant and equipment
6.1 General requirements
6.2 Room allocation and flows
6.3 Physical barriers and processes
6.4 Heating, ventilation and air conditioning,
6.5 Regulation, monitoring and alarms
6.2 Cleaning, disinfection a waste management
6.3 Facility, equipment and utility qualification
6.4 Maintenance and calibration
6.5 Records
6.6 Business continuity plan / disaster plan

7 Breeding & animal care
7.1 Procurement & storage
7.2 Genetic and breeding programme
7.3 Zootechnical monitoring and reports (e.g. production parameters, breeding performance)
7.4 Health status and programme
7.5 Health monitoring (veterinary diagnostics, outcome of abbatoir inspections and necropsy findings)
7.6 Animal welfare
7.7 Veterinary care (prophylactic or therapeutic)
7.8 Age and batch definition / identification
7.9 Rejection of animals
7.10 Stock and shipment preparation

8 Delivery
8.1 Orders
8.2 Delivery / batch identification
8.3 Vehicles and packaging
8.4 List of documents
8.5 Delivery terms

9 Checks upon reception

10 Post-delivery issues

11 Failure of compliance to specifications

12 Complaint management

13 Conflict resolution

14 Responsibilities

15 History of the document / revisions

16 Signatures

17 Appendixes

Appendix 3.2. Combining the QTA and health status definition

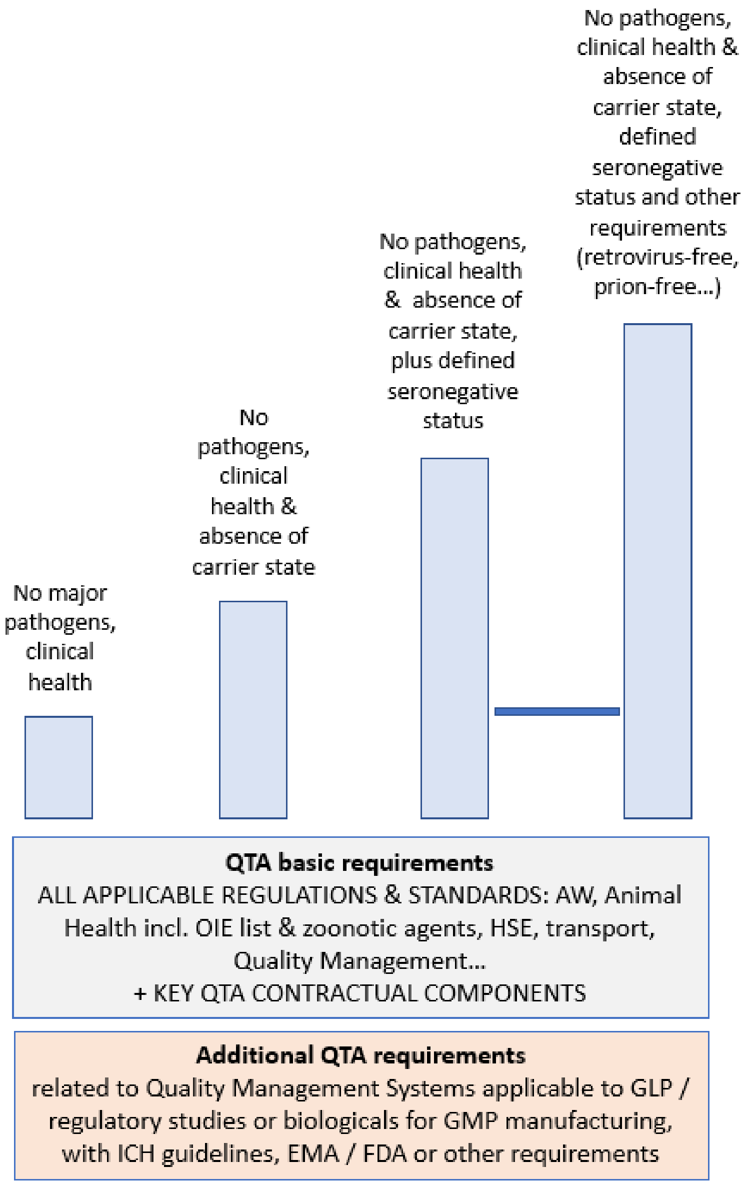

AW: Animal Welfare, EMA: European Medicines Agency, FDA: Food and Drug Administration, GLP: Good Laboratory Practice, GMP: Good Manufacturing Practice, HSE: Health and Safety Executive, ICH: International Council for Harmonisation of Technical Requirements for Pharmaceuticals for Human Use, OIE: World Organisation for Animal Health, QTA: Quality and Technical Agreement.
