# Supplementary material for: Federation of European Laboratory Animal Science Associations recommendations of best practices for the health management of ruminants and pigs used for scientific and educational purposes
Source: Lab Anim. 2020 Aug 9;55(2):117–28. doi: 10.1177/0023677220944461 (PMC8044623; doi:10.1177/0023677220944461)
Supplement: sj-pdf-4-lan-10.1177_0023677220944461 - Supplemental material for Federation of European Laboratory Animal Science Associations recommendations of best practices for the health management of ruminants and pigs used for scientific and educational purposes [file sj-pdf-4-lan-10.1177_0023677220944461.pdf]

Appendix 4. Herd health plan and farm biosecurity policy

4.1. Herd health plan

The farms - as other animal suppliers - should have a health management programme which should be agreed between the farm and the veterinarian. This programme is unit-specific, with any vaccination regimen, metaphylactic treatments, and adherence to any farm based/national health screening/eradication policy documented. It should also fulfil the following requirements (Figure A):

a. To be periodically reviewed and, if necessary, revised by the veterinarian, in close collaboration with the facility manager.
b. To be reviewed / revised rapidly in the event of substantial changes to husbandry practices or abnormal zootechnical performances.
c. To keep track of health and zootechnical performances records including meat/milk inspection outcomes etc.
d. To include a strategy for the prevention and control of common diseases and a suitable antiparasitic programme.
e. To set health and husbandry procedures covering the whole production cycle.
f. To require an immediate action plan to tackle injuries or clinical signs.
g. To include and monitor recommendations expressed during welfare assessments.
h. To keep records of staff competence, including for individuals who are deemed competent by a veterinarian to perform prescribed treatments.
i. To be available to all staff having a responsibility for animals.
j. To ensure a full compliance with national and international animal health regulations, e.g. in the event of an outbreak of a notifiable animal disease.

Farms are food-production businesses, which means that any additional time/cost not contributing to its profitability will most likely not be implemented, unless it is rewarded by the user establishment.
When identifying a supplier, defining the pre-existing animal health and herd management will help identify and manage the risk of incursions of new organisms into the farm.

The main information collected and documented should include:

a. vaccination strategy, including type of vaccines
b. ecto- and endoparasite treatment (considering the potential development of resistance)
c. any other prophylactic / metaphylactic treatments applied
d. disease occurrences, at least over the past 18 months
e. any disease from which the animals have been deemed free by being member of an accreditation scheme
f. open / closed herd and level of engineering / procedural controls in place to assure biosecurity

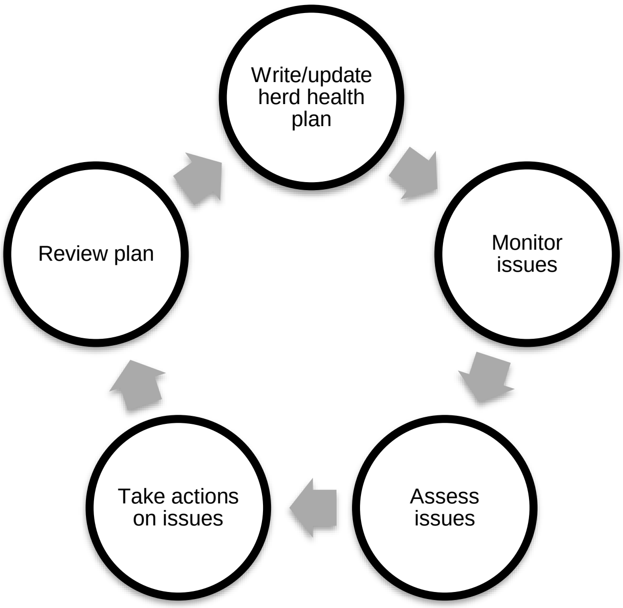

Figure A. *Establishment and maintenance of a herd health plan, aiming to proactively manage and improve health and welfare of livestock. The farmer and the farm's veterinarians should work closely together on this plan and collaborate with the user establishment (request for input, timely communication)*

4.2. Farm biosecurity policy

The herd health plan should contain a documented farm biosecurity policy that also outlines quarantine procedures and includes on-site related processes and to control risks to the farm including:

a. incoming stock (bought-in animals, sperm, embryos and relevant biologicals; and livestock that have been on grass-keep etc.)
b. potential disease transmission from animal to animal within the holding
c. diet, water, bedding (especially important when natural raw materials such as hay are fed or used for bedding, but also industrially produced diets). When drinking water is sourced from natural sources rather that public water supply, its quality should be considered)
d. equipment and vehicles
e. pest management
f. external visits/ contact of farm owner and personnel
g. wildlife (for example, using double fencing)
h. neighboring farms (both sided - communication is very important)

The farm biosecurity programme should also provide written records on washing and disinfection processes (for boots, clothing, vehicles, equipment and rooms available for use by staff and visitors), regular antiparasitic treatments of farm dogs (in accordance with the manufacturers' recommendations), grazing intervals (especially where manure is spread on grazing land).
