# Supplementary material for: Federation of European Laboratory Animal Science Associations recommendations of best practices for the health management of ruminants and pigs used for scientific and educational purposes
Source: Lab Anim. 2020 Aug 9;55(2):117–28. doi: 10.1177/0023677220944461 (PMC8044623; doi:10.1177/0023677220944461)
Supplement: sj-pdf-5-lan-10.1177_0023677220944461 - Supplemental material for Federation of European Laboratory Animal Science Associations recommendations of best practices for the health management of ruminants and pigs used for scientific and educational purposes [file sj-pdf-5-lan-10.1177_0023677220944461.pdf]

Appendix 5. Example of agents for pigs

Infectious/ parasitic agent	Body system	Transmission route	Incubation period	Important clinical signs	Triggered by stress (Yes/ No)	Immunosuppressive/ chronic	Important lesions at necropsy	Prophylactic disease control measures	Zoonosis (Yes/ No)	References
**Bacteria**										
Actinobacillus pleuro-pneumoniae (APP)	CV/HP, RES	APP resides in the respiratory tract of pigs. Introduction of subclinically affected carrier animals to naïve herds. Vertical transmission, piglets in close contact to sows' nasal discharge	Variable, depending on the strain virulence, co-infections, infectious dose, and managemental aspects	Pathogenic serotypes differ between countries. Sudden death, hyperthermia (41.5 °C) coughing, cyanosis, dyspnea, mouth breathing, dog-sitting posture, anorexia	Yes	The chronic form will lead to no or little hyperthermia, intermittent coughing and possibly reduced feed intake contributing to reduced weight gain	Fibrinous or fibrous epi- and pericarditis. Firm, rubbery lungs with friable necrotic areas, pleuropneumonia, sequestra, fibrous adhesions (pleura parietalis and visceralis)	Antimicrobial treatment of sick pigs. Bacterin and subunit toxin-vaccines. Depopulation and repopulation with disease-free pigs	No	1-3
Bordetella bronchiseptica	RES	*B. bronchiseptica* infects swine and a wide range of wild and domesticated mammals. Transmission via aerosol droplets and airborne spread has been described. Cross-species transmission may occur. *B. bronchiseptica* persists in the nasal cavity for several months	Two to three days in uncomplicated disease	Clinical signs mainly in 3-4-weeks-old pigs, or around weaning. Sneezing and snuffling, catarrhal rhinitis	Yes	*B. bronchiseptica* can predispose to secondary bacterial infections by means that remain unknown, but disturbance of the innate protective mechanisms during colonization of the nasal mucosa and ciliated cells is likely to play an important role	May progress to progressive athrophic rhinitis. Suppurative bronchopneumonia in young piglets	Combination of managemental, environmental and antimicrobial measures. Vaccines	Yes	4-11
Brachyspira hyodysenteriae	D	*B. hyodysenteriae* infects swine and occasionally birds (rheas, ducks, chicken and geese). It has been identified in rats, mice, dogs and feral birds on infected farms. Transmission mainly occurs by ingestion of	Incubation periods vary from 2 days to 3 months. Usually disease occurs within 10-14 days in naturally exposed pigs. Spirochetes are found in the feces 1 to 4	Clinical signs mainly in grower and finisher pigs, less frequently in weaners. Mild hyperthermia (40.0-40.5 °C), yellow to gray feces with mucus and flecks of blood, then watery stools with blod, mucus and	Overcrowding and temperature fluctuations may precipitate disease	Pigs recovering from *B. hyodysenteriae* infection show reduced growth rates	Hyperemia and edema of the large intestine wall and mesentery, swollen mesenteric lymph nodes, mucofibrinous pseudomembranes	Antimicrobial treatment (pleuromutilin resistance occurs). Eradication protocol	No	12-16

		contaminated fecal material	days before diarrhea starts	mucofibrinous exudate						
Brucella suis	CV/ HP, D, I, NL, REP, U	Feral swine, wild boars and wild hares pose a risk for domestic swine. Transmission between wild boars to pigs is thought to be the venereal route, not however excluding other routes. Lateral transmission can be through direct contact, aerosols or ingestion of infected fetuses or placental membranes	Highly variable.	Abortions, increased perinatal mortality, return to estrus and infertility. Swollen joints in surviving piglets and lameness. Enlarged or atrophic nodular testicles and/ or epididymides. Posterior paralysis and spondylitis has been described	No	Granulomas can be found in lymphatic tissues.	Granulomas with caseous centers; mammary glands, placentas, synovial membranes, seminal vesicles, prostate, epididymides, testes, uterus, oviducts, liver, spleen, bones, tendons, bursae, brain and lymph nodes. Placentitis, embryonic death	Well-established biosecurity protocols to avoid contact with infected wildlife reservoirs or contaminated material.	Yes	17, 18
Campylobacter jejuni, *C. coli*, *C. hyointestinals* and *C. mucosalis*	D	Ubiquitous worldwide. Direct transmission from one pig to another through the oral route or indirectly through contaminated water or feces. Birds, rodents and insects can introduce infection	One to three days	Campylobacters have not been associated with generally recognized enteric syndromes in swine. Clinical signs rarely ascribed to the infection are mucoid creamy diarrhea which can contain blood in piglets from 3 days to 3 weeks of age. Inflammatory reactions in the ileum and large intestine can occur in weaned piglets	No	No	Slight gross changes can be found in the small intestine of suckling piglets; thickening of the ileum especially the terminal segment. Mesenteric lymph nodes are prominent. Mucoid contents can be found in the ileum and/ or caecum. Stunting of the ileal villi, enlargement of lymphoid tissue in the terminal ileum	Control has rarely, if ever, been attempted in pig herds. Hygiene and chlorination of water supplies would be an appropriate method. Rodents, birds and insects should be excluded, if reduction of infection is an objective	Yes. Campyloba cters are the most common cause of food-borne bacterial enteric disease in humans	19-21
Clostridium perfringens type A enteritis	D	Sows are the probable source of infection in young suckling piglets	Within 48 hours of birth, piglets develop clinical sighs	Creamy or pasty diarrhea within 48 hours of birth, displaying rough hair coat and perineal fecal staining	No	No	Flaccid small intestine filled with gas and watery contents. Mild inflammation of the mucosa	Vaccination available in some countries	No	22, 23
Clostridium perfringens type C	D	Sows' feces are the probable source of infection in young suckling piglets	Short, as early as 12-36 hours postpartum piglets can be	Peracute infection: sudden deaths without clinical signs, hemorrhagic	No	Surviving piglets can develop chronic infection but unthriftiness often results in euthanasia	Hemorrhagic small intestines, bloodstained fluid in abdominal cavity.	Toxoid vaccines for sows to accomplish passive immunization of suckling piglets. Systematic wash and	No	22, 24, 25

			found dead in the pens	diarrhea, depression, hypothermia. unthriftiness. Acute infection: Reddish-brown diarrhea, ehydration, anorexia, depression. Subacute infection: Progessive emaciation, yellow stools with necrotic debris. Chronic infection: intermittent diarrhea			Necrotic, often segmental enteritis with emphysema of the intestinal wall, peritonitis. Fibrinous peritonitis	disinfection routines should be implemented		
Erysipelothrix rhusiopathiae	CV/HP, NL, IN, REP	*E. rhusiopathiae* is ubiquitous. Numerous species are known to harbor the organism, including pigs. Carriers harbor *E. rhusiopathiae* in the tonsils and other lymphoid tissues. Feces and oronasal secretions are sources of infection	The acute form or septicemic disease shows a sudden onset	Hyperthermia (40-42 °C), anorexia, reluctance to rise, stiff walk and lameness	Yes	Yes	Skin discoloration, diamond-shaped skin lesions. Vegetative valvular endocarditis	Vaccines are available. Antimicrobial treatment; preferably penicillin. Eradication: cesarean derivation or medicated early weaning combined with strict biosecurity measures	Yes	26-29
*Escherichia coli (*F5 (K99), F6 (987P), F41, F4 (K88), AIDA*,* F18)	D	Sources of infection are feces, water, soil and the environment as such in the pen	Variable. Neonatal diarrhea can be observed as early as 2-3 hours after birth	Mild to profuse diarrhea, eventually hemorrhagic	Yes	No	Dehydration, dilation of the stomach that can contain undigested milk, venous infarcts of the greater curvature of the stomach. Edema disease will cause vasculitis and edema subcutaneously, in the submucosa of the gastric cardia, the mesocolon, the mesentery of the jejunum and sometimes the gallbladder	Vaccination of sows prior to farrowing, vaccination of piglets to avoid post weaning diarrhea or edema disease. Good hygiene, plentiful supply of colostrum, organic acids, spray-dried plasma, probiotics and zinc oxide (ZnO)	Yes (STEC)	30-36
Haemophilus parasuis	CV/HP, NL	The bacterium belongs to the normal respiratory microbiota of pigs.	Variable	Affects mainly 4 to 8 week old pigs; sudden death, high hyperthermia (41.5	Yes	Chronic cases show rough hair, reduced growth rates and lameness	Fibrinous to fibrinopurulent polyserositis	Antimicrobial treatment and vaccination. Reduce stress levels	No	37-39

		Pigs are colonized during the suckling period		°C), coughing, abdominal breathing, swollen joints, lameness, central nervous signs						
Leptospira sp.	CV/HP, D, REP, U	Transmission is thought to occur via the mucous membranes of the eye, mouth or nose. The vaginal route and through milk has been demonstrated experimentally	Variable	Most infections are subclinical. Piglets may show anorexia, hyperthermia and listlessness	No	Chronic listerioses can cause considerable losses due to increased numbers of abortions, stillbirths, weak born piglets and reduced litter size	Jaundice and hemoglobinuria. Renal, tubular damage, focal liver necrosis and lymphocytic infiltration of adrenal glands, meningoencephalitis with perivascular lymphocytic infiltration. Abortions, stillbirths and weak born piglets. Bloodstained fluid in body cavities, petechial hemorrhages in the renal cortex of fetuses	Antimicrobial therapy, vaccination and management. Vaccination is not available in many Western European countries. Prevention of direct or indirect contact with free-living vectors or other domestic stock. Strict biosecurity measures	Yes	40-44
Methicillin resistant *Staphylococcus aureus* (MRSA)	RES, D	Transmission occur through direct contact or through the environment	MRSA colonizes the pigs, typically without causing disease	Colonisation of nasal mucosa of pigs that can constitute a separate MRSA reservoir. Colonisation does not give rise to any disease in most cases	No	No	Usually none	Strict biosecurity measures can prevent introducing MRSA to a naïve pig herd. Surveillance programs in Norway have minimized occurrence of MRSA in the pig population	Yes	45-47
Mycoplasma hyopneumoniae	RES	Nose-to-nose contact with carrier pigs, airborne transmission	Variable	Endemic mycoplasmosis is more common; insidious onset with dry, non-productive cough, fever, decreased appetite, labored breathing or prostration due to secondary pathogens. Epizootic mycoplasmosis: in naïve herds;	Yes	*M. hyopneumoniae* is considered a primary pathogen very much involved in the porcine respiratory disease complex (PRDC). The bacterium causes modulations of both the innate and adaptive immune responses	Consolidation of cranioventral lung portions, catarrhal exudate. Secondary infections complicate pathological lesions	All in- all out management, medicated, segregated early weaning, multisite operations, eradication of the infectious agent or vaccination	No	48-51

				coughing, respiratory distress, pyrexia and death						
Mycoplasma hyorhinis	CV/ HP, D, NL RES,	*M. hyorhinis* is a normal member of the upper airways of young pigs. Direct contact cause transmission between animals	Three to ten days	Polyserositis typically occurs in 3-10-week-old pigs. Slight hyperthermia, depression, reluctance to move, difficulty breathing, conjunctivitis, otitis, lameness and swollen joints	Yes	Probably immunosuppressive. Chronic infection cause fibrous adhesions leading to reduced weight gain and feed conversion rates	Fibrinopurulent pericarditis, pleuritis and sometimes peritonitis. Fibrous adhesions can develop. Serosanguinous and fibrinous synovial fluid	Stress prevention can prevent disease outbreaks.	No	52-57
Mycoplasma hyosynoviae	NL	The bacterium colonize the respiratory tract of pigs and it persists in tonsils. Transmission occurs from sows to piglets at 4 to 8 weeks of age	Four to nine days	Clinical lameness typically occurs in 3-5-month-old pigs, sometimes in more than one leg. No hyperthermia. Lameness. Joint swelling may be present	Yes	In chronic phases, the joint membranes may be thickened due to fibrosis	Proliferation, swelling, edema and hyperemia of the synovial membranes in affected joints. Increased volumes of serofibrinous to serosanguineous synovial fluid. Periarticular edema is common. Fibrosis of joint membranes with pannus formation	Antimicrobial treatment, stress prevention can prevent disease outbreaks. No commercial vaccine is available	No	58, 59
Pasturella multocida (toxin producing)	CV/HP, RES	Present in the majority of pig herds. Aerosol and nose-to-nose transmission	Variable	PAR: Sneezing, epistaxis, brachygnatia superior in 4 to 12 weeks old pigs, coughing, intermittent hyperthermia, anorexia, labored breathing	Yes	Progressive atrophic rhinitis can be seen from 4 to 12 weeks of age	Brachygnatia superior in 4 to 12 weeks old pigs, turbinate atrophy, suppurative bronchopneumonia	Antimicrobial treatment<br>Vaccination<br>Eradication protocol	Yes	7, 60, 61
Salmonella sp.	CV/HP, D	Fecal-oral transmission is the most likely mode of transmission of virulent salmonellae. Transmission can occur through contaminated environment, dam to offspring or between	Variable.	*S. choleraesuis*: often pigs less than five months of age; septicemia, inappetence, hyperthermia, cough, icterus.<br>*S. typhimurium*: Yellow diarrhea, hyperthermia,	Yes.	Chronic salmonellosis caused by *S. typhimurium* may lead to wasting, rectal strictures and obstipation because of fibrosis due to ischemia in the rectum with its precarious blood supply	*S. choleraesuis*: Cyanosis, enlarged lymph nodes, necrotic foci in liver, renal cortical petechial and ecchymoses.<br>*S. typhimurium*: Focal or diffuse necrotic enteritis,	Antimicrobial treatment is often challenging due to plasmid-mediated resistance. Preventive measures should be advocated. Husbandry routines must be optimized. Carrier pigs, contaminated feed or environment are	Yes	62-67

		pigs. Short distance aerosol transmission is probable		decreased feed intake, dehydration			colitis or typhlitis. Adherent gray-yellow debris on roughened mucosal surface, button ulcers. Enlarged mesenteric lymph nodes	significant sources of infection. Some countries have surveillance programs and implement eradication programs whenever *Salmonella sp.* is detected		
Staphylococcus hyicus	IN	*St. hyicus* is present worldwide at pig facilities. Inoculation during birth in the vagina is suspected as a means of vertical transmission. Fighting or biting can lead to infections as well as high humidity and poor ventilation	Piglets from 3 to 4 days of age can show clinical signs	Clinical signs in piglets between 4-6 days and 5-6 weeks of age. Reddish skin color, brown scales, ulcers and separation of claw horn at heel bulbs. Rapid weight loss may occur and death. No pruritus, hyperthermia is not common	Yes	Chronically affected animals are stunted, reduced weight gain can result from infection	Intradermal vesicular and pustular dermatitis, acanthosis, perivascular inflammation	Fluid replacer and antimicrobial treatment in early stages of disease. Vaccination of sows with autogenous bacterins	Yes	68-70
β-hemolytic streptococci and *Streptococcus suis*	CV/HP, NL	Healthy carrier animals transmit virulent strains between herds. Sows infect piglets during farrowing. Aerosol transmission without nose-to-nose contact has been confirmed for S. suis serotype 2	Variable. Clinical signs are normally seen between 6 and 10 weeks of age	Generally pigs between 5 and 10 weeks of age; fluctuating hyperthermia, anorexia, depression and lameness	Yes	Chronic disease can cause wasting.	Vegetative valvular endocarditis, myocarditis, rhinitis and bacteremia/ septicemia	Management improvement. Antimicrobial treatment; preferably amoxicillin. Vaccines Eradication protocol	Yes	27, 71-73
**Viruses**										
African swine hyperthermia virus (ASFV)	CV/HP, D, I, RES, U,	ASFV is endemic in more than 20 countries in sub-Saharan Africa. Currently several European countries are also struggling with ASFV. The virus has also reached China. Direct transmission by contact between domesticated pigs	Four to nineteen days	Sudden death, hyperthermia, anorexia, leukopenia, skin hemorrhages	No	In endemic situations, subacute or chronic forms may be present	Skin hemorrhages, hemorrhages in multiple inner organs, splenomegaly	Eradication protocol. No vaccine available	No	74-77

		and wild boars is the most common route								
Encephalomyo-carditis virus	CV/HP, REP	Widespread geographical distribution, many susceptible hosts. Direct pig-to-pig contact is a potential transmission route. Transplacental transmission may occur. Infectious rodent carcasses pose a risk	Variable	Sudden death in young pigs and reproduction disorders in sows	No	Chronic myocarditis can occur	Epicardial hemorrhage, hydropericardium, hydrothorax, pulmonary edema. The heart muscle is often enlarged, soft and pale. Multiple foci of various sizes (circular and linear, gray-white in color) are found in the myocardium	Stand-empty period followed by thorough cleaning and disinfection. Strict rodent control	No	78, 79
Porcine circovirus 2 (PCV2)	CV/HP, D, I, IN, REP, RES, U	Oronasal exposure is the primary route of transmission. However, PCV2 has been found in nasal, tonsillar, bronchial and ocular secretions, feces, saliva, urine, colostrum, milk and semen	Variable	Subclinical infections may occur. Reproductive failure in sows and myocarditis in aborted and/ or mummified fetuses. Wasting, pallor of the skin, respiratory distress and diarrhea or icterus in pigs from 2 to 4 months of age. PCV2-associated enteritis can occur.<br>In nursery to adult pigs, anorexia, depression	Yes	The virus is immunosuppressive and chronic disease lead to wasting and poor production results	Lymphoid depletion in lymph nodes. Proliferative and necrotizing pneumonia. Granulomatous enteritis. Porcine dermatitis and nephropathy syndrome (PDNS): red-to-purple macules and papules in the skin on hind limbs and perineal area can occur. Here, enlarged kidneys with pinpoint cortical lesions and edema of the renal pelvis can be found. Mummifications, stillborn piglets	Vaccination control program	No	80-85
Porcine Epidemic Diarrhoea virus (PEDV)	D	Direct or indirect fecal-oral transmission	Four to five days	PEDV can infect pigs of all ages, causing depression, anorexia, diarrhea and vomiting.	No	Recurrent weaning diarrhea may occur after an acute outbreak has passed	Distended stomach filled with completely undigested milk curd and thin, transparent intestine walls with accumulation of yellowish fluids.	Strict biosecurity to avoid entrance of PEDV into pig farms by minimizing introduction of any material or any person, which could be in contact with the virus.	No	86-90
Porcine parvovirus (PPV)	REP	PPV can be transported between herds via fomites	Variable	Usually subclinical infection of pregnant dams, but can show	No	No	Offspring may be mummified, stillborn or weak. .	Vaccination of gilts and sows	No	91, 92

		and rodents (mechanical vectors) and infected boars		return to estrus, fail to farrow or farrow only few piglets. born			Congestion, edema and hemorrhage with accumulation of serosanguineous fluids in body cavities, dehydration			
Porcine reproductive and respiratory syndrome virus (PRRSV)	CV/HP, IN, REP, RES,	Parenteral exposure through breaks in the skin barrier, oral fluids, blood, insects, airborne transmission. Vertical transmission	Variable	High mortality when introduced to naïve herd, anorexia and lethargy, reproductive failure, respiratory signs	No	The virus is immunosuppressive.	Cutaneous hyperemia or cyanosis. Perivascular myocarditis. Stillborn or mummified fetuses	Eradication program of positive herd by e.g. segregated early weaning or test and removal. Gilt acclimatization routines in PRRSV positive herds. Vaccines to moderate clinical signs and reduce virus shedding	No	93-95
Rotavirus	D	Fecal-oral route.	Eighteen to ninety six hours	Listlessness, anorexia, vomiting, yellow-to-white flocculent diarrhea in suckling and recently weaned piglets	No	No	Dehydration, thin-walled jejunum, flaccid and dilated with watery, flocculent, yellow or gray fluid. The lacteals in the distal parts of the intestine contain no chyle	General supportive therapy and antimicrobial treatment to prevent secondary bacterial infections. Electrolyte solution containing glucose-glycine minimize dehydration and weight loss. Active immunization of sows to protect offspring from infection during the suckling period	Yes	96-98
Swine influenza virus (SIV), all subtypes	RES	Introduction of infected animals to a herd, pig-to-pig contact via nasopharyngeal exposure. Aerosol transmission	Short	Hyperthermia, anorexia, prostration, huddling, conjunctivitis, rhinitis, nasal discharge, sneezing, coughing, dyspnea	VI, IF, IHC, ELISA		Sharp demarcation of affected, purple, firm tissue and normal lung tissue. Bronchial and mediastinal lymph node enlargement. Blood-tinged fibrinous exudates in airways. Necrosis of lung epithelia and desquamation of bronchial epithelial cells	Vaccination and biosecurity measures.	Yes	99-101
Transmissible gastroenteritis virus (TGEV)	D	Seasonal appearance during winter. Cats, dogs and foxes are possible carriers of TGEV. Starlings can mechanically spread	Eighteen hours to three days	Piglets < 2 weeks of age: Vomiting, profuse watery, yellowish diarrhea, with rapid weight loss, dehydration,	No	Endemic Transmissible Gastroenteritis occur in large herds farrowing frequently where mortality is low and clinical signs	Distended stomach with curdled milk sometimes with petechial hemorrhages. The jejunum is distended	Provide a warm (above 32°C), draft-free, dry environment and provide water or nutrient solutions freely to	No	102-104

		TGEV between farms. Possibly transfer between farms due to long-term carrier pigs		and high morbidity and mortality. Most suckling pigs over 3 weeks of age will survive, but may remain stunted. Clinical signs of TGE in finishing swine and in sows include inappetence, transient diarrhea, and vomiting		can resemble rotavirus infection	with yellow fluid and undigested milk. The wall is thin and the villi are markedly shortened in the jejunum and ileum	TGEV-infected pigs. Elimination of TGEV		
**Parasites**										
Ascaris suum	D, RES	The most common nematode in pigs. Contact with ascarid eggs normally starts when neonates come in contact with the ground		Often subclinical infections, however, reduced growth rates can occur	No	Chronic infection can affect weight gain	Mild eosinophilic infiltrations in liver and lymphoid nodules in hepatic portal areas. Verminous pneumonia may result in deaths if large amounts of larvae invade the lungs at the same time. Intestinal obstipation can result after occlusion of the intestinal lumen by adult worms	Anthelmintics. Thorough wash and disinfection between batches	Yes	105-107
Isospora suis	D	Coccidia are ubiquitous anywhere pigs are raised in confinement		In piglets between 7 - 11 days to 5-6 weeks, yellowish to grayish diarrhea occurs. Rough hair coat, reduced weight gain	No	No	Enteric villous atrophy, villous fusion, crypt hyperplasia, and necrotic enteritis	Good sanitation, thorough cleaning of the crates to remove organic debris, disinfection, and steam cleaning. In extreme cases, sealing or painting solid surfaces within farrowing crates can help break the cycle of reinfection by the hardy oocysts	No	108
Sarcoptes scabiei	IN	Worldwide distribution. Contact between sows (main reservoir) and other pigs		Generalized pruritus and encrusted lesions	No	Chronic hyperkeratotic form	Encrusted lesions especially in the inner surface of the ears, focal erythematous skin papules, hyperkeratosis	Treatment with acaricides and good sanitation, good nutrition and good management. Eradication	Yes	109, 110

Trichuris suis	D	Fecal-oral transmission		Bloody diarrhea, enterocyte destruction, loss of capillary blood	No		Light, minimal lesions in the cecum or ulceration of the mucosa, mucosal edema, hemorrhage and formation of a fibrinonecrotic membrane	Thorough wash and disinfection between batches, anthelmintic treatment	No	111, 112

Body systems; CV/ HP: Cardiovascular and hematopoietic system, D: Digestive system, I: Immune system, IN: Integumentary system; skin, hoof and claw, NL: Nervous and locomotor systems, REP: Reproductive system, RES: Respiratory system, U: Urinary system

References

1. Gottschalk M, Lacouture S. *Actinobacillus pleuropneumoniae* serotypes 3, 6, 8 and 15 isolated from diseased pigs in North America. *Vet Rec* 2014; 174: 452 https://doi.org/10.1136/vr.102470

2. MacInnes JI, Gottschalk M, Lone AG, et al. Prevalence of *Actinobacillus pleuropneumoniae*, *Actinobacillus suis*, *Haemophilus parasuis*, *Pasteurella multocida*, and *Streptococcus suis* in representative Ontario swine herds. *Can J Vet Res* 2008; 72: 242–248.

3. Sassu EL, Bossé JT, Tobias TJ, et al. Update on *Actinobacillus pleuropneumoniae* - knowledge, gaps and challenges. *Transbound Emerg Dis* 2017; 65, 72–90.

4. Baars JC, De Jong MF, Storm PK, et al. Atrophic rhinitis and its control with an adjuvant vaccine consisting of *B. bronchiseptica* and *P. multocida* strains. In: *Proc Int Congr Pig Vet Soc*, International congress of the pig veterinary society (IPVS), Mexico, p. 121.

5. Brockmeier SL, Register, KB, Nicholson, TL, et al. Bordetellosis. In: Zimmerman et al. (eds.) *Diseases of Swine*. 10th ed., West Sussex, UK: Wiley-Blackwell, 2012, pp. 670–679.

6. Brockmeier, SL, Lager, KM. Experimental airborne transmission of porcine reproductive and respiratory syndrome virus and *Bordetella bronchiseptica*. *Vet Microbiol* 2002; 89: 267–275.

7. Chanter N, Magyar T, Rutter JM. Interactions between *Bordetella bronchiseptica* and toxigenic *Pasteurella multocida* in atrophic rhinitis of pigs. *Res Vet Sci* 1989; 47: 48–53. https://doi.org/10.1016/S0034-5288(18)31230-X

8. Coutts AJ, Dawson S, Binns S, et al. Studies on natural transmission of *Bordetella bronchiseptica* in cats. *Vet Microbiol* 1996; 48: 19–27.

9. Farrington DO, Jorgenson RD. Prevalence of *Bordetella bronchiseptica* in certain central Iowa. *J Wildl Dis* 1976; 12: 523–525.

10. Goodnow RA. Biology of *Bordetella bronchiseptica*. *Microbiol Rev* 1980; 44: 722–738.

11. Pedersen KB, Barfod, K. The aetiological significance of *Bordetella bronchiseptica* and *Pasteurella multocida* in atrophic rhinitis of swine. *Nord Vet Med*1981; 33: 513–522.

12. Alvarez-Ordóñez A, Martínez-Lobo FJ, Arguello H, et al. Swine Dysentery: Aetiology, Pathogenicity, Determinants of Transmission and the Fight against the Disease. *Int J Environ Res Public Health* 2013; 10: 1927–1947. https://doi.org/10.3390/ijerph10051927

13. Burrough ER. Swine Dysentery: Etiopathogenesis and Diagnosis of a Reemerging Disease. *Vet Pathol* 2017; 54: 22–31. https://doi.org/10.1177/0300985816653795

14. Hampson DJ. Brachyspiral Colitis. In: Zimmerman et al. (eds.) *Diseases of Swine*. 10th ed., West Sussex, UK: Wiley-Blackwell, 2012, pp. 680–696.

15. Joerling J, Barth SA, Schlez K, et al. Phylogenetic diversity, antimicrobial susceptibility and virulence gene profiles of *Brachyspira hyodysenteriae* isolates from pigs in Germany. PLOS ONE 2018: 13, e0190928. https://doi.org/10.1371/journal.pone.0190928

16. Kinyon JM, Harris DL, Glock RD. Enteropathogenicity of various isolates of *Treponema hyodysenteriae*. *Infect Immun* 1977; 15: 638–646.

17. Enright FM, Araya LN, Elzer PH, et al. Comparative histopathology in BALB/c mice infected with virulent and attenuated strains of *Brucella abortus*. *Vet Immunol Immunopathol* 1990; 26: 171–182.

18. Olsen SC, Garin-Bastuji B, Blasco JM et al. Brucellosis. In: Zimmerman et al. (eds.) *Diseases of Swine*. 10th ed., West Sussex, UK: Wiley-Blackwell, 2012, pp. 697–708.

19. Soultos N, Madden RH. A genotyping investigation of the colonization of piglets by *Campylobacter coli* in the first 10 weeks of life. *J Appl Microbiol.* 2007; 102: 916–920. https://doi.org/10.1111/j.1365-2672.2006.03158.x

20. Taylor DJ. *Pig diseases*, 9th ed. Lennoxtown, Glasgow, Great Britain: Wayment Print & Publishing Solutions Ltd., 2013.

21. Taylor DJ,. Miscellaneous Bacterial Infections. In: Zimmerman et al. (eds.) *Diseases of Swine*. 10th ed., West Sussex, UK: Wiley-Blackwell, 2012, pp. 866–881.

22. Songer JG, 2012. Clostridiosis. In: Zimmerman et al. (eds.) *Diseases of Swine*. 10th ed., West Sussex, UK: Wiley-Blackwell, 2012, pp. 709–722.

23. Songer JG, Uzal FA. Clostridial enteric infections in pigs. *J Vet Diagn Investig Off Publ Am Assoc Vet Lab Diagn Inc* 2005; 17: 528–536. https://doi.org/10.1177/104063870501700602

24. Schäfer K, Wyder M, Gobeli S, et al. Detection of *Clostridium perfringens* type C in pig herds following disease outbreak and subsequent vaccination. *Vet Rec* 2012; 171: 503–503. https://doi.org/10.1136/vr.101052

25. Wollschläger N, Zimmermann W, Brodard I, et al. Occurrence of *Clostridium perfringens* type A and type C in piglets of the Swiss swine population. *Schweiz Arch Tierheilkd* 2009; 151: 377–382. https://doi.org/10.1024/0036-7281.151.8.377

26. Eamens GJ, Chin JC, Turner B, et al. Evaluation of *Erysipelothrix rhusiopathiae* vaccines in pigs by intradermal challenge and immune responses. *Vet Microbiol* 2006; 116: 138–148. https://doi.org/10.1016/j.vetmic.2006.03.018

27. Loynachan AT. 2012. Cardiovascular and haemopoietic system. In: Zimmerman et al. (eds.) *Diseases of Swine*. 10th ed., West Sussex, UK: Wiley-Blackwell, 2012, pp. 189–198.

28. Stephenson EH, Berman, DT. Isolation of *Erysipelothrix rhusiopathiae* from tonsils of apparently normal swine by two methods. *Am J Vet Res* 1978; 39: 187–188.

29. Wang Q, Chang BJ, Riley TV. *Erysipelothrix rhusiopathiae*. *Vet Microbiol Zoonoses: Advances and Perspectives* 2010; 140: 405–417. https://doi.org/10.1016/j.vetmic.2009.08.012

30. Blázquez E, Rodríguez C, Ródenas J, et al. Evaluation of ultraviolet-C and spray-drying processes as two independent inactivation steps on enterotoxigenic *Escherichia coli* K88 and K99 strains inoculated in fresh unconcentrated porcine plasma. *Lett Appl Microbiol* 2018; 67: 442–448. https://doi.org/10.1111/lam.13068

31. Fairbrother JM, Nadeau É, Bélanger L, et al.. Immunogenicity and protective efficacy of a single-dose live non-pathogenic *Escherichia coli* oral vaccine against F4-positive enterotoxigenic *Escherichia coli* challenge in pigs. *Vaccine* 2017; 35: 353–360. https://doi.org/10.1016/j.vaccine.2016.11.045

32. Fairbrother JM, Gyles CL, 2012. Colibacillosis. In: Zimmerman et al. (eds.) *Diseases of Swine*. 10th ed., West Sussex, UK: Wiley-Blackwell, 2012, pp. 723–749.

33. Johansen M, Andresen LO, Jorsal SE, et al. Prevention of edema disease in pigs by vaccination with verotoxin 2e toxoid. *Can J Vet. Res* 1997; 61: 280–285.

34. Lu X, Zhang M, Zhao L, et al. Growth Performance and Post-Weaning Diarrhea in Piglets Fed a Diet Supplemented with Probiotic Complexes. *J Microbiol Biotechnol* 2018; 28: 1791–1799. https://doi.org/10.4014/jmb.1807.07026

35. Nadeau É, Fairbrother JM, Zentek J, et al. Efficacy of a single oral dose of a live bivalent *E. coli* vaccine against post-weaning diarrhea due to F4 and F18-positive enterotoxigenic *E. coli*. *Vet J* 2017; 26: 32–39. https://doi.org/10.1016/j.tvjl.2017.07.004

36. Oropeza-Moe M, Grøntvedt CA, Phythian CJ, et al.. Zinc oxide enriched peat influence *Escherichia coli* infection related diarrhea, growth rates, serum and tissue zinc levels in Norwegian piglets around weaning: five case herd trials. *Porc Health Manag* 2017a; 3: 14. https://doi.org/10.1186/s40813-017-0060-7

37. Bello-Orti B, Costa-Hurtado M, Martinez-Moliner V, et al. Time course *Haemophilus parasuis* infection reveals pathological differences between virulent and non-virulent strains in the respiratory tract. *Vet Microbiol* 2014; 170: 430–437. https://doi.org/10.1016/j.vetmic.2014.01.011

38. Howell KJ, Weinert LA, Peters SE, et al. “Pathotyping” Multiplex PCR Assay for *Haemophilus parasuis:* a Tool for Prediction of Virulence. *J Clin Microbiol* 2017; 55: 2617–2628. https://doi.org/10.1128/JCM.02464-16

39. Howell KJ, Peters SE, Wang J, et al. Development of a Multiplex PCR Assay for Rapid Molecular Serotyping of Haemophilus parasuis. *J Clin Microbiol* 2015; 53: 3812–3821. https://doi.org/10.1128/JCM.01991-15

40. Chirathaworn C, Inwattana R, Poovorawan Y, et al. Interpretation of microscopic agglutination test for leptospirosis diagnosis and seroprevalence. *Asian Pac J Trop Biomed.* 2014; 4: S162–S164. https://doi.org/10.12980/APJTB.4.2014C580

41. De Azevedo J, Palmeiro JM. Contribution to the knowledge of leptospirosis in the domestic animals of Portugal. *J Med* (Oporto). 1961; 46: 497–511.

42. Langham RF, Morse EV, Morter RL. Experimental leptospirosis. V. Pathology of leptospira pomona infection in swine. *Am J Vet Res* 1958; 19: 395–400.

43. Nagy G. Comparative pathogenicity study of *Leptospira interrogans* serovar *pomona* strains. *Acta Vet Hung* 1993; 41: 315–324.

44. Strutzberg-Minder K, Tschentscher A, Beyerbach M, et al. Passive surveillance of *Leptospira* infection in swine in Germany. *Porc Health Manag* 2018; 4. https://doi.org/10.1186/s40813-018-0086-5

45. Khanna T, Friendship R, Dewey C, et al. Methicillin resistant *Staphylococcus aureus* colonization in pigs and pig farmers. *Vet Microbiol* 2008; 128: 298–303. https://doi.org/10.1016/j.vetmic.2007.10.006

46. Lewis HC, Mølbak K, Reese C, et al.. Pigs as Source of Methicillin-Resistant *Staphylococcus aureus* CC398 Infections in Humans, Denmark. *Emerg Infect Dis* 2008. 14, 1383–1389. https://doi.org/10.3201/eid1409.071576

47. Norwegian Veterinary Institute, Norwegian Food Safety Authority. *The surveillance programme for methicillin resistant Staphylococcus aureus in pigs in Norway 2017* (Annual Report). Norwegian Veterinary Institute, Norway, 2018a.

48. Holst S. Elimination of *Mycoplasma hyopneumoniae* from breed-to-wean farms: A review of current protocols with emphasis on herd closure and medication. *J Swine Health Prod* 2015; 23: 321–330.

49. Maes D, Segales J, Meyns T, et al.. Control of *Mycoplasma hyopneumoniae* infections in pigs. *Vet Microbiol* 2008; 126: 297–309. https://doi.org/10.1016/j.vetmic.2007.09.008

50. Maes D, Deluyker H, Verdonck M, et al. Effect of vaccination against *Mycoplasma hyopneumoniae* in pig herds with an all-in/all-out production system. *Vaccine* 1999; 17: 1024–1034.

51. Nathues H, Fournie G, Wieland B, et al. Modelling the within-herd transmission of *Mycoplasma hyopneumoniae* in closed pig herds. *Porc Health Manag* 2016; 2. https://doi.org/10.1186/s40813-016-0026-1

52. Caron J, Ouardani M, Dea S. Diagnosis and Differentiation of *Mycoplasma hyopneumoniae* and *Mycoplasma hyorhinis* Infections in Pigs by PCR Amplification of the p36 and p46 Genes. *J Clin Microbiol* 2000; 38: 1390–1396.

53. Fourour S, Tocqueville V, Paboeuf, F et al. Pathogenicity study of *Mycoplasma hyorhinis* and *M. flocculare* in specific-pathogen-free pigs pre-infected with *M. hyopneumoniae*. *Vet Microbiol* 2019; 232: 50–57. https://doi.org/10.1016/j.vetmic.2019.04.010

54. Friis NF. A serologic variant of *Mycoplasma hyorhinis* recovered from the conjunctiva of swine. *Acta Vet Scand* 1976; 17: 343–353.

55. Gomes Neto JC, Bower L, Erickson BZ, et al. Quantitative real-time polymerase chain reaction for detecting *Mycoplasma hyosynoviae* and *Mycoplasma hyorhinis* in pen-based oral, tonsillar, and nasal fluids. *J Vet Sci* 2015; 16: 195–201. https://doi.org/10.4142/jvs.2015.16.2.195

56. Ross R. Mycoplasmal diseases. In: Leman AD, Straw B and Mengeling WL (eds). *Diseases of Swine* Ames (IA): Iowa State, University Press, 2012, pp. 537–551

57. Ross RF, Young TF. The nature and detection of mycoplasmal immunogens. *Vet Microbiol* 1993; 37: 369–380.

58. Lauritsen KT, Heydenreich AV, Riber U, et al. Novel Vaccine Against Mycoplasma Hyosynoviae: *The Immunogenic Effect of Iscom-Based Vaccines in Swine*. Presented at the 3rd European Veterinary Immunology Workshop. 2009.

59. Nielsen EO, Nielsen NC, Friis NF. *Mycoplasma hyosynoviae* Arthritis in Grower-Finisher Pigs. *J Vet Med* 2001; Ser. A 48: 475–486. https://doi.org/10.1046/j.1439-0442.2001.00378.x

60. Davies RL, MacCorquodale R, Baillie S, et al. Characterization and comparison of *Pasteurella multocida* strains associated with porcine pneumonia and atrophic rhinitis. *J Med Microbiol* 2003; 52: 59–67. https://doi.org/10.1099/jmm.0.05019-0

61. Wilson BA, Ho M. *Pasteurella multocida*: from Zoonosis to Cellular Microbiology. *Clin Microbiol Rev* 2013; 26: 631–655. https://doi.org/10.1128/CMR.00024-13

62. Carlson SA, Barnhill AE, Griffith RW. 2012. Salmonellosis. In: Zimmerman et al. (eds.) *Diseases of Swine*. 10th ed., West Sussex, UK: Wiley-Blackwell, 2012, pp. 821–833.

63. Hurd HS, McKean JD, Griffith RW, et al. *Salmonella enterica* Infections in Market Swine with and without Transport and Holding. *Appl Environ Microbiol* 2002; 68: 2376–2381. https://doi.org/10.1128/AEM.68.5.2376-2381.2002

64. Lewerin SS, Skog L, Frössling J, et al. Geographical distribution of salmonella infected pig, cattle and sheep herds in Sweden 1993-2010. *Acta Vet Scand* 2011; 53: 51. https://doi.org/10.1186/1751-0147-53-51

65. Mousing J, Jensen PT, Halgaard C, et al. Nation-wide *Salmonella enterica* surveillance and control in Danish slaughter swine herds. *Prev Vet Med* 1997; 29: 247–261. https://doi.org/10.1016/S0167-5877(96)01082-3

66. Norwegian Veterinary Institute, Norwegian Food Safety Authority. *Surveillance programmes in Norway - Salmonella* - Annual Report 2017 (Annual Report). Norwegian Veterinary Instiute, Norway, 2018b.

67. Reed WM, Olander HJ, Thacker HL. Studies on the pathogenesis of Salmonella typhimurium and *Salmonella choleraesuis* var *kunzendorf* infection in weanling pigs. *Am J Vet Res* 1986; 47: 75–83.

68. Casanova C, Iselin L, von Steiger N et al. *Staphylococcus hyicus* bacteremia in a Farmer. *J Clin Microbiol* 2011; 49: 4377–4378. https://doi.org/10.1128/JCM.05645-11

69. Hunter D, Todd JN, Larkin M. Exudative epidermitis of pigs. The serological identification and distribution of the associated staphylococcus. *Br Vet J* 1970; 126: 225–229.

70. L'Ecuyer C, Jericho K. Exudative Epidermitis in Pigs. *Can J Comp Med Vet Sci* 1966; 30: 94–101.

71. Amass SF, SanMiguel P, Clark LK. Demonstration of vertical transmission of *Streptococcus suis* in swine by genomic fingerprinting. *J Clin Microbiol* 1997; 35: 1595–1596.

72. Berthelot-Hérault F, Gottschalk M, Labbé A, et al. Experimental airborne transmission of *Streptococcus suis* capsular type 2 in pigs. *Vet Microbiol* 2001; 82: 69–80.

73. Heidt MC, Mohamed W, Hain T, et al. Human Infective Endocarditis Caused by Streptococcus suis Serotype 2. *J Clin Microbiol* 2005; 43: 4898–4901. https://doi.org/10.1128/JCM.43.9.4898-4901.2005

74. Blome S, Gabriel C, Beer M. Pathogenesis of African swine fever in domestic pigs and European wild boar. *Virus Res* 2013; 173: 122–130. https://doi.org/10.1016/j.virusres.2012.10.026

75. Howey EB, O'Donnell V, de Carvalho Ferreira, HC, et al. Pathogenesis of highly virulent African swine fever virus in domestic pigs exposed via intraoropharyngeal, intranasopharyngeal, and intramuscular inoculation, and by direct contact with infected pigs. *Virus Res* 2013; 178: 328–339. https://doi.org/10.1016/j.virusres.2013.09.024

76. Sánchez-Cordón PJ, Montoya M, Reis AL, et al. African swine fever: A re-emerging viral disease threatening the global pig industry. *Vet J Lond Engl* 2018; 233: 41–48. https://doi.org/10.1016/j.tvjl.2017.12.025

77. Sánchez-Vizcaíno JM, Arias Neira M. African Swine Fever Virus. In: Zimmerman et al. (eds.) *Diseases of Swine*. 10th ed., West Sussex, UK: Wiley-Blackwell, 2012, pp. 396–404.

78. Maurice H, Nielen M, Brocchi E, et al. The occurrence of encephalomyocarditis virus (EMCV) in European pigs from 1990 to 2001. *Epidemiol Infect* 2005; 133: 547–557.

79. Vansteenkiste K, Van Limbergen T, Decaluwé R, et al. Clinical problems due to encephalomyocarditis virus infections in two pig herds. *Porc Health Manag* 2016: 2. https://doi.org/10.1186/s40813-016-0036-z

80. Brunborg IM, Jonassen CM, Moldal T, et al. Association of Myocarditis with High Viral Load of Porcine Circovirus Type 2 in Several Tissues in Cases of Fetal Death and High Mortality in Piglets. A Case Study. *J Vet Diagn Invest* 2007; 19: 368–375. https://doi.org/10.1177/104063870701900405

81. Larochelle R, Bielanski A, Müller P. et al. PCR Detection and Evidence of Shedding of Porcine Circovirus Type 2 in Boar Semen. *J Clin Microbiol* 2000; 38: 4629–4632.

82. Madson DM, Opriessnig T. Effect of porcine circovirus type 2 (PCV2) infection on reproduction: disease, vertical transmission, diagnostics and vaccination. *Anim Health Res Rev* 2011; 12: 47–65. https://doi.org/10.1017/S1466252311000053

83. Oropeza-Moe M, Oropeza Delgado AJ, Framstad T. Porcine circovirus type 2 associated reproductive failure in a specific pathogen free (SPF) piglet producing herd in Norway: a case report. *Porc Health Manag* 2017b; 3: 25. https://doi.org/10.1186/s40813-017-0072-3

84. Segalés J. Porcine circovirus type 2 (PCV2) infections: Clinical signs, pathology and laboratory diagnosis. *Virus Res*, *Recent Advances in Porcine Circoviruses and Associated Diseases* 2012; 164: 10–19. https://doi.org/10.1016/j.virusres.2011.10.007

85. West KH, Bystrom JM, Wojnarowicz C, et al. Myocarditis and abortion associated with intrauterine infection of sows with porcine circovirus 2. *J Vet Diagn Investig Off Publ Am Assoc Vet Lab Diagn Inc* 1999; 11: 530–532. https://doi.org/10.1177/104063879901100608

86. Boniotti MB, Papetti A, Lavazza A, et al. Porcine Epidemic Diarrhea Virus and Discovery of a Recombinant Swine Enteric Coronavirus, Italy. *Emerg Infect Dis* 2016; 22: 83–87. https://doi.org/10.3201/eid2201.150544

87. Hanke D, Pohlmann A, Sauter-Louis C, et al.. Porcine Epidemic Diarrhea in Europe: In-Detail Analyses of Disease Dynamics and Molecular Epidemiology. *Viruses* 2017; 9. https://doi.org/10.3390/v9070177

88. Kim Y, Lee C. Porcine epidemic diarrhea virus induces caspase-independent apoptosis through activation of mitochondrial apoptosis-inducing factor. *Virology* 2014; 460–461: 180–193. https://doi.org/10.1016/j.virol.2014.04.040

89. Lee C. Porcine epidemic diarrhea virus: An emerging and re-emerging epizootic swine virus. *Virol J* 2015; 12. https://doi.org/10.1186/s12985-015-0421-2

90. Lee S, Kim Y, Lee C. Isolation and characterization of a Korean porcine epidemic diarrhea virus strain KNU-141112. *Virus Res* 2015; 208: 215–224. https://doi.org/10.1016/j.virusres.2015.07.010

91. Joo HS, Donaldson-Wood CR, Johnson RH. Observations on the pathogenesis of porcine parvovirus infection. *Arch Virol* 1976; 51: 123–129. https://doi.org/10.1007/BF01317841

92. Van Leengoed LA, Vos J, Gruys E. Porcine Parvovirus infection: review and diagnosis in a sow herd with reproductive failure. *Vet Q* 1983; 5: 131–141. https://doi.org/10.1080/01652176.1983.9693887

93. Cano JP, Dee SA, Murtaugh MP et al. Impact of a modified-live porcine reproductive and respiratory syndrome virus vaccine intervention on a population of pigs infected with a heterologous isolate. *Vaccine* 2007; 25: 4382–4391. https://doi.org/10.1016/j.vaccine.2007.03.031

94. Kittawornrat A, Prickett J, Wang C, et al. Detection of Porcine reproductive and respiratory syndrome virus (PRRSV) antibodies in oral fluid specimens using a commercial PRRSV serum antibody enzyme-linked immunosorbent assay. *J Vet Diagn Investig Off Publ Am Assoc Vet Lab Diagn Inc.* 2012; 24: 262–269. https://doi.org/10.1177/1040638711435679

95. Lunney JK, Fang Y, Ladinig A, et al. Porcine Reproductive and Respiratory Syndrome Virus (PRRSV): Pathogenesis and Interaction with the Immune System. *Annu Rev Anim Biosci* 2016; 4: 129–154. https://doi.org/10.1146/annurev-animal-022114-111025

96. Crouch CF. Vaccination against enteric rota and coronaviruses in cattle and pigs: Enhancement of lactogenic immunity. *Vaccine* 1985; 3: 284–291. https://doi.org/10.1016/S0264-410X(85)90056-8

97. McAdaragh JP, Bergeland ME, Meyer RC, et al. Pathogenesis of rotaviral enteritis in gnotobiotic pigs: a microscopic study. *Am J Vet Res* 1980; 41:1572–1581.

98. Saif LJ, Fernandez FM. Group A rotavirus veterinary vaccines. *J Infect Dis* 1996; 174 Suppl 1: S98-106.

99. Grøntvedt CA, Er C, Gjerset B, et al. Clinical Impact of Infection with Pandemic Influenza (H1N1) 2009 Virus in Naïve Nucleus and Multiplier Pig Herds in Norway. *Influenza Res Treat* 2011, 163745. https://doi.org/10.1155/2011/163745

100. Haesebrouck F, Pensaert MB. Effect of intratracheal challenge of fattening pigs previously immunised with an inactivated influenza H1N1 vaccine. *Vet Microbiol* 1986; 11: 239–249.

101. Van Reeth K, Brown IH, Olsen CW. Influenza virus. In: Leman AD, Straw B and Mengeling WL (eds). *Diseases of Swine* Ames (IA): Iowa State, University Press, 2012, pp. 557–571.

102. Hooper BE, Haelterman EO. Lesions of the gastrointestinal tract of pigs infected with transmissible gastroenteritis. *Can. J. Comp. Med. Rev. Can. Med. Comp.* 1969; 33: 29–36.

103. Saif LJ, Pensaert MB, Sestak K, et al. Coronaviruses. In: Leman AD, Straw B and Mengeling WL (eds). *Diseases of Swine* Ames (IA): Iowa State, University Press, 2012, pp. 501–524.

104. Woods RD, Wesley RD. Transmissible gastroenteritis coronavirus carrier sow. *Adv Exp Med Biol* 1998; 440: 641–647. https://doi.org/10.1007/978-1-4615-5331-1_83

105. Katakam KK, Thamsborg SM, Dalsgaard A, et al. Environmental contamination and transmission of *Ascaris suum* in Danish organic pig farms. *Parasit Vectors* 2016: 9. https://doi.org/10.1186/s13071-016-1349-0

106. Menzies FD, Goodall EA, Taylor SM. The epidemiology of *Ascaris suum* infections in pigs in Northern Ireland, 1969–1991. *Br Vet J* 1994; 150: 165–172. https://doi.org/10.1016/S0007-1935(05)80224-6

107. Roepstorff A, Nansen P. Epidemiology and control of helminth infections in pigs under intensive and non-intensive production systems. Vet. Parasitol., Special Issue: 14th W.A.A.V.P. Conference 1994; 54: 69–85. https://doi.org/10.1016/0304-4017(94)90084-1

108. Lindsay DS, Dubey JP, Santín-Durán M, et al. Coccidia and other Protozoa. In: Leman AD, Straw B and Mengeling WL (eds). *Diseases of Swine* Ames (IA): Iowa State, University Press, 2012, pp. 895–907.

109. Grahofer A, Bannoehr J, Nathues H, et al. *Sarcoptes* infestation in two miniature pigs with zoonotic transmission – a case report. *BMC Vet Res* 2018; 14: 91. https://doi.org/10.1186/s12917-018-1420-5

110. Laha R. Sarcoptic mange infestation in pigs: an overview. *J Parasit Dis Off Organ Indian Soc Parasitol* 2015; 39: 596–603. https://doi.org/10.1007/s12639-014-0419-5

111. Bager P, Kapel C, Roepstorff A, et al. Symptoms after Ingestion of Pig Whipworm *Trichuris suis* Eggs in a Randomized Placebo-Controlled Double-Blind Clinical Trial. PLoS ONE 6, 2011. https://doi.org/10.1371/journal.pone.0022346

112. Thienpont D, Vanparijs O, Hermans L. Treatment of *Trichuris suis* infections in pigs with flubendazole. Vet Rec 1982; 110: 517–520.
