# Supplementary material for: Federation of European Laboratory Animal Science Associations recommendations of best practices for the health management of ruminants and pigs used for scientific and educational purposes
Source: Lab Anim. 2020 Aug 9;55(2):117–28. doi: 10.1177/0023677220944461 (PMC8044623; doi:10.1177/0023677220944461)
Supplement: sj-pdf-6-lan-10.1177_0023677220944461 - Supplemental material for Federation of European Laboratory Animal Science Associations recommendations of best practices for the health management of ruminants and pigs used for scientific and educational purposes [file sj-pdf-6-lan-10.1177_0023677220944461.pdf]

Appendix 6. Examples of agents for cattle

Infectious/ parasitic agent	Body system	Transmission route	Incubation period	Important clinical signs	Triggered by stress (Yes/ No)	Immunosuppressive / chronic	Important lesions at necropsy	Prophylactic disease control measures	Zoonosis (Yes/ No)	References
**Bacteria**										
Actinobacillus lignieresii («wooden tongue»)	D, IN	Direct infection of the soft tissues of the head (in particular tongue tissue) due to trauma from the environment via lingual trauma	Unknown	Inappetence, hollowed out appearance, salivation. Very firm, painful, swollen tongue. Occasionally soft tissue swellings of the other soft tissues in the head and neck may be seen.	No	Yes – chronic	Pyogranulomatous lesions seen in soft tissues of the head and neck	Not applicable	Possible but extremely uncommon	1
Actinomyces bovis («lumpy jaw»)	D, IN	Direct infection of the periostium due to trauma to the buccal cavity or via tooth eruption / infection	Unknown	Enlargement of mandible, discharging sinus, loss of milk production and body condition	No	Yes	Not applicable	Not applicable	Possible but extremely uncommon	2
Brucella abortus	REP	Direct contact with infected cattle	Unknown	Abortions are the main sign, with weak calves and fetal membrane retention	No - but carrier cattle shed high quantities of the bacterium at abortion	No, although bacteria are shed at calving	Not applicable	Vaccines available - can be combined with surveillance and stamping out to control disease. Some countries have implemented systems resulting in eradication	Yes	3
Campylobacter foetus	REP	Direct contact – venereal	Unknown	Infertility and abortions are commonly seen	No	No	Not applicable	Vaccines available in some countries and are very effective. Strict biosecurity and closed herds manage the risk of infection	Yes, along with other *Campylobacter* spp. strains which do not cause disease in cattle	4
Clostridium chauvoei («black leg»)	L	Direct infection from environment	3-10 days	Dull, depressed, lethargic, hyigh rectal temperature -	No	No	Extensive and obvious muscle necrosis, usually of a limb, is seen at	Vaccination against clostridial diseases most effective	No	5

				occasionally cattle found dead			necropsy. 10 months to 2 years age generally			
Clostridium novyi («black disease»)	D	Association with liver fluke infection	Not applicable	Rare - cattle usually found dead	No	No - although association with liver fluke	Pale areas of liver damage / infarcts are seen along with fluke damage	Effective fluke control along with vaccination against clostridial diseases	No	6
Clostridium tetani	L	Direct infection from environment via wounds	10-14 days	Visible wound (entry site), Bloat, stiffness, third eyelid protrusion, death	No	No	Not applicable	Elective procedures carried out in clean environment. Vaccine available	Yes	7
Coxiella burnetii	REP	Via aerosols, ingestion or direct contact. Persistent infections for several years, possibly lifelong. discharges during subsequent pregnancies and lactations. Long persistence in the environment; spread at long distances by the wind	28-56 days (experimental infection)	"Q Fever". Asymptomatic in nonpregnant animals; placentitis and abortions in the third or second trimester or stillbirths. Anorexia and depression 1-2 days before aborting. Abortions in successive parturitions possible	Yes, by stress, over-crowding, poor nutrition	Yes, chronic	The placenta with gross, white areas of necrosis; mineralization of the cotyledons and intercotyledonary area. Chorionic surface covered in thick exudate. Fetuses with no gross lesions	Culling the animals that serve as permanent reservoirs. Prompt incineration of placentas	Yes	8
Dichelobacter nodosus (interdigital dermatitis)	L	Indirect spread through environ-ment	Unknown	Limb discomfort, shifting weight initially, later animal avoids weight bearing on affected foot	No	No	Not applicable	Good housing systems keeping the feet dry and clean are of primary importance, regular foot care. Foot bathing.	No	9
Treponema spp. (digital dermatitis, «hairy wart»)	L	Indirect spread through environment	Unknown	Proliferative / wart like appearance ranging from mild to severe lameness in the flexure commissure of the interdigital space	No	No	Not applicable	Good housing systems keeping the feet dry and clean are of primary importance, as well as regular foot care. Foot bathing is also an option	No	10

Fusobacterium necrophorum - (interdigital necrobacillosis, «foot rot»)	L	Indirect spread through environ-ment	1 week	Swelling / erythema of interdigital space tissues and coronary band. Claws are typically markedly separated. Rapid onset of increasing lameness. Characteristic foul odor is produced	No	No	Not applicable	Good housing systems keeping the feet dry and clean are of primary importance, as well as regular foot care. Foot bathing is also an option	No	11
Mannheimia sp., *Histophilus* sp. and *Pasturella* sp.(bacterial pneumonia)	RES	Commensals	Not appli-cable	Coughing, dullness and depression, poor milk yield, fever (>41C), serous - mucopurulent ocular and nasal discharge, erythema / erosion of nasal mucosa. Loss of condition if chronic	Yes (and viral infection)	Yes, pneumonia can be chronic - these bacteria are, however, present in healthy animals as part of the normal microbiome	Hemorhaggic, fibrinonecrotic pneumonia with cranio-ventral areas of lung consolidation	Meta-<br>phylactic treatment with licensed antibiotics in high risk animals immediately before shipment in addition to ensuring immunity to RSV, IBV and PI3	No	11
Leptospira borgpetersenii spp. and *interrogans* spp. serovar *hardjo*	REP, U	Contact with infected urine or products of abortion	2-7 days	Infertility, abortion and poor milk yield	No	No	Not applicable	Effective vaccine is available	Yes	12
Moraxella bovis («pink eye»)	N	Dust / vector borne transmission	Unknown	Corneal ulceration, epiphora, partial blepharospasm	No	No	Not applicable	Dust and fly control - treatment of clinically affected animals with topical antibiotics	No	13
Mycobacterium avium paratuberculosis (Johne's disease)	D	Fecal - oral. Direct contact	Months - years	Progressive weight loss in the face of continued feeding. Profuse, watery "pipe stem" diarrhea may also be seen in chronic cases	No	Yes – chronic	Ileal mucosae thickened with corrugated appearance. Internal lymph nodes are enlarged	Testing and cull positive cases combined with strict on farm biosecurity is the main control measure	No	14
Mycobacterium bovis (bovine tuberculosis)	RES, D	Exposure to contaminated environment (from other infected cattle / wildlife)	Months - years	Progressive emaciation and lethargy are seen, with respiratory involvement presenting with a chronic moist cough	No	Yes – chronic	Granulomatous caseous lesions seen in lungs and other viscera	Many countries have national freedom and / or eradication programmes in place	Yes	15
S. typhimurium and *S.dublin* (Salmonellosis)	D	Fecal - oral infection	Either very short: 24-72 hours post exposure, either results from stressors	Diarrhea and abortion, occasional pyrexia and death due to dehydration / hypovolemia	Yes - carriers with no clinical signs	No	Not applicable	Vaccines available. Strict biosecurity and closed farms to reduce the risk of importing the bacteria onto the farm.	Yes	16

			in carrier animals					Screening and eliminating carriers possible.		
**Viruses**										
Bovine Ephemeral Fever Virus	L	Vector borne	1-14 days	Mild fever, joint pain / arthritis and muscle weakness / lameness. Sometimes anorexia, dullness and depression. Reduction in milk yield and reduced fertility	No	No	Not applicable	Herd immunity and controlling arthropod vectors	No	17
Bovine Herpesvirus-1 (Infectious Bovine Rhinotracheitis, BHV -1)	RES, REP	Inhalation - aerosols. Direct contact although many cattle have latent virus	2-6 days after exposure or 2-3 weeks following stressors in carrier animals. Secondary bacterial infection most important contributor to morbidity	Coughing, conjunctivitis, poor milk yield, fever (>41ºC), ocular and nasal discharge, erythema / erosion of nasal mucosa. Can also cause abortions. All ages susceptible	Yes - transport / mixing of animals and other stressors can cause the latent virus to become activated	No	Cattle which die - severe tracheitis	Vaccination available - some countries may have an eradication scheme, and closed herds can eradicate it	No	18
Bovine Leukaemia Virus (Enzootic Bovine Leucosis)	I	Vertical and horizontal (direct contact transmission)	2-5 weeks	Lymphadenopathy, weight loss / ill thrift	No	Yes	Internal lymphoid tumors, enlarged secondary lymphoid tissue	Many countries are free from the disease as a result of intense control programme - detect and destroy	No	19
Bovine papular stomatitis	D	Direct contact	Unknown	Usually inapparent - may be incidental finding. Shallow papules seen on the muzzle. Hard palate and gums	No	No	Usually seen in animals 1 year old or under	None. Self- limiting with no effect on productivity / welfare	Yes	20
Bovine parainfluenza virus 3 (PI3)	RES	Inhalation - aerosol. Direct contact	Unknown	Mild rhinitis when on its own, but significant respiratoy disease complex when present with BHV-1, BRSV or *Mannheimia* sp. and *Mycoplasma* sp.	Yes	No	As for bacterial pneumonia	Vaccination available	No	11

Bovine respiratory syncytial virus (BRSV)	RES	Inhalation - aerosol. Direct contact	2-6 days	Clinical signs variable, with some cattle infected with no clinical signs to those with severe presentations of respiratory distress and mouth breathing with mortality.	Yes	No	As for bacterial pneumonia	Vaccination available	No	11
Bovine viral diarrhoea virus (BVDV) (Acute BVD and Mucosal disease)	D	Fecal - oral. Direct contact	3-7 days	Acute infection may be inapparent or mild nasal discharge with transient pyrexia and diarrhea. During pregnancy may cause fetal death / abnormalities. Mucosal disease can cause sever ulceration of the mouth and muzzle, with anorexia, pyrexia and profuse diarrhea	No	Yes - transient with acute infection, ongoing with persistently infected animals.	Oedema and thickening of large intestine / corrugated appearance	Vaccine available - National control / eradication schemes are available	No	21
Coronavirus	D	Fecal - oral	Unknown	Similar to Rotavirus infection - dull, depressed, with mucoid diarrhea - seen in calves up to 3 weeks of age. Can result in death of severely affected and untreated calves	No	No	Not applicable	Vaccine available commonly with *E.coli* and Rotavirus	No	22
Foot-and-mouth disease virus (FMDV)	IN	Direct contact with infected animals and fomites	1-14 days	Vesicular lesions forming in / around oral cavity, coronary band, and interdigital space	No	No	Heart lesions (pale areas of myocardium) may be observed in young animals which died after infection	Vaccination available. Eradicated in the EU	No	23
Lumpy skin disease virus (LSDV)	IN	Vector borne	5-14 days	Pyrexia and inappetence, followed by well-defined circular nodules forming under / in the skin with associated enlargement of	No	Yes - chronic	Internal lymphadenopathy	Vaccine is available	No	24

				peripheral lymph nodes. Infertility, abortion and reduced milk yields are also recorded						
Ovine Herpes Virus 2 (malignant catarrhal fever)	RES	Contact with recently lambed or pregnant sheep	Days - months	Severe depression, pyrexia, loss of appetite and corneal opacity. Often accompanied by a muco-purulent nasal discharge	No	No	Systemic lymphadenopathy	No vaccine is available. Avoidance of contact with sheep is the best control measure	No	25
Rotavirus	D	Fecal - oral infection	1-7 days	Usually calves affected 5-14 days old, presenting with yellow diarrhea. Asymptomatic shedding in older cattle	No	No	Calves affected 5-14 days old: mild oedema of small intestine parenchyma – microscopic villus changes most prevalent.	Good hygiene, ample colostrum combined with vaccination of the dams	No	26
Vesicular stomatitis virus (VSV)	D	Direct contact / insect vectors	1-14 days	Oral vesiculation and ptyalism	No	No	Not applicable	OIE LIST PATHOGEN due to similarity with FMD	No	27
**Parasites**										
Anaplasma phagocytophilum (tick borne fever)	CV/HP, L	Vector borne (ticks)	7-60 days	Abortion, pyrexia, weight loss and severe drop in milk production	No	No - although carrier state possible.	Not applicable	Regular dipping of cattle to prevent the vector feeding; vaccines available in some countries	No	28
Babesia bovis /*B. divergens*	CV/HP	Vector borne (ticks)	1-4 weeks	Dark urine, loss of condition, mucosal palor	No	Chronic	Signs consistent with anemia	Regular dipping, tick eradication, vaccination	No	29

Cryptosporidum parvum	D	Fecal - oral	Unknown	Usually affects calves 1-4 weeks old. Diarrhea (watery), dullness, depression, lethargy	No	No	Not applicable	Herd immunity is critical, and therefore adequate colostrum intake combined with good environmental hygiene is essential	Yes	30
Dictyocaulus viviparus («lung worm / husk»)	RES	Indirect contact - ingestion of nematode from pasture (excreted by infected animal)	2-4 weeks	Marked weight loss, partial anorexia and tachypnoea. Later stages: persistent cough with the animals' head and neck extended when breathing	No	Chronic - recovered animals will have long term damage to lungs which may result in indefinite reductions in weight gain	Large numbers of larvae in the bronchi and bronchioles	Prophylactic antihelmintic treatment according to the herd health plan. An effective vaccine is also available	No	31
Eimeria spp. (coccidiosis)	D	Exposure to contaminated water courses / environment	3-4 weeks after mixing	Sudden onset; profuse mucoid diarrhea. Chronic wasting and poor appetite commonly observed	No	Yes	Not applicable	Coccidiostats can be used prophylactically and retrospectively, however unit hygiene is most important	No	32
Fasciola hepatica («liver fluke»)	D	Indirect - ingestion of infected snail	Highly variable: months-years	Reduced milk yeilds and reduced weight gains, poor fertility and marked weight loss in adult animals. Diarrhea may also be present	No	Yes - chronic infection	Visible fluke in liver at post mortem with fibrosed tracts	Prophylactic flukicide given to cattle as part of the herd health plan	Yes	33
Ostertagia ostertagi (ostertagiosis)	D	Indirect contact (fecal - oral)	Weeks - months	General loss of condition / ill thrift with significant scour / weight loss in type II disease	No	Yes - chronic infection	Oedema of gastrointestinal wall, thickening of abomasal wall	Prophylactic treatment with anthelmintic before turning out to clean pasture in line with herd health plan and lifecycle of organism in the region	No	34

Trichophyton verrucosum («ring worm»)	IN	Direct or indirect contact with infected animals and fomites	Unknown	Scaly gray skin lesions appear anywhere, but particularly around the head neck and rump	Yes - especially malnourished calves; self-limiting	No, but can be a sign of an underlying immunosuppression	Not applicable	Ensure immune-suppressive agents are not circulating on farm. Vaccines available	Yes	35

Body systems: CV/ HP: Cardiovascular and hematopoietic system, D: Digestive system, I: Immune system, IN: Integumentary system; skin, hoof and claw, N: Nervous system, L: Locomotor system, REP: Reproductive system, RES: Respiratory system, U: Urinary system

References

1. Rycroft AN and Garside LH. *Actinobacillus* species and their role in animal disease. *Vet J* 2000; 159: 18-36. DOI: 10.1053/tvjl.1999.0403.

2. Pine L, Howell A, Jr. and Watson SJ. Studies of the morphological, physiological, and biochemical characters of Actinomyces bovis. *J Gen Microbiol* 1960; 23: 403-424. DOI: 10.1099/00221287-23-3-403.

3. De Figueiredo P, Ficht TA, Rice-Ficht A, et al. Pathogenesis and immunobiology of brucellosis: review of Brucella-host interactions. *Am J Pathol* 2015; 185: 1505-1517. DOI: 10.1016/j.ajpath.2015.03.003.

4. Hoffer MA. Bovine campylobacteriosis: a review. *Can Vet J* 1981; 22: 327-330.

5. Uzal FA. Evidence-based medicine concerning efficacy of vaccination against *Clostridium chauvoei* infection in cattle. *Vet Clin North Am Food Anim Pract* 2012; 28: 71-77, viii. 2012/03/01. DOI: 10.1016/j.cvfa.2011.12.006.

6. Ditchfield J and Julian RJ. *Clostridium novyi* in Cattle. *Can Vet J* 1960; 1: 542.

7. Morrow DA. Tetanus in Cattle. *Cornell Vet* 1963; 53: 445-449.

8. Spickler AR. Q-Fever, http://www.cfsph.iastate.edu/DiseaseInfo/factsheets.php (2017, accessed 6th June 2019).

9. Wilson-Welder JH, Alt DP and Nally JE. The etiology of digital dermatitis in ruminants: recent perspectives. *Vet Med (Auckl)* 2015; 6: 155-164. DOI: 10.2147/VMRR.S62072.

10. Knappe-Poindecker M, Gilhuus M, Jensen TK, et al. Interdigital dermatitis, heel horn erosion, and digital dermatitis in 14 Norwegian dairy herds. *J Dairy Sci* 2013; 96: 7617-7629. DOI: 10.3168/jds.2013-6717.

11. Grissett GP, White BJ and Larson RL. Structured literature review of responses of cattle to viral and bacterial pathogens causing bovine respiratory disease complex. *J Vet Intern Med* 2015; 29: 770-780. DOI: 10.1111/jvim.12597.

12. Ellis WA. Leptospirosis as a cause of reproductive failure. *Vet Clin North Am Food Anim Pract* 1994; 10: 463-478. DOI: 10.1016/s0749-0720(15)30532-6.

13. Postma GC, Carfagnini JC and Minatel L. *Moraxella bovis* pathogenicity: an update. *Comp Immunol Microbiol Infect Dis* 2008; 31: 449-458. DOI: 10.1016/j.cimid.2008.04.001.

14. Geraghty T, Graham DA, Mullowney P, et al. A review of bovine Johne's disease control activities in 6 endemically infected countries. *Prev Vet Med* 2014; 116: 1-11. DOI: 10.1016/j.prevetmed.2014.06.003.

15. Humblet MF, Boschiroli ML and Saegerman C. Classification of worldwide bovine tuberculosis risk factors in cattle: a stratified approach. *Vet Res* 2009; 40: 50. DOI: 10.1051/vetres/2009033.

16. Holschbach CL and Peek SF. *Salmonella* in Dairy Cattle. *Vet Clin North Am Food Anim Pract* 2018; 34: 133-154. DOI: 10.1016/j.cvfa.2017.10.005.

17. Walker PJ and Klement E. Epidemiology and control of bovine ephemeral fever. *Vet Res* 2015; 46: 124. DOI: 10.1186/s13567-015-0262-4.

18. Muylkens B, Thiry J, Kirten P, et al. Bovine herpesvirus 1 infection and infectious bovine rhinotracheitis. *Vet Res* 2007; 38: 181-209. DOI: 10.1051/vetres:2006059.

19. Polat M, Takeshima SN and Aida Y. Epidemiology and genetic diversity of bovine leukemia virus. *Virol J* 2017; 14: 209. DOI: 10.1186/s12985-017-0876-4.

20. Fraser CM and Savan M. Bovine Papular Stomatitis. *Can Vet J* 1962; 3: 107-111.

21. Fray MD, Paton DJ and Alenius S. The effects of bovine viral diarrhoea virus on cattle reproduction in relation to disease control. *Anim Reprod Sci* 2000; 60-61: 615-627. DOI: 10.1016/s0378-4320(00)00082-8.

22. Clark MA. Bovine coronavirus. *Br Vet J* 1993; 149: 51-70. DOI: 10.1016/S0007-1935(05)80210-6.

23. Alexandersen S, Zhang Z, Donaldson AI, et al. The pathogenesis and diagnosis of foot-and-mouth disease. *J Comp Pathol* 2003; 129: 1-36. DOI: 10.1016/s0021-9975(03)00041-0.

24. Beard PM. Lumpy skin disease: a direct threat to Europe. *Vet Rec* 2016; 178: 557-558. DOI: 10.1136/vr.i2800.

25. O'Toole D and Li H. The pathology of malignant catarrhal fever, with an emphasis on ovine herpesvirus 2. *Vet Pathol* 2014; 51: 437-452. DOI: 10.1177/0300985813520435.

26. Snodgrass DR, Terzolo HR, Sherwood D, et al. Aetiology of diarrhoea in young calves. *Vet Rec* 1986; 119: 31-34. DOI: 10.1136/vr.119.2.31.

27. Letchworth GJ, Rodriguez LL and Del Cbarrera J. Vesicular stomatitis. *Vet J* 1999; 157: 239-260. DOI: 10.1053/tvjl.1998.0303.

28. Stuen S. *Anaplasma phagocytophilum* - the most widespread tick-borne infection in animals in Europe. *Vet Res Commun* 2007; 31 Suppl 1: 79-84. DOI: 10.1007/s11259-007-0071-y.

29. Bock R, Jackson L, de Vos A, et al. Babesiosis of cattle. *Parasitology* 2004; 129 Suppl: S247-269. DOI: 10.1017/s0031182004005190.

30. Thomson S, Hamilton CA, Hope JC, et al. Bovine cryptosporidiosis: impact, host-parasite interaction and control strategies. *Vet Res* 2017; 48: 42. DOI: 10.1186/s13567-017-0447-0.

31. Panuska C. Lungworms of ruminants. *Vet Clin North Am Food Anim Pract* 2006; 22: 583-593. DOI: 10.1016/j.cvfa.2006.06.002.

32. Sudhakara Reddy B, Sivajothi S and Rayulu VC. Clinical coccidiosis in adult cattle. *J Parasit Dis* 2015; 39: 557-559. DOI: 10.1007/s12639-013-0395-1.

33. Kaplan RM. *Fasciola hepatica*: a review of the economic impact in cattle and considerations for control. *Vet Ther* 2001; 2: 40-50.

34. Charlier J, Demeler J, Hoglund J, et al. *Ostertagia ostertagi* in first-season grazing cattle in Belgium, Germany and Sweden: general levels of infection and related management practices. *Vet Parasitol* 2010; 171: 91-98. DOI: 10.1016/j.vetpar.2010.03.006.

35. Lund A, Bratberg AM, Naess B, et al. Control of bovine ringworm by vaccination in Norway. *Vet Immunol Immunopathol* 2014; 158: 37-45. DOI: 10.1016/j.vetimm.2013.04.007.
