# Supplementary material for: Federation of European Laboratory Animal Science Associations recommendations of best practices for the health management of ruminants and pigs used for scientific and educational purposes
Source: Lab Anim. 2020 Aug 9;55(2):117–28. doi: 10.1177/0023677220944461 (PMC8044623; doi:10.1177/0023677220944461)
Supplement: sj-pdf-7-lan-10.1177_0023677220944461 - Supplemental material for Federation of European Laboratory Animal Science Associations recommendations of best practices for the health management of ruminants and pigs used for scientific and educational purposes [file sj-pdf-7-lan-10.1177_0023677220944461.pdf]

Appendix 7. Examples of agents for sheep

Infectious/ parasitic agent	Body system	Transmission route	Incubation period	Important clinical signs	Triggered by stress (Yes/ No)	Immunosuppressive / chronic	Important lesions at necropsy	Prophylactic disease control measures	Zoonosis (Yes/ No)	References
**Bacteria**										
Actinobacillus lignieresii	D, IN	Part of the normal flora of the respiratory and upper gastrointestinal tract. Penetration of wounds of skin, mouth, nose, gastro-intestinal tract, testicles and mammary gland	Variable	Diffuse abscesses and granuloma formation. Lip lesions, swollen lymph nodes and tongue protruding from the mouth, anorexia, excessive salivation	No	Occasionally chronic inflammation	Purulent discharges of white-green exudate containing small white-gray granules	Avoid poor quality, coarse feed	Possible but extremely un-common	1, 2
Brucella melitensis biovars 1-3	REP, D, I, L	Via contaminated feed and water, oral secretions, milk, urine, feces, semen, vaginal discharge (2-4 weeks), placental membranes. Entering through the mucuous membranes. Non-aborting females can give birth to persistently infected lambs	5-60 days. Infection at any time (including before gestation), but abortions usually late in gestation	Rarely: placentitis with abortion in the final trimester, followed by resistance; systemic disease with fever, depression, weight loss, diarrhea, mastitis, lameness, hygroma, orchitis, epididymitis	Yes, if overcrow-ding	No, cleared in 2-4 weeks	Rarely: mild placental lesions	Test and cull programmes. Veterinarians who encounter or suspect brucellosis should follow their national and/or local guidelines for disease reporting	Yes	3, 4
Brucella ovis	REP	Rams persistently infected, shedding this organism in semen for 2-4 years or longer (+/- clinical signs, intermittent), excretion in urine. Transmitted from ram to ram directly (through mucosae) or via ewes (mechanical vectors - do not become infected-carriers in vagina for 2 months). Ewes occasionally infected: shedding in vaginal discharges and milk (transient, rarely span more than one	Clinically detectable lesions became apparent 3-17 weeks after inoculation (experimentally infected rams). Changes in semen quality 1 week after infection	Epididymitis, orchitis and impaired fertility in rams. Poor quality semen with increased numbers of white blood cells, sperm motility and concentration decreased, and individual sperm abnormal. Palpable, permanent, painful unilateral (more common) or bilateral lesions in the epididymis and scrotum. Nodules or	No	Yes	Enlarged epididymis, thickened and fibrous, tunica vaginalis with extensive adhesions. Fibrous atrophy in the testis. Typical lesions in the placenta: yellow-grey plaques, coalescent in the intercotyledonary areas. Necrotic cotyledons. Gelatinous edema in the chorioallantois in severe cases. Mild	If encountered or suspect ed, national and/or local guidelines for disease reporting apply. Generally introduced into a flock through infected animals or semen. Flocks free of this organism not to be allowed to contact sheep that may be infected. Rams	No	1, 3, 5

		pregnancy). Lambs infected if nursing infected dams, congenitally infected lambs rare		testis atrophy in chronic cases. Abortions, stillbirths increased perinatal mortality, weak or small lambs. Systemic signs rare in adult ewes and rams			to moderate pneumonic changes in the fetus	not to be housed with rams of unknown status, or allowed to breed ewes that were recently mated by such rams		
Campylobacter fetus subsp. *intestinalis* and *Campylobacter jejuni*	REP, D	Commensals in the intestines and gallbladder. Infection: fecal-oral (fecal shedding - including in persistently infected animals that aborted), aborted fetuses, placenta, vaginal discharge. No venereal transmission. Contamination of the environment. Active organisms in uterine discharges for several months post abortion	3-60 days 7-12 days in ewes after experimental inoculation with *C. jejuni*	Abortion "storms" in the last trimester (70-90% of the flock), still births, weak lambs, agalaxia. Ewes' death in case of retention of the fetus *in utero*. Enzootic infections: <20% of the animals abort. Diarrhea, bacteriemia	No	Yes	Edematous aborted fetuses with serosanguinous fluids within the subcutis and muscle tissue fascia. Pale liver foci, thickened and edematous placental tissues, with serous fluids, gray placental cotyledons. "Gray target"-like necrotic areas on the livers of aborted lambs	Immediate isolation of aborting ewes from the rest of the flock, prompt decontamination of the area and disposal of the aborted tissues and discharges (incineration, kept away from guard dogs), avoidance of contamination of feedstuffs	Yes	1, 3, 6

Chlamydia abortus	REP, D, RES	Via ingestion and aerosols from placenta and uterine discharges at abortion or birth. Seldom more than 1 abort, but persistent infection and shedding in the ewe's reproductive tract for 2–3 days before and after ovulation. Other routes of transmission: fecal/urine- oral, direct inoculation into the eye and venereal transmission. After entering at the tonsils: latent intracellular bacteria.<br>Birds and foxes: vectors	Highly variable. Abortion soon after infection (if ewes infected more than 6 weeks before due lambing date). Nonpregnant animals: asymptomatic carriers, abort during next pregnancy or mount an effective immune response and never abort	Enzootic abortion of ewes: typically in the last 2–3 weeks of pregnancy: when introduced into naïve flocks, 25-60% abort. In flocks where epizootic: 1-15% abortions (new additions and primiparous). Transient fever and general malaise at initial infection, then asymptomatic until abortion. 48-72 hours before aborting: transient pneumonia and hepatitis, anorexia, fever, bloody vaginal discharge. Premature stillborn lambs and inflamed (retained) placentas/delivery of full-term stillborn lambs or weak lambs that do not survive > 48 hours. Clinically healthy female lambs born carriers of the bacteria =>abort at the first pregnancy	No	Yes, chronic: persistent subclinical infection in non-pregnant and multiparous ewes	Fetuses: rare gross lesions: ascites, lymphadenopathy, liver congestion. Histopathology in lambs: hepatitis, splenitis, broncho-pneumonia, encephalitis. Ewes: placentitis with necrotic, reddish brown cotyledons and thickened brown intercotyledonary areas covered by exudate	Prophylactic vaccination possible. Isolation of aborted ewes, removal and destruction of aborted material and infected bedding. Culling of ewes that aborted	Yes	3, 7-14
Chlamydia pecorum	D, IN, L, REP, RES	Present in nasal and ocular secretions, feces, urine of infected animals. Major transmission routes: fecal-oral and via inhalation	6-30 days	Clinical disease (polyarthritis) in 1-9 month-old lambs (3-5 month old most affected): fever, reluctant movements (joint stiffness +/- synovial effusion, lameness).Kerato-conjunctivitis, infertility, enteritis, reduced growth rates, mastitis, and	No	Yes	Fibrinous exudate in joints and oedema of surrounding tissue. Articular cartilage minimally affected	Isolation of diseased animals	No	3, 14-16

				pneumonia. Morbidity up to 80%, mortality 1%. Course of disease: 10-14 days: recovery, sometime residual lameness						
Clostridium perfringens type C	D, CV/HP, I, RES, N, L	Transmitted by ingestion of contaminated feed (ubiquitous in the environment, gastrointestinal tract and contaminated feeds)	1-3 days	Animals aged 3 days: hemorrhagic enterocolitis or found dead with no clinical presentation. Acute anemia, dehydration, anorexia, depression, tremors, convulsions, abdominal. Yearlings and adults: acute enterotoxaemia (“struck”): uneasiness, depression, convulsions	Yes	No	Milk-filled abomasum, hemorrhage in the distal small and large intestines. Petechial hemorrhages of the serosal surfaces (thymus, heart, gastrointestinal tract), hydropericardium, hydroperitoneum and hemorrhagic mesenteric lymph nodes. Pulmonary and brain edema	Vaccination	No	1, 17
Clostridium perfringens type D	D, CV/HP, RES, N, L, U	Normal inhabitant of the intestine, but if intestinal environment altered by sudden changes in diet or other factors => proliferation and production of a potent toxin acting locally or absorbed into the general circulation with devastating effects	Variable	Acute, subacute, or chronic neurologic condition: sudden death (1-2 hours) or neurologic and respiratory signs: blindness, opisthotonus, convulsions, bleating, frothing by the mouth and recumbency with paddling immediately before death	Yes	Yes	Similar to type C + “pulpy kidneys” (necrotic, soft), focal encephalomalacia, petechial hemorrhages on serosal surfaces of the brain, diaphragm, gastrointestinal tract and heart	Vaccination at 5 weeks old, appropriate feeding regimens for young and fast growing animals and feeding concentrates to adults	Yes	1, 17
Clostridium tetani	N, L	Soil contaminant, part of gut microbiota of herbivores. Introduced into the tissue through wounds and deep punctures, after banding castrations, tail docking, ear tagging	4 days -3 weeks	Sporadic, acute, fatal neuropathy. Bloat, muscular spasticity, prolapse of the third eyelid, rigidity and extension of the limbs leading to a	No	No	Nonspecific, except inflammatory reaction associated with the wound	Good sanitation, aseptic surgical procedures and vaccination	Yes	1

				stiff gait, inability to chew and hyperthermia. Retracted lips, drooling, hypersensitivity to external signs, “saw-horse” stance. Death in 3-10 days						
Corynebacterium pseudotuberculosis	IN, I, RES	Present in the pus from the abscesses. Entering through broken or intact skin or mucuous membranes, by inhalation or ingestion. Transmission through materials used in the management of the animals and biological vectors (flies). Environmental contamination due to a leaking abscess very high and persistent: survival in soil > 8 months, in bedding straw for 3 weeks, in hay for 2 months, in shearing stalls for 4 months	From 2 weeks (lymph node enlargement) to 2- 6 months (abscess formation)	Caseous lymphadenitis: caseous abscesses, enlargement of external lymph nodes (parotid, submandibular, supramammary, also prescapular and prefemoral) Enlargement of internal lymph nodes and major organ infection can lead to chronic weight loss, coughing, respiratory problems and to death	No	Yes	Caseous abscesses, with classic “onion ring” on cut surface	Best prevention: maintaining a disease-free flock. All new animals to be tested for the disease and examined for lymph node enlargement. Housing to be maintained free of objects that can cause skin injury, material used for management of the animals to be cleansed and disinfected after use. Control external parasites leading to pruritus -> scratching-> skin wounds	Yes	3, 18
Coxiella burnetii	REP	Transmission by aerosols or direct contact, ingestion. Infections persistent for several years and possibly lifelong. Shed in milk (4 months), feces (5 months) urine, vaginal secretions (4 months), semen, placenta and reproductive discharges during subsequent pregnancies	Incubation period variable (reproductive failure usually being the only sign of illness in naturally infected animals)	“Q Fever”. Asymptomatic in nonpregnant animals; placentitis and abortions in the third or second trimester or stillbirths. 5-50% of the flock affected. Abortions in successive parturitions	Yes, by stress, overcrow-ding, poor nutrition	Yes, chronic	Placenta with gross white areas of necrosis and mineralization of the cotyledons and intercotyle-donary area. Chorionic surface covered in thick exudate. Fetuses without gross lesions	Culling the animals that serve as permanent reservoirs. Prompt incineration of placentas	Yes	3, 19

		and lactations. Long term persistence in the environment, spread at long distances by the wind.<br>Vectors: ticks, lice, mites and parasitic flies								
Dichelobacter nodosus and *Fusobacterium necrophorum,* occasionally *Corynebacterium pyogenes*	IN, L	Source of *D. nodosus*: feet of infected animals that transfer the organisms to the soil (survival:7-14 days) and then to the feet of other animals. Persistence for 2-3 years in carrier animals. Wet environments predisposing to infection (leading to maceration of tissue and encouraging infection with *F. necrophorum* and, occasionally, C. *pyogenes)*	Variable	"Foot rot"<br>*F. necrophorum* => interdigital dermatitis - starting at 4-10 weeks old - necessary for infection with *D. nodosus* to occur. All ages susceptible, severity of disease increasing with age: severe lameness -> grazing on knees -> recumbency, fever, anorexia, weight loss in numerous animals. Usually affecting both claws in more than 1 foot	No	Yes	Benign strain of *D. nodosus* => soft horn underrun, without further pathological changes ("benign"/"nonprogressive footrot"): inflammation and necrosis of interdigital tissue – affects few animals. Virulent strain of *D. nodosus* => more severe disease ("virulent footrot"): the (entire) horn may separate from underlying tissue and express a malodorous exudate	Genetic selection for resistance to footrot. Separation of infected animals at foot trimming, grazing on unused pastures, foot baths, culling all severely affected animals	Yes (*C. pyogenes*)	3, 13
Leptospira interrogans serovars: *Hardjo, Pomona, Bratislava, Ballum, Icterohaemorrhagica, Grippotyphosa, Sejroe, Wolffi*	REP, U, D, CV/HP, N	Exposure to environments contaminated by urine from other species (e.g. wild rodents). Direct transmission rarely confirmed	2 to 12 weeks	Anorexia, fever, jaundice, hemoglobinuria, anemia, neurological signs, flaccid agalactia. Rarely: abortions in the last trimester of gestation	No	Yes	Swollen kidneys, pale or containing red or white/gray spots, mottling, bands of tissue or fibrotic scarring. Enlarged liver, sometimes with necrotic foci. Hemorrhages: petechiae or ecchymoses in various organs. Hematuria, icterus, abnormalities associated with acute uremia, pulmonary lesions or other lesions	Control of wild rodents	Yes	3, 20

Listeria monocytogenes	N, REP	Present in soil, water, plant litter, rotten wood, silage that is not properly acidified (if pH>5). Fecal shedding peaks in the winter. Infection through consumption of contaminated silage. Possibly invading the body through gastrointestinal tract	Variable	Meningoencephalitis, septicemia (in animals < 1 year old). Infection in late gestation: stillbirths, weak neonates rather than abortion, preceeded by septicemia (fever, decreased appetite, reduced milk production). The neurologic and the abortifacient form not seen simultaneously	No	Yes	Histopathological identification of microabscesses in the brain stem. Metritis post abortion or uterus filed with necrotic, dark colored, putrid material. Suppurative placentitis with necrotizing vasculitis. In chronically affected animals: thickened cotyledons with leathery texture. Severely autolyzed or macerated fetuses	Avoid feeding poor quality (insufficiently acidified) or spoiled silage or grazing on pastures linked to disease outbreaks, remove rotten wood from pastures	Yes	3, 7
Mycobacterium avium subsp. paratuberculosis	D, CV/HP	Bacterial shedding in feces and milk and transplacental transmission in animals showing clinical signs. Horizontal(fecal/colostrum/milk-oral) and vertical transmission from subclinical carriers. Organism very resistant in environment (1 year)	2-15 years	Chronic weight loss, chronic diarrhea (20% of cases), submandibular oedema because of low protein levels	Yes, also triggered by parturition	Yes	Thickening and corrugation of intestinal mucosa (distant jejunum and ileum). Thickened and cordlike lymphatic vessels, with enlargement of ileocecal and mesenteric lymph nodes, edema of abomasal wall, fluid accumulation in abdominal and pericardial cavities	Difficult prevention as difficult to diagnose on subclinical animals. Culling positive animals and offspring	Yes	3, 21
Mycoplasma agalactiae	IN, N, L, D, REP, RES	Shedding in nasal and ocular discharges, milk, also in: urine, feces and semen, presence in the external ear canal. Intermittent shedding in semen. Asymptomatic carriers for months to years, shedding in milk during > 1 lactation. Infection by ingestion, inhalation or through the teat opening. Milk and colostrum infectious.	1-8 weeks	Contagious agalactia: acute or chronic. Major clinical syndromes: mastitis, arthritis and keratoconjunctivitis. Initially, fever, nonspecific signs of illness. Clinical mastitis soon afterwards in lactating females. Hot and swollen	No	Yes	Catarrhal mastitis with primary inflammation of the interstitial tissues and enlargement of the mammary lymph nodes. Secondary acinar involvement, fibrosis and/ or parenchymatous atrophy of the udder. Periarticular edema - hemorrhagic or turbid joint fluid -	Once established in a herd: difficult to eliminate. Regular herd tests, with culling or isolation of infected animals and good management and hygiene reducing transmission	No	3, 22

		Aerosol transmission over short distances from animals with respiratory signs, and the Present in semen => possible venereal transmission. Via fomites (feed, drinking water and milking equipment)		udder, discolored or watery, granular or clotted milk. Arthritis or polyarthritis in tarsal and carpal joints, and becoming chronic. Short lasting ocular signs, chronic cases possible: blindness in one or both eyes. Diarrhea or respiratory signs: from coughing to dyspnea. Septicemia in nursing lambs. Occasional meningitis, abortions			cartilage unaffected. Serous or mucopurulent conjunctivitis or keratitis. Generalized peritonitis when death in acute stage. Vulvovaginitis, cystic catarrhal metritis and/or salpingitis, balanoposthitis or testicular degeneration	within the flock. The premises and equipment to be cleaned and disinfected regularly, sick animals to be isolated. Cleanliness and infection control measures especially important during milking. Separating young animals from milking animals to reduce their exposure		
Pasteurella hemolytica type A	RES	Common commensal of tonsils of young animals. Disease occurs when it gains access to lower respiratory tract	Variable	Enzootic pneumonia in young lambs and their mothers	Yes - the risk factors are: overcrow-ding, stress	No	Hemorrhagic bronchopneumonia, bluebag"/gangrenou s mastitis after being nursed by infected offspring	Avoiding stress factors	Yes	3, 7
Pasteurella multocida	RES	Disease if colonization of lower respiratory tract or if entering the blood stream. Direct spread between animals with nasal contact; indirect spread after contact with nasal secretions. Long persistence in the environment during warm, moist weather	Variable	Pneumonia and septicemia: bilateral purulent nasal discharge, coughing, diarrhea, anorexia, high fever. Septicemia in neonates and hemorrhagic septicemia in adults. Occasionally septic arthritis and mastitis ("bluebag"/gangrene of the udder)	Yes - risk factors: transport, overcrow-ding, changes to higher energy feeds, handling stress	No	Pneumonia lesions, with little hemorrhage and little fibrin (or without)	Avoiding stress factors	Yes	3
Salmonella enterica subsp. *enterica* serovar *abortus ovis*	REP	Mainly found in vaginal discharges, placenta, aborted fetuses, and infected newborns. Vaginal discharges highly infectious during the first week after an abortion	Animals infected at 1 month of gestation aborting after a 2–month incubation	Abortion storms in up tp 70% of the females, primarily during the second half or last third of gestation. Stillborn lambs or dying	Yes: triggered by climatic changes, shipping, overcrowding , food and/or water	Carrier state	Aborted fetus and placenta grossly normal or autolyzed. Signs of septicemia in the placenta: edema and hemorrhages in the	Maintenance of high health status closed flock or keeping purchased sheep segregated until lambing	Yes	3, 13

		and up to 1 month. Septicemic animals excreting it in feces, a few excreting the bacteria in the colostrum or milk. Respiratory secretions infectious in young lambs. Infection via oral, conjunctival, or respiratory routes. Venereal spread and asymptomatic carriers possible	period. If infection during the 3rd month of gestation: abortions after 20 days. No abortions if infections 1 month before mating	within a few hours of birth from septicemia ("cooked appearance"). Some lambs apparently healthy die within 3 weeks; in some: diarrhea or symptoms of pulmonary infections. Most ewes otherwise healthy; transient fever in some animals. Vaginal discharge for a few days before and after the abortion. Diarrhea is rare. Occasionally post–parturient metritis and peritonitis from secondary bacterial invaders	deprivation, inappropriate use of antibiotics		chorioallantois and necrosis or swelling of the cotyledons. Multifocal suppurative inflammation, necrosis, edema, or hemorrhages in the fetal tissues. Liver and spleen swollen, with pale foci. Young lambs or ewes with diarrhea: enteritis and abomasitis, swelling of the regional lymph nodes. Ewes dying with septicemia: acute metritis; uterus swollen and containing necrotic tissue, serous exudate, and a retained placenta			
Salmonella enterica subsp. *Enterica* serotypes *: arizonae, dublin, montevideo,* and *typhimurium (S.a., d.,m., t.)*	REP, D	Wild birds: vectors of *S.m*., cattle of *S.d.*, humans of *S.t.*, but also contaminated feedstuffs and water courses. Transmission by ingestion	Variable	Abortion and death of pregnant ewes. *S.t.* and *S.d:* Profuse dysentery, tooth grinding, pyrexia, congestion of mucuous membranes, no ruminal sounds, severe metritis after abortion*). S.m.*: Affected ewes: dull, depressed, isolated from flock, with fetid red-brown vaginal discharge	Yes: can be triggered by climatic changes, shipping, overcrowding, food and/or water deprivation, inappropriate use of antibiotics	Yes	*S.t.*: ewes found dead with autolytic/emphysematous lambs *in utero*, metritis or septic peritonitis	Isolation of all aborted sheep and separation of the group from other sheep for 6 weeks	Yes	3, 13
**Viruses**										
Bluetongue virus	IN, N, L, REP, RES	*Culicoides* spp. (insect host) transmitting the virus, after infection by feeding on viremic animals. Transmission	4-12 days	Pyrexia up to 42°C, excessive salivation, depression, panting; clear nasal discharge -> mucopurulent, crust around the	No	No	Congestion, oedema, hemorrhages and ulcerations of digestive and respiratory mucosae	Disease-free areas: vaccination, animal movement control,	No	3, 13

		through semen and blood also possible		nares. Hyperaemia and congestion of the muzzle, lips, face, eyelids and ears => oedema. Ulceration and necrosis of the mucosae of the mouth, hyperaemic and oedematous tongue; later cyanotic and protruding. Extension of hyperemia to coronary band of the hoof, the groin, axilla and perineum; lameness due to coronitis or pododermatitis and myositis. Torticolis in severe cases. Abortion or birth of malformed lambs. Pneumonia, emaciation. Either death within 8–10 days or long recovery with alopecia, sterility and growth delay			Severe bilateral broncholo-bular pneumonia (if complications); in fatal cases, lungs may show interalveolar hyperemia, severe alveolar oedema and bronchial tree filled with froth. Thoracic cavity and pericardial sac containing large quantities of plasma-like fluid; distinctive hemorrhages found at base of pulmonary artery. Congestion of hoof laminae and coronary band. Hypertrophy of lymph nodes and splenomegaly	quarantine and screening, vector control, especially in aircrafts. Infected areas: vector control, vaccination		
Border Disease Virus (BDV) and Bovine Viral Diarrhea Virus (BVDV)	IN, N, L, REP, I	Venereal transmission from shedding rams. Vertical transmission. Infection of fetuses and birth of persistently infected (PI) lambs - most potent source of infection: viraemic, antibody-negative and constantly excreting virus. Spreading from sheep to sheep. The commonest cause of BD = the pestivirus border disease virus (BDV); in some parts of the world, bovine viral diarrhea virus	4-11 days	Fetuses often infected early in gestation. Small dead fetuses resorbed or abortion unnoticed (asymptomatic ewes). As lambing approaches: abortion of larger fetuses, stillbirths and the premature births of small, weak lambs. Affected lambs: “hairy shaker disease”. Healthy	No	No	Lesions mainly microscopic: central nervous system and skin. Fetal infections at mid-gestation => cerebellar hypoplasia and dysplasia, hydronencephaly and porencephaly. Severe destructive lesions immune-mediated in lambs. Mucosal involvement in adults: erosions and	Identifying the viraemic PI animals and not using them for breeding or trading purposes. Serologically positive, nonviraemic sheep generally considered “safe” - latent infections not known to occur	No	3, 23

		(BVDV) = more common cause of BD. The source of BVDV for sheep: close contact with cattle		newborn and adult sheep exposed to BDV: mild or inapparent disease - slight fever and a mild leukopenia			ulcerations. Histologically: necro-suppurative inflammation	in recovered animals		
Contagious echtyma ("orf") virus	IN. RES, D	Direct contact with clinically affected animals or on fomites contaminated by the clinically affected. Indirect, by contact with virus-contaminated soil or shed scabs or from "mechanical" carriers. Typically entering through a break in the skin (e.g at tooth eruption). Persistence in soil for years	3-14 days	Papules->vesicules->pustules-> scabs (1-4 weeks until healing). Self-limiting, resolving in 3 weeks. Morbidity 100% in naïve flocks, mortality 1%. Death because of pneumonia or starvation (impossibility to feed because of severe oedema and necrosis in the mouth or of painful udder and teats lesions, preventing suckling). Sometimes gastroenteritis	Yes, by trans-portation stress	No	Characteristic macroscopic scab-like lesions on the lips, muzzle, in the oral cavity. Crusty proliferations at mucocutaneous junctions. Lesions on ears, face, periorbital region, scrotum, perianal region, extremities. Histopathology: ballooning and degeneration of keratinocytes, eosinophilic intracytoplasmatic viral inclusions	Isolation of affected stock, prevention of the disease entering the farm through quarantine, thorough clinical examination of new additions and purchasing new stock from ecthyma-free farms	Yes	3, 7
Ovine Pulmonary Adenocarcinoma Virus ("Jaagsiekte", pulmonary adenomatosis)	RES, I	Respiratory route: via aerosols or droplets. Present in respiratory exudates, tumors, lung fluids, peripheral blood leucocytes and lymphoid organs; before tumors develop, the virus is detected in lymphoreticular cells. Horizontal transmission among sheep of all ages, neonates particularly susceptible. Spread in milk or colostrum. Short survival in the environment. Transmission is facilitated	6 months to 3 years. It appears to be age-dependent, and is longer in older sheep	Clinical signs only in animals that have developed tumors: progressive emaciation, weight loss and respiratory compromise, particularly after exercise (lag behind the flock). Thin mucus discharge from the nostrils; if the head is lowered => copious frothy exudate pouring from nares. Moist rales at auscultation, coughing not prominent. Slowly	No	Yes	Lungs enlarged in animals with tumors; in advanced cases: not collapsing upon opening the chest cavity. Frothy fluid in the trachea and bronchi. Tumors in the lungs: small nodules to solid masses, sharply demarcated, firm, and gray or pinkish–gray. On cut surface: glistening and granular tumors; expressing frothy fluid. Secondary pneumonia and	No diagnostic test to detect asymptomatic carriers => difficult prevention of entry. Herd replacements to be bought only from flocks with no history of ovine pulmonary adenomatosis (not an absolute guarantee, because of long incubation period)	No	24, 25

		by close confinement (e.g. indoor housing)		progressive clinical signs, ending in severe dyspnea. Death in days to months, from secondary bacterial pneumonia. Most cases at >2 years old; peak incidence in 3- to 4-year-old animals. Rare in sheep < 7-9 months of age. Appearance of tumors => death. High morbidity and mortality rates (80%) in recently infected flocks			fibrinous pleuritis. Tumor metastasis to nearby lymph nodes	Removing affected sheep and the offspring of infected ewes. Embryo transfer to save the genetic potential from flocks that are euthanized. Significant reduction by removing lambs at birth and hand-rearing them with colostrum substitutes or cow colostrum, followed by milk replacer. Good disinfection and general hygiene		
Rotavirus group A-C	D	Mainly by fecal-oral route. Waterborne or airborne (respiratory) routes possible	Up to 48 hours	Acute diarrhea in animals aged 2-14 days, depression and dehydration	Yes	No	Intestinal villus atrophy	Immunization of dams. Giving colostrum supplements in milk during the period of risk. The management of pregnant animals at the time of parturition ensuring the minimum exposure of newborn animals to infectious agents	Yes – group A	3, 26, 27
Small ruminant Lentiviruses: Maedi Visna Virus	RES, N, L, I	Infection early in life, from drinking infected colostrum or milk. Through close contact	The incubation period for “maedi” > 2 years; clinical	Mainly subclinical, rare progressive, untreatable disease	No	Chronic, lifelong persistence of the causal agent in host	Progressive interstitial pneumonia, progressive lesions	Additions to uninfected herds from negative herds.	No	3, 28

		(through respiratory secretions) or by fecal-oral route. Rare indirect spread (from water contaminated with feces), intrauterine spread negligible or minor. Infection for life, but variable viral burdens between individual animals. Both asymptomatic and symptomatic carriers	signs typically develop at 3-4 years old. Shorter incubation period for “visna”: symptoms can appear in sheep >2 years old. Death within 1 year after development of clinical signs	syndromes: dyspnea, progressive interstitial pneumonia and emaciation caused by ovine progressive pneumonia (OPP) (“maedi”) or neurologic signs and paralysing meningoencephalitis (“visna”). Both maedi and visna eventually fatal. Indurative mastitis in clinically affected animals		monocytes and macrophages	of joints, udder and central nervous system. The lungs not collapsing when removed from the thorax and often retaining the impression of the ribs. The lungs and lymph nodes increasing in weight (up to 2–3 times)	New additions to be quarantined and tested within 60 days of arrival. A programme of periodic testing and culling of all seropositive animals to eradicate the virus from a flock. Lambs to be separated from older animals and fed heat-treated colostrum		
Schmallenberg Virus	REP	Transmitted vertically and via insect vectors: *Culicoides obsoletus* and *C.dewulfi*	1-4 days	Abortions, congenital malformation in newborns	No	No	Malformations in fetuses: arthrogryposis, torticollis, scoliosis and kyphosis, brachygnatia inferior, and mild-to-marked hypoplasia of the cerebrum, cerebellum and spinal cord	Control of potential vectors during the vector-active season to decrease transmission. Delay of breeding to decrease the number of fetal malformations	No	29, 30
**Parasites**										
Haemonchus contortus	D, CV/HP	Fecal-oral. Larvae hatching shortly after eggs passing in the feces and reaching the infective stage in 2 weeks under optimal temperatures (24°C). Development to the infective stage delayed during cold weather	In the hyperacute disease, death within 1 week of heavy infestation without significant signs	Hyperacute, acute, or chronic. Acute form: severe anemia, submandibular, then generalized edema, tachypnea and tachycardia. Chronic form: anemia, increased gastric => abomasal dysfunction. Mature sheep: heavy, even fatal, infections,	No	Yes	Large number of adult parasites seen and identified on the surface of the abomasum, and small petechiae visible where the worms have been feeding	Sustainable parasite control programmes for individual farms/user establishments based on the unique circumstance and management	No	13

				particularly during lactation						
Cryptosporidium parvum	D, RES	Fecal-oral route, directly or on fomites: contaminated food and water. Shed in feces of symptomatic and asymptomatic individuals (shedding oocysts in stressful periods – e.g. around parturition). Sporulated oocyts shed in the feces immediately infectious. Autoinfection causing severe disease	In young animals, clinical signs appearing after 3-5 days	Lambs aged 3-7 days old most commonly affected. Clinical cases: mild to severe watery diarrhea, yellow or pale brown, containing mucus. Anorexia, lethargy and weight loss, respiratory signs. Most clinical cases self-limiting within 1-2 weeks, but dehydration, debilitation and deaths are possible	Yes	Yes	Not specific: increased fluidity of the intestinal contents, hyperemia of the intestinal mucosa, and distension of the large and/ or small intestine. Enlarged mesenteric lymph nodes. Mild to severe villous atrophy in the intestines, and spherical organisms in the brush border	Low infective dose, making control difficult. Sanitation (e.g., regular cleaning of pens) and manure management to reduce the level of exposure to oocysts. Steam cleaning and disinfection, or thorough cleaning with hot water, followed by drying to promote desiccation. Good nutrition and management practices to avoid stress. Sick animals to be isolated	Yes	13, 31
Dicrocoelium dendriticum	D	Fecal-oral: parasite eggs eliminated in the feces of sheep and ingested by terrestrial snails. Cercaria develop in the snails, are secreted in mucus, and are ingested by ants. The definitive host then ingests the ants while grazing. The metacercaria migrate up the common bile duct	Depending on the level of challenge: immediate acute disease, subacute disease after weeks, chronic disease apparent several months later	Anemia, ill thrift	Yes	Yes	Scarring of the liver, and histologic lesions typical of perilobular cirrhosis	Difficult to prevent	No	1, 2
Dictyocaulus filaria, « lungworm »	RES	Adult *D. filaria* in the trachea and bronchi lay eggs with fully developed L1. L1 hatch quickly, are coughed up, swallowed and passed in feces. In 5 days, L3 migrate to grass	21-28 days	Normally not clinically significant. Clinically apparent if first challenge or high challenge: harsh coughing,	No	Chronic, causes ill thrift	If large numbers of larvae are ingested: death - severe interstitial emphysema and Other lesions:	Prophylactic anthelmintic treatment according to the herd health plan	No	2, 32

		tips and are ingested, penetrate the intestinal mucosa, moult to L4 in the mesenteric lymph nodes, then migrate via lymphatics and blood to the caudal lobes of the lungs		dyspnea, rectal prolapse after severe coughing paroxysms. Mild to moderate pneumonia with coughing in lambs. Deaths uncommon (if secondary pasteurellosis)			parasitic pneumonia (ventral areas of the Caudal lung lobes), severe bronchiolitis and bronchitis. Adult worms 30 to 100 mm long in the trachea and bronchi. pulmonary edema			
Fasciola hepatica	D, CV/HP	For animals on pastures: late spring /early summer infestation of snails by miracidia resulting in an autumn metacercariae challenge to sheep	Depending on the level of challenge: immediate acute disease, subacute disease after weeks, chronic disease apparent several months later	Acute form: sudden death by hemorrhage and liver damage, other animals lethargic, with pale mucuous membranes and with reduced grazing activity. Subacute form: rapid weight loss => very poor body condition score and poor fleece quality, marked anemia, severe depression, inappetence, weakness, unable to stand, fetal death/resorption	Yes	Yes	Acute form: liver enlargement, ascites/peritoneal exudate. Subacute form: liver enlargement, ascites/peritoneal exudate, visible only by ultrasound examination/ necropsy. Chronic form: low body condition score and poor fleece quality, submandibular oedema, anemia, death in advanced gestation/early lactation	Measures to reduce the metacercariae, challenge in autumn, strategic drenching, fencing off snails' habitats (expensive measure)	Yes	13, 33
Neospora caninum	REP, N	3 life cycles: tachyzoites, tissue cysts (both found in intermediate hosts: sheep) and oocysts (found in definitive hosts: dogs). Oocysts sporulation outside the host. Fecal-oral transmission (sporulated oocysts can also contaminate food and water). Transplacental transmission of tachyzoites for several generations	Not described. Reactivation of latent infection possible	Abortions, near-term, mummified stillborn fetuses	Yes	Yes	Autolyzed fetuses and placentas or placentitis with necrosis and mineralization of cotyledonary vili, mild multifocal necrosis in the brain	Food to be stored in facilities preventing contamination by dogs' feces	No	3, 34-36

Toxoplasma gondii	REP, N	Sporulated oocysts surviving for long periods under moderate environmental conditions – months to years in soil. Infective oocysts ingested with food or water, inhaled in aerosols. Transplacental transmission possible (5%)	5-23 days. Reactivation possible several years after infection	Most abortions during the latter half of gestation. Infection during early pregnancy (up to 40 days): embryo/early fetal loss => irregular and extended intervals of return to oestrus. Infection during mid-pregnancy (40-120 days): abortion/mummification. Infection after 120 days of gestation: premature, stillborn or weak lambs. Affected does clinically normal (exception: immunosuppressed pregnant females: febrile and develop the neurological form)	Yes	No	Mummified fetuses: dark brown leathery appearance. 1-3 mm gray-white necrotic foci on the dark-red cotyledons of the placenta. The intercotyledonary region normal or slightly edematous	Food to be stored in facilities preventing contamination by cats' feces or vermin, maintenance of a healthy adult cat population by appropriate vaccinate and neutering. Cats not to be allowed near pregnant sheep. Vaccination possible	Yes	3, 7, 13, 36

Body systems: CV/ HP: Cardiovascular and hematopoietic system, D: Digestive system, I: Immune system, IN: Integumentary system; skin, hoof and claw, N: Nervous system, L: Locomotor system, REP: Reproductive system, RES: Respiratory system, U: Urinary system

References

1. Fox J, Otto G, Pritchett-Corning K, et al. *Laboratory Animal Medicine*. 3rd ed.: Academic Press 2015.

2. Sargison N. *Sheep Flock Health: A Planned Approach*. 1st ed.: Wiley-Blackwell, 2008.

3. Pugh DG, Baird N, *Sheep and Goat Medicine*. 2nd ed.: Elsevier, 2011.

4. Spickler AR. Brucellosis: *Brucella melitensis*, http://www.cfsph.iastate.edu/DiseaseInfo/factsheets.php (2018, accessed 30th June 2019).

5. Spickler AR. Ovine Epididymitis: *Brucella ovis*, http://www.cfsph.iastate.edu/DiseaseInfo/factsheets.php (2018, accessed 30th June 2019).

6. Spickler AR. Zoonotic Campylobacteriosis, http://www.cfsph.iastate.edu/DiseaseInfo/factsheets.php (2013, accessed 6th June 2019).

7. Allen MK and Borkowski GL. *The Laboratory Small Ruminant*. 1st ed.: CRC Press, 1999.

8. Borel N, Doherr MG, Vretou E, et al. Seroprevalences for ovine enzootic abortion in Switzerland. *Prev Vet Med* 2004; 65: 205-216. 2004/10/19. DOI: 10.1016/j.prevetmed.2004.08.005.

9. Buxton D, Anderson IE, Longbottom D, et al. Ovine chlamydial abortion: characterization of the inflammatory immune response in placental tissues. *J Comp Pathol* 2002; 127: 133-141. 2002/10/02. DOI: 10.1053/jcpa.2002.0573.

10. Essig A and Longbottom D. *Chlamydia abortus*: New aspects of infectious abortion in sheep and potential risk for pregnant women. *Curr Clin Microbiol Rep* 2015; 2: 22-34.

11. Longbottom D and Coulter LJ. Animal chlamydioses and zoonotic implications. *J Comp Pathol* 2003; 128: 217-244. 2003/07/02. DOI: 10.1053/jcpa.2002.0629.

12. Longbottom D, Livingstone M, Maley S, et al. Intranasal infection with *Chlamydia abortus* induces dose-dependent latency and abortion in sheep. *PLoS One* 2013; 8: e57950. 2013/03/08. DOI: 10.1371/journal.pone.0057950.

13. Scott PR. *Sheep Medicine*. 2nd ed.: CRC Press, 2015.

14. Spickler AR. Zoonotic Chlamydiae Maintained in Mammals, http://www.cfsph.iastate.edu/DiseaseInfo/factsheets.php (2017, accessed 6th June 2019).

15. Walker E, Jelocnik M, Bommana S, et al. Understanding the health and production impacts of endemic *Chlamydia pecorum* infections in lambs. *Vet Microbiol* 2018; 217: 90-96. 2018/04/05. DOI: 10.1016/j.vetmic.2018.03.009.

16. Walker E, Moore C, Shearer P, et al. Clinical, diagnostic and pathologic features of presumptive cases of *Chlamydia pecorum*-associated arthritis in Australian sheep flocks. *BMC Vet Res* 2016; 12: 193. 2016/09/10. DOI: 10.1186/s12917-016-0832-3.

17. Uzal FA and Songer JG. Diagnosis of *Clostridium perfringens* intestinal infections in sheep and goats. *J Vet Diagn Invest* 2008; 20: 253-265. 2008/05/08. DOI: 10.1177/104063870802000301.

18. Guimaraes AS, Carmo FB, Heinemann MB, et al. High sero-prevalence of caseous lymphadenitis identified in slaughterhouse samples as a consequence of deficiencies in sheep farm management in the state of Minas Gerais, Brazil. *BMC Vet Res* 2011; 7: 68. 2011/11/10. DOI: 10.1186/1746-6148-7-68.

19. Spickler AR. Q-Fever, http://www.cfsph.iastate.edu/DiseaseInfo/factsheets.php (2017, accessed 6th June 2019).

20. Spickler AR. Leptospirosis http://www.cfsph.iastate.edu/DiseaseInfo/factsheets.php (2013, accessed 6th June 2019).

21. Spickler AR. Paratuberculosis http://www.cfsph.iastate.edu/DiseaseInfo/factsheets.php (2017, accessed 6th June 2019).

22. Spickler AR. Contagious Agalactia, http://www.cfsph.iastate.edu/DiseaseInfo/factsheets.php (2018, accessed 6th June 2019).

23. Border disease, https://www.cabi.org/isc/datasheet/91616 (accessed 6th June 2019).

24. Scott P, Griffiths D and Cousens C. Diagnosis and control of ovine pulmonary adenocarcinoma (Jaagsiekte) *In Practice* 2013; 35: 382-393.

25. Spickler AR. Ovine Pulmonary Adenocarcinoma, http://www.cfsph.iastate.edu/DiseaseInfo/factsheets.php (2019, accessed 6th June 2019).

26. Rotavirus infections in livestock and poultry, https://www.cabi.org/isc/datasheet/66267 (accessed 6th June 2019).

27. Papp H, Malik YS, Farkas SL, et al. Rotavirus strains in neglected animal species including lambs, goats and camelids. *Virus disease* 2014; 25: 215-222. 2015/02/13. DOI: 10.1007/s13337-014-0203-2.

28. Spickler AR. Small Ruminant Lentiviruses: Maedi-Visna & Caprine Arthritis and Encephalitis, http://www.cfsph.iastate.edu/DiseaseInfo/factsheets.php (2015, accessed 6th June 2019).

29. Van den Brom R, Luttikholt SJ, Lievaart-Peterson K, et al. Epizootic of ovine congenital malformations associated with Schmallenberg virus infection. *Tijdschr Diergeneeskd* 2012; 137: 106-111. 2012/03/08.

30. Wernike K, Elbers A and Beer M. Schmallenberg virus infection. *Rev Sci Tech* 2015; 34: 363-373. 2015/11/26. DOI: 10.20506/rst.34.2.2363.

31. Spickler AR. Cryptosporidiosis, http://www.cfsph.iastate.edu/DiseaseInfo/factsheets.php (2018, accessed 6th June 2019).

32. Ballweber LR. Overview of Lungworm Infection https://www.msdvetmanual.com/respiratory-system/lungworm-infection/overview-of-lungworm-infection (2014, accessed 6th June 2019 2019).

33. Ballweber LR. *Fasciola hepatica* in Ruminants https://www.msdvetmanual.com/digestive-system/fluke-infections-in-ruminants/fasciola-hepatica-in-ruminants (2014, accessed 6th June 2019).

34. Dubey JP and Lindsay DS. *Neospora caninum* induced abortion in sheep. *J Vet Diagn Invest* 1990; 2: 230-233. 1990/07/01. DOI: 10.1177/104063879000200316.

35. Dubey JP and Lindsay DS. A review of *Neospora caninum* and neosporosis. *Vet Parasitol* 1996; 67: 1-59. 1996/12/02. DOI: 10.1016/s0304-4017(96)01035-7.

36. Dubey JP and Lindsay DS. Neosporosis, toxoplasmosis, and sarcocystosis in ruminants. *Vet Clin North Am Food Anim Pract* 2006; 22: 645-671. 2006/10/31. DOI: 10.1016/j.cvfa.2006.08.001.
