# Supplementary material for: Federation of European Laboratory Animal Science Associations recommendations of best practices for the health management of ruminants and pigs used for scientific and educational purposes
Source: Lab Anim. 2020 Aug 9;55(2):117–28. doi: 10.1177/0023677220944461 (PMC8044623; doi:10.1177/0023677220944461)
Supplement: sj-pdf-8-lan-10.1177_0023677220944461 - Supplemental material for Federation of European Laboratory Animal Science Associations recommendations of best practices for the health management of ruminants and pigs used for scientific and educational purposes [file sj-pdf-8-lan-10.1177_0023677220944461.pdf]

Appendix 8. Examples of agents for goats

Infectious/ parasitic agent	Body system	Transmission route	Incubation period	Important clinical signs	Triggered by stress (Yes/ No)	Immuno-suppressive/ chronic	Important lesions at necropsy	Prophylactic disease control measures	Zoonosis (Yes/ No)	References
**Bacteria**										
Brucella melitensis biovars 1-3	REP, N, L	Via contaminated feed and water, oral secretions, milk, urine, feces, semen, vaginal discharge (2-3 months), placental membranes. Entering the mucuous membranes and localizing in the udder, uterus, testes, spleen, lymph nodes, placenta. Non-aborting females giving birth to persistently infected kids	5-60 days Animals infected at any time (including before gestation), abortions usually late in gestation	Placentitis with abortion in the final trimester, followed by a period of resistance. Systemic disease with fever, depression, weight loss, diarrhea, mastitis, lameness, hygroma, orchitis, epididymitis	Yes, if overcrow-ding	Yes	Mild placental lesions	Test and cull programmes	Yes	1, 2
Campylobacter fetus subsp. *intestinalis* and *Campylobacter jejuni*	REP, D	Fecal-oral: normally inhabit the gastrointestinal tract shed in the feces of the persistently infected animals that have already aborted. No venereal transmission. Contamination of the environment due to shedding in placenta, fetuses, uterine fluids. Active organisms in uterine discharges for several months post abortion	3-60 days	Abortion "storms" in the last trimester, still births, weak kids, agalaxia. Aborting does usually asymptomatic or having diarrhea and mucopurulent vaginal discharge. Aborted fetuses and placentas expelled with little or no autolysis. Abortion storms: 70-90% of the animals; enzootic infections: <20% of the animals abort	No	Yes	Oedematous aborted fetuses with serosanguinous fluids within the subcutis and muscle tissue fascia. Pale liver foci, thickened and edematous placental tissues, with serous fluids, gray placental cotyledons. "Gray target"-like necrotic areas on the livers of aborted lambs	Immediate isolation of aborting ewes from the rest of the flock, prompt decontamination of the area and disposal of the aborted tissues and discharges (incineration, keeping away from guard dogs), avoiding contamination of feedstuffs	Yes	1, 3, 4
Chlamydia abortus	REP, RES, D	Via ingestion and aerosols from placenta and uterine discharges at abortion or birth. Shed in feces urine, milk	Sometimes abortion soon after infection (infection occurring >6 weeks before the due lambing date). Abortions from	Enzootic abortion of does: typically in the last 2–3 weeks of pregnancy. Abortion of 25-60% of does at introduction into naïve flocks. In flocks where the disease is epizootic: abortion rates 1-15% (new additions and	No	Yes, chronic: persistent subclinical infection in non-pregnant and multiparous does	Rare gross lesions in the fetus: ascites, pneumonia, lymphadenopathy, hepatitis. Kids: white spots on the liver. Does: placentitis with necrotic, reddish brown cotyledons	Prophylactic vaccination possible. Isolation of aborted does, removal and destruction of aborted material and infected bedding.	Yes	1, 3-7

			nonpregnant subclini-cal carriers during the next gestation	primiparous). In some animals: persistent cough, polyarthritis or keratoconjunctivitis			and thickened brown intercotyledonary areas	Potential culling of does that aborted		
Clostridium perfringens type D	D, CV/HP, RES, N, L, U	Normal inhabitant of the intestine; if intestinal environment altered by sudden changes in diet or other factors => proliferation and production of a potent toxin acting locally or absorbtion into the general circulation with devastating effects	Depending on form	Acute, subacute, or chronic neurologic condition: sudden death (1-2 hours) or neurologic and respiratory signs, blindness, opisthotonos, convulsions, bleating, frothing by the mouth and recumbency with paddling immediately before death	Yes	Yes	"Pulpy kidneys" (necrotic, soft), focal encephalomalacia, petechial hemorrhages on serosal surfaces of the brain, diaphragm, gastrointestinal tract and heart	Vaccination at 5 weeks old, appropriate feeding regimens for young and fast growing animals and feeding concentrates to adults	Yes	3, 8
Clostridium tetani	N, L	Soil contaminant, part of gut microbiota of herbivores. Introduced into the tissue through wounds and deep punctures, after banding castrations, tail docking, ear tagging	4 days-3 weeks	Sporadic, acute, fatal neuropathy. Bloat, muscular spasticity, prolapse of the third eyelid, rigidity and extension of the limbs leading to a stiff gait, inability to chew and hyperthermia. Retracted lips, drooling, hypersensitivity to external signs, "saw-horse" stance	No	No	Nonspecific, except inflammatory reaction associated with the wound	Good sanitation, aseptic surgical procedures and vaccination	Yes	3, 5
Corynebacterium pseudotuberculosis	IN, RES, I	Environmental contamination from a leaking abscess very high and persistent. Entering the body through broken or intact skin or mucuous membranes, by inhalation or ingestion. Via materials used in the management of the animals and biological vectors (flies). Survival in soil more than 8 months, in bedding straw: for 3 weeks, in hay for 2 months, in shearing stalls: 4 months	From 2 weeks (lymph node enlargement) to 2- 6 months (abscess formation)	Caseous lymphadenitis: caseous abscesses, enlargement of external lymph nodes (parotid, submandibular, supramammary, also prescapular and prefemoral). Enlargement of internal lymph nodes and major organ infection => chronic weight loss, coughing, respiratory problems and death	No	Yes	Caseous abscesses	Best prevention by maintaining a disease-free herd. Test of all new animals for the disease and examination for lymph node enlargement. Housing maintained free of objects causing skin injury, material used for management of the animals to	Yes	1, 5

								be cleansed and disinfected after use. Control of external parasites leading to pruritus -> scratching-> skin wounds		
Coxiella burnetii	REP	Via aerosols, ingestion or direct contact. Persistent infections for several years, possibly lifelong. Localization in the mammary glands, supramammary lymph nodes, uterus, placenta and fetus in animals; shedding in milk (4 months), feces (5 months) urine, vaginal secretions (4 months), semen, placenta and reproductive discharges during subsequent pregnancies and lactations. Long persistence in the environment; spread at long distances by the wind	28-56 days (experimental infection)	"Q Fever". Asymptomatic in nonpregnant animals; placentitis and abortions in the third or second trimester or stillbirths. Anorexia and depression 1-2 days before aborting. Abortions in successive parturitions possible	Yes, by stress, overcrow-ding, poor nutrition	Yes, chronic	The placenta with gross, white areas of necrosis; mineralization of the cotyledons and intercotyledonary area. Chorionic surface covered in thick exudate. Fetuses with no gross lesions	Culling the animals that serve as permanent reservoirs. Prompt incineration of placentas	Yes	1, 5, 9, 10
Dichelobacter nodosus and *Fusobacterium necrophorum*, occasionally *Corynebacterium pyogenes*	IN, N	*D. nodosus*: transmitted by the feet of infected animals to the soil (survival: days- weeks) and then to the feet of other animals. Persistance for years in carrier animals. Wet environments predisposing to infection and leading to maceration of tissue and encouraging infection with *F. necrophorum* and, occasionally, C. *pyogenes*	Variable	Footrot. Interdigital dermatitis (and severe lameness) caused by *F. necrophorum* , necessary for infection with *D. nodosus* to occur. All ages susceptible, severity of disease increasing with age: severe lameness -> grazing on knees -> recumbency, fever, anorexia, weight loss in numerous animals. Usually both claws affected in more than 1 foot	No	Yes	Benign strain of *D. nodosus* =>soft horn underrun, without further pathological changes ("benign"/ "non-progressive footrot": inflammation and necrosis of interdigital tissue – affects few animals). Virulent strain of *D. nodosus* => more severe disease ("virulent footrot": the (entire) horn separated from underlying tissue, malodorous exudate)	Genetic selection for resistance to footrot. Separation of infected animals at foot trimming, grazing on unused pastures, foot baths with 15% zinc sulfate solution, culling of all severely affected animals	Yes (*C. pyogenes*)	1, 5
Leptospira interrogans serovars: *Hardjo, Pomona, Bratislava, Ballum, Icterohaemorrhagica,*	REP, U, D, CV/HP, N	Exposure to environments contaminated by urine from other species (e.g. wild rodents). Direct transmission rarely confirmed	Variable, depending on the evolution of the disease: from days to weeks	Anorexia, fever, jaundice, hemoglobinuria, anemia, neurological signs, flaccid agalactia, (fatal) abortions in the	No	Yes	Petechial hemorrhage on surface of serosal membranes, enlarged kidney, autolyzed fetuses	Control of wild rodents	Yes	1, 11, 12

Grippotyphosa, Sejroe, Wolffi				last trimester of gestation						
Listeria monocytogenes	N, REP	Present in soil, water, plant litter, silage. Peak of fecal shedding in winter. Infection through consumption of contaminated silage. Transmission via milk => fatal septicemia	Abortion after 9-11 days in experimentally infected does	Meningoencephalitis, septicemia or, if infection in late gestation => stillbirths, weak neonates rather than abortion, preceeded by septicemia (fever, decreased appetite, reduced milk production). The neurologic and the abortifacient form seen simultaneously	No	Yes	Occasionally metritis post abortion or uterus filed with necrotic, dark colored, putrid material. Suppurative placentitis with necrotizing vasculitis. In chronically affected animals: thickened cotyledons with leathery texture. Severely autolyzed or macerated fetuses	Avoiding feeding poor quality or spoiled silage or grazing on pastures linked to disease outbreaks	Yes	1, 5
Mycobacterium avium subsp. *para-tuberculosis*	D, ICV/HP	Bacterial shedding in feces and milk and transplacental transmission more common in animals with clinical signs. Transmission by subclinical carriers via horizontal (fecal/colostrum/milk-oral) and vertical routes. Organism very resistant in environment (1 year survival)	2-15 years until appearance of clinical signs	Chronic weight loss, chronic diarrhea (20% of cases), submandibular oedema because of low protein levels	Yes, also triggered by parturition	Yes	Thickening and corrugation of intestinal mucosa (especially in distant jejunum and ileum). Thickened and cordlike lymphatic vessels, with enlargement of ileocecal and mesenteric lymph nodes, edema of abomasal wall, fluid accumulation in abdominal and pericardial cavities	Difficult prevention as difficult to diagnose on subclinical animals. Culling positive animals and offspring	Yes	1, 13
Mycoplasma agalactiae	IN, N, L, D, REP, RES	Shedding in nasal and ocular discharges and milk, also in urine, feces and semen, in the external ear canal. Intermittent shedding in semen. Asymptomatic carriers for months to years, shedding in milk during > 1 lactation. Infection by ingestion, inhalation or through the teat opening. Milk and colostrum infectious. Aerosol transmission over short distances from animals with respiratory signs, and the Present in semen => possible venereal transmission. Via fomites (feed, drinking water and milking equipment)	1-8 weeks	Contagious agalactia: acute or chronic illness. Mastitis, arthritis and keratoconjunctivitis. Initially: fever and nonspecific signs of illness, followed by clinically apparent mastitis in lactating females. Hot and swollen udder, milk usually discolored, often with a yellowish	No	Yes	Catarrhal mastitis with primary inflammation of the interstitial tissues and enlargement of the mammary lymph nodes. Secondary acinar involvement, fibrosis and/ or parenchymatous atrophy of the udder. Periarticular	Once established in a herd, difficult to eliminate. Regular herd tests, with culling or isolation of infected animals and good management and hygiene to reduce	No	1, 14

				tinge or watery, granular or clotted. Arthritis or polyarthritis most often in the tarsal and carpal joints, potential to become chronic. Ocular signs only for a short time, chronic cases possible: blindness in one or both eyes. Diarrhea or respiratory signs: from coughing to dyspnea. Septicemia most often in nursing kids. Rarely: neurological signs (meningitis), abortion			edema around the affected joints in animals - hemorrhagic or turbid joint fluid - cartilage is unaffected. Serous or mucopurulent conjunctivitis or keratitis. Generalized peritonitis if death during the acute stage. Vulvovaginitis, cystic catarrhal metritis and/or salpingitis, balanoposthitis or testicular degeneration	transmission within the herd. Regular cleaning and disinfection of the premises and equipment, isolation of sick animals. Cleanliness and infection control measures especially important during milking. Separation of young animals from milking animals		
Pasteurella multocida	RES	Disease if colonization of the lower respiratory tract or if entering the blood stream. Direct spread between animals with nasal contact. Indirect spread after contact with nasal secretions. Long persistence in the environment during warm, moist weather	Variable	Pneumonia and septicemia: bilateral purulent nasal discharge, coughing, diarrhea, anorexia, high fever. Septicemia in neonates and haemorrhagic septicemia in adults. Occasionally septic arthritis and mastitis (“bluebag”/gangrene of the udder)	Yes - risk factors: transport, overcrowding, changes to higher energy feeds, handling stress	No	Pneumonia lesions, with little hemorrhage and little fibrin (or without)	Avoiding stress factors.	Yes	1, 5
Salmonella enterica subsp. *enterica* serotypes*: arizonae, dublin, montevideo,* and *typhimurium (S.a., d.,m., t.)*	REP, D	Wild birds: vectors of *S.m*., cattle of *S.d.*, humans of *S.t.,* but also contaminated feedstuffs and water courses. Transmission by ingestion	Variable.	Abortion and death of pregnant ewes. *S.t.* and *S.d:* Profuse dysentery, tooth grinding, pyrexia, congestion of mucuous membranes, no ruminal sounds, severe metritis after abortion*). S.m.*: Affected ewes: dull, depressed, isolated from flock, have foetid red-brown vaginal discharge	Yes: triggered by climatic changes, shipping, overcrow-ding, food and/or water deprivation, inappropriate use of antibiotics	Yes	*S.t.*: ewes found dead with autolytic/ emphysematous lambs *in utero*, metritis or septic peritonitis	Isolation of all aborted sheep for 6 weeks	Yes	1, 3, 15

**Viruses**										
Bluetongue virus (BTV)	IN, L, REP, RES	Transmitted by *Culicoides* spp. (insect host) after becoming infected by feeding on viraemic animals. Transmission through semen and blood also possible	5-10 days	Rarely symptomatic	No	No	Coronitis, ulcerations of the oral mucosa, muzzle oedema.	Disease-free areas: vaccination, animal movement control, quarantine and screening, vector control, especially in aircrafts. Infected areas: vector control, vaccination	No	1, 16
Caprine arthritis-encephalitis virus (CAEV)	N, L, RES, I	Through fluids that contain macrophages from an infected animal. Most efficient transmission: from doe to kid by ingestion of colostrum or milk from infected does. Horizontal transmission possible (if long term cohousing). Venereal transmission from animals with clinical signs. Vertical transmission, transmission during parturition, iatrogenic transmission possible	Highly variable: usually lasts for months to years	Chronic disease. 4 clinical syndroms: arthritis (chronic, progressive, in goats > 6 months old, from lameness to recumbency over years), leucoencephalomyelitis, interstitial pneumonia and mastitis. Encephalitis in 2-6 month old kids, but also reported in younger and older animals. Polyarthritis in adult goats, some cases reported in 6 month old animals	No	Yes	Thickened joint capsules, often with periarticular mineralization, but with intact articular cartilage. Histopathology: chronic proliferative synovitis with infiltration of lymphocytes, macrophages and plasma cells	Programme of periodic testing (seroconversion occurs after 2-8 weeks) and culling of all seropositive animals to eradicate the virus from a herd. Kids to be separated from older animals and fed heat-treated colostrum. New additions to be quarantined and tested within 60 days of arrival	No	1, 5, 17
Caprine herpesvirus 1 (CpHV-1)	D, REP, RES	Venereal: selective tropism of the virus for the genital tract	10-60 days (after infection, resulting in abortion). Latency unkmown. Reactivation attempts difficult under both natural and experi-	Generalized and often lethal gastrointestinal infection in 1–2 week old kids. Most infections in adult goats subclinical, otherwise: vulvovaginitis, ulcerative (balano)posthitis, abortion storms. Less frequently infections of the respiratory tract	No	Yes	Autolyzed aborted fetuses or with lungs, liver, kidneys and adrenal glands with pinpoint white foci (randomly distributed coagulative necrosis). Microscopically: intranuclear inclusion bodies may be visible	Removal of seropositive animals. Strict biosecurity measures at the facility, maintenance of a closed herd, and separating kids from adults at birth, contributing to the success of	No	1, 18

			mental conditions				around the areas of necrosis. Mild or minimal placental damage	the surveillance programme for CpHV-1		
Caprine respiratory syncytial virus (CRSV)	RES	Through contaminated droplets, food and water. Most severe epidemics in autumn and winter	2-8 days	Fever, sluggishness, nasal discharge, deep breathing, respiratory distress, cough, anorexia	Yes	No	Macroscopically, a mucopurulent exudate in the lumen of the bronchus and bronchioles, irregular lobular or diffuse mildly collapsed gray- red foci in the cranioventral lobes of the lung. Pulmonary emphysema. Histopathologically, bronchitis, bronchiolitis, thickening in the alveolar septum with mononuclear cell infiltrations, lymphoid hyperplasia, hyperplasia in the epithelial cells of the bronchi and bronchioles, acidophilic inclusion bodies in the epithelium of the bronchi and bronchioles and syncytial cells	Separation of diseased animals	No	19
Contagious echtyma (“orf”) virus	IN, RES, D	Direct contact with clinically affected animals. Fomites contaminated by the clinically affected. Indirect, by contact with virus-contaminated soil or shed scabs or from “mechanical” carriers. Typically through a break in the skin (e.g at tooth eruption). Persistance in soil for years	3-14 days	Papules -> vesicules -> pustules -> scabs dropping off in 1-4 weeks. Self-limiting, resolving in 3 weeks Morbidity 100% in naïve flocks, mortality 1%. Death because of pneumonia or starvation due to impossibility to feed:	Yes, by trans-portation stress	No	Characteristic macroscopic scab-like lesions on the lips, muzzle, in the oral cavity. Crusty proliferations at mucocutaneous junctions. Lesions also on ears, face, periorbital region, scrotum, perianal	Isolation of affected stock, prevention of the disease entering the farm (quarantine, clinical examination of new additions and purchasing	Yes	1, 5

				e.g. severe oedema and necrosis in the mouth or painful udder and teats lesions, preventing suckling. Sometimes gastroenteritis			region, extremities. Histopathology: ballooning and degeneration of keratinocytes, eosinophilic intracytoplasmatic viral inclusions	new stock from ecthyma-free farms)		
Rotavirus group A-C	D	Mainly by fecal-oral route (excreted feces by approximately 30% of infected animals). Waterborne or airborne (respiratory) routes also suggested	Up to 48 hours	Acute diarrhea in animals aged 2-14 days, depression and dehydration	Yes	No	Intestinal villus atrophy	Immunization of dams. Colostrum supplements in milk during the period of risk. The management of pregnant animals at the time of parturition ensuring the minimum exposure of newborn animals to infectious agents	Yes – group A	1, 20, 21
**Parasites**										
Cryptosporidium parvum	D	Fecal-oral route: directly or on fomites including contaminated food and water. Sporulated oocyts (immediately infectious) shed in the feces of symptomatic and asymptomatic individuals (in stressful periods – e.g. around parturition). Severe disease by autoinfection	In young animals, clinical signs after 3-5 days	Kids aged 3-7 days old most commonly affected. Mild to severe watery diarrhea: yellow or pale brown, mucoid, anorexia, lethargy, and weight loss. Occasionally respiratory signs. Most clinical cases self-limiting within 1-2 weeks, possible. dehydration, debilitation and deaths	Yes	Yes	Not specific: increased fluidity of the intestinal contents, hyperemia of the intestinal mucosa, and distension of the large and/ or small intestine. Enlarged mesenteric lymph nodes. Microscopically: mild to severe villous atrophy in the intestines, spherical organisms in the brush border	The infective dose is low Difficult control (low infective dose). Sanitation (e.g., regular cleaning of pens) and manure management to reduce the level of exposure to oocysts. Steam cleaning and disinfection, or thorough cleaning with hot water, followed by	Yes	1, 22

								drying to promote desiccation. Good nutrition and management practices to avoid stress. Isolate sick animals.		
Dictyocaulus filaria, « lungworm »	RES	Adult *D. filaria* in the trachea and bronchi lay eggs with fully developed L1. L1 hatch quickly, are coughed up, swallowed and passed in feces. In 5 days, L3 migrate to grass tips and are ingested, penetrate the intestinal mucosa, moult to L4 in the mesenteric lymph nodes, then migrate via lymphatics and blood to the caudal lobes of the lungs	21-28 days	Normally not clinically significant. Clinically apparent if first challenge or high challenge: harsh coughing, dyspnea, rectal prolapse after severe coughing paroxysms. Mild to moderate pneumonia with coughing in lambs. Deaths uncommon (if secondary pasteurellosis)	No	Chronic, causes ill thrift	If large numbers of larvae are ingested: death - severe interstitial emphysema and Other lesions: parasitic pneumonia (ventral areas of the Caudal lung lobes), severe bronchiolitis and bronchitis. Adult worms 30 to 100 mm long in the trachea and bronchi. pulmonary edema	Prophylactic antihelmintic treatment according to the herd health plan	No	23
Fasciola hepatica	D, CV/HP	For animals on pastures: late spring /early summer infestation of snails by miracidia. Autumn: metacercariae challenge to sheep	Depending on the level of challenge: immediate acute disease, subacute disease after weeks, chronic disease apparent several months later	Acute form: sudden death by hemorrhage and liver damage, other animals lethargic, with pale mucuous membranes and with reduced grazing activity. Subacute form: rapid weight loss => very poor body condition score and poor fleece quality, marked anemia, severe depression, inappetence, weakness, unable to stand, fetal death/resorption	Yes	Yes	Acute form: liver enlargement, ascites/peritoneal exudate. Subacute form: liver enlargement, ascites/peritoneal exudate, visible only by ultrasound examination/ necropsy. Chronic form: low body condition score and poor fleece quality, submandibular oedema, anemia, death in advanced gestation/early lactation	Measures to reduce the metacercarial challenge in autumn, strategic drenching, fencing off snails' habitats (expensive measure)	Yes	1, 24
Neospora caninum	REP, N	There are 3 life cycles: tachyzoites, tissue cysts (both found in intermediate hosts such as sheep) and oocysts (found in	Not described. Reactivation of latent	Abortions, near-term, mummified stillborn fetuses	Yes	Yes	Autolyzed fetuses and placentas or placentitis with	Food should be stored in facilities	No	1, 25-27

		definitive hosts - dogs).The oocysts sporulate outside the host. The transmission is fecal-oral (sporulated oocysts can also contaminate food and water). Transplacental transmission of tachyzoites is also possible, for several generations	infection possible				necrosis and mineralization of cotyledonary vili, mild multifocal necrosis in the brain	preventing contamination by dogs' feces		
Toxoplasma gondii	N, REP	Infective oocysts from cat feces can be ingested with food or water, inhaled in aerosols. Transplacentally (vertical transmission 4%), potentially via semen	2-4 weeks	Abortions during the latter half of gestation. Embryonic resorbtion.(if infection at 1- 40 days of gestation). Fetal mummification, maceration and abortion (if infection at 40-120 days of gestation). Premature, stillborn or weak kids (if infection after 120 days of gestation). Clinically normal does clinically (except: immunosuppressed pregnant females: febrile, develop the neurological form)	No	No	Mummified fetuses: dark brown leathery appearance. 1-3 mm gray-white necrotic foci found on the dark-red cotyledons of the placenta. The intercotyledonary region usually normal or slightly edematous	Food storage in facilities preventing contamination by cats or vermin, maintenance of a healthy adult cat population by appropriate vaccination and neutering. Cats not to be allowed near pregnant goats. Vaccination possible	Yes	1, 5, 28

Body systems: CV/ HP: Cardiovascular and hematopoietic system, D: Digestive system, I: Immune system, IN: Integumentary system; skin, hoof and claw, N: Nervous system, L: Locomotor system, REP: Reproductive system, RES: Respiratory system, U: Urinary system

References

1. Pugh DG, Baird N. *Sheep and Goat Medicine*. 2nd ed.: Elsevier, 2012.

2. Spickler AR. Brucellosis: Brucella melitensis, http://www.cfsph.iastate.edu/DiseaseInfo/factsheets.php (2018, accessed 30th June 2019).

3. Fox J, Otto G, Pritchett-Corning K, et al. *Laboratory Animal Medicine*. 3rd ed.: Academic Press 2015.

4. Spickler AR. Zoonotic Campylobacteriosis, http://www.cfsph.iastate.edu/DiseaseInfo/factsheets.php (2013, accessed 6th June 2019).

5. Allen MK and Borkowski GL. *The Laboratory Small Ruminant*. 1st ed.: CRC Press, 1999.

6. Longbottom D and Coulter LJ. Animal chlamydioses and zoonotic implications. *J Comp Pathol* 2003; 128: 217-244. 2003/07/02. DOI: 10.1053/jcpa.2002.0629.

7. Scott PR. *Sheep Medicine*. 2nd ed.: CRC Press, 2015.

8. Uzal FA and Songer JG. Diagnosis of *Clostridium perfringens* intestinal infections in sheep and goats. *J Vet Diagn Invest* 2008; 20: 253-265. 2008/05/08. DOI: 10.1177/104063870802000301.

9. Roest HJ, van Gelderen B, Dinkla A, et al. Q fever in pregnant goats: pathogenesis and excretion of *Coxiella burnetii*. *PLoS One* 2012; 7: e48949. 2012/11/16. DOI: 10.1371/journal.pone.0048949.

10. Spickler AR. Q-Fever, http://www.cfsph.iastate.edu/DiseaseInfo/factsheets.php (2017, accessed 6th June 2019).

11. Bahr A and Wolf E. Domestic animal models for biomedical research. *Reprod Domest Anim* 2012; 47 Suppl 4: 59-71. 2012/08/01. DOI: 10.1111/j.1439-0531.2012.02056.x.

12. Spickler AR. Leptospirosis http://www.cfsph.iastate.edu/DiseaseInfo/factsheets.php (2013, accessed 6th June 2019).

13. Spickler AR. Paratuberculosis http://www.cfsph.iastate.edu/DiseaseInfo/factsheets.php (2017, accessed 6th June 2019).

14. Spickler AR. Contagious Agalactia, http://www.cfsph.iastate.edu/DiseaseInfo/factsheets.php (2018, accessed 6th June 2019).

15. Clark MA. Bovine coronavirus. *Br Vet J* 1993; 149: 51-70. 1993/01/01. DOI: 10.1016/S0007-1935(05)80210-6.

16. Saegerman C, Berkvens D and Mellor PS. Bluetongue epidemiology in the European Union. *Emerg Infect Dis* 2008; 14: 539-544. 2008/04/09. DOI: 10.3201/eid1404.071441.

17. Spickler AR. Small Ruminant Lentiviruses: Maedi-Visna & Caprine Arthritis and Encephalitis, http://www.cfsph.iastate.edu/DiseaseInfo/factsheets.php (2015, accessed 6th June 2019).

18. Pollock JM, Schofield MJ, Porter C, et al. Caprine herpesvirus 1: A successful eradication program in dairy goat herd. *Small ruminant research* 2019; 170: 8-11.

19. Bennett N, Ellis J, Bonville C, et al. Immunization strategies for the prevention of pneumovirus infections. *Expert Rev Vaccines* 2007; 6: 169-182. 2007/04/06. DOI: 10.1586/14760584.6.2.169.

20. Rotavirus infections in livestock and poultry, https://www.cabi.org/isc/datasheet/66267 (accessed 6th June 2019).

21. Papp H, Malik YS, Farkas SL, et al. Rotavirus strains in neglected animal species including lambs, goats and camelids. *Virus disease* 2014; 25: 215-222. 2015/02/13. DOI: 10.1007/s13337-014-0203-2.

22. Spickler AR. Cryptosporidiosis, http://www.cfsph.iastate.edu/DiseaseInfo/factsheets.php (2018, accessed 6th June 2019).

23. Ballweber LR. Overview of Lungworm Infection https://www.msdvetmanual.com/respiratory-system/lungworm-infection/overview-of-lungworm-infection (2014, accessed 6th June 2019 2019).

24. Reddington JJ, Leid RW and Wescott RB. The susceptibility of the goat to *Fasciola hepatica* infections. *Vet Parasitol* 1986; 19: 145-150. 1986/01/01. DOI: 10.1016/0304-4017(86)90042-7.

25. Dubey JP. Review of *Neospora caninum* and neosporosis in animals. *Korean J Parasitol* 2003; 41: 1-16. 2003/04/02. DOI: 10.3347/kjp.2003.41.1.1.

26. Dubey JP and Lindsay DS. A review of *Neospora caninum* and neosporosis. *Vet Parasitol* 1996; 67: 1-59. 1996/12/02. DOI: 10.1016/s0304-4017(96)01035-7.

27. Dubey JP and Lindsay DS. Neosporosis, toxoplasmosis, and sarcocystosis in ruminants. *Vet Clin North Am Food Anim Pract* 2006; 22: 645-671. 2006/10/31. DOI: 10.1016/j.cvfa.2006.08.001.

28. Spickler AR. Toxoplasmosis, http://www.cfsph.iastate.edu/DiseaseInfo/factsheets.php (2017, accessed 6th June 2019).
