# Supplementary material for: Federation of European Laboratory Animal Science Associations recommendations of best practices for the health management of ruminants and pigs used for scientific and educational purposes
Source: Lab Anim. 2020 Aug 9;55(2):117–28. doi: 10.1177/0023677220944461 (PMC8044623; doi:10.1177/0023677220944461)

Appendix 9. Examples of supply chains

9.1. Example of supply chain for pigs

9.1.1. Breeding pyramid

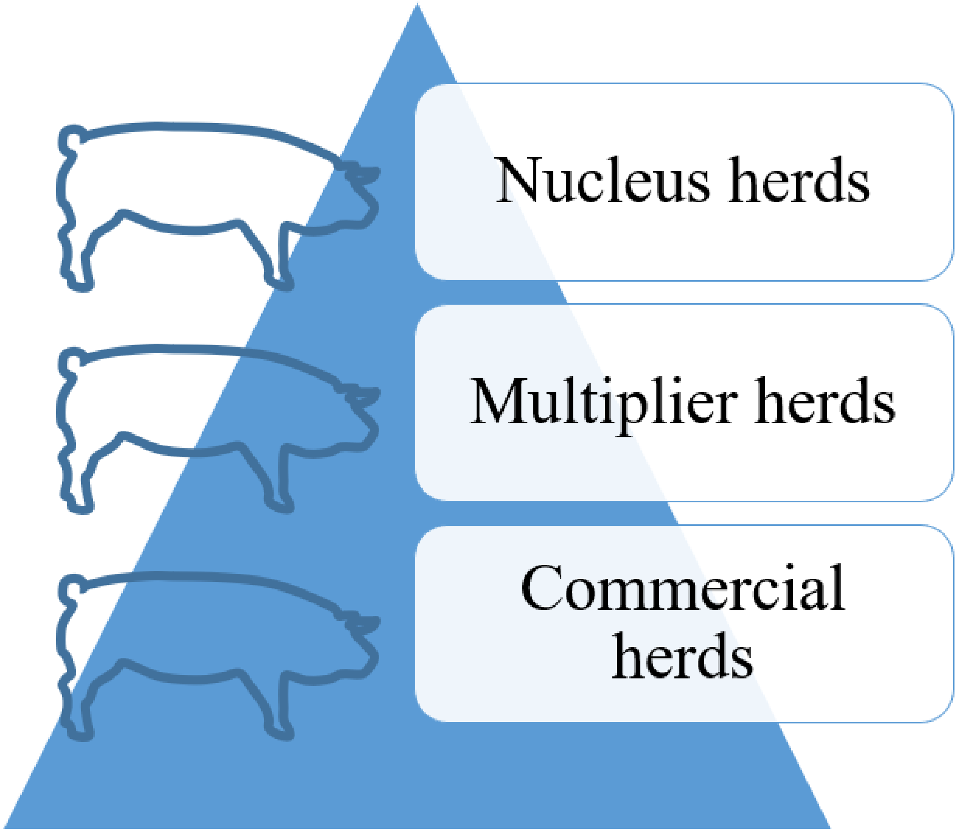

Systematic breeding pyramid systems in pig production safeguard unidirectional flow to ensure high health and performance.

At the top of the pyramid, the nucleus herds produce pure genetic lines where constant genetic progress is pursued.

The next level consists of multiplier herds producing maternal hybrid lines. These make up the pool to be used in commercial herds, producing grower-finisher pigs.

Depending on the nature of the investigation in question, animals should be purchased from the top, middle or the bottom of the breeding pyramid.

9.1.2. Sow pool production system

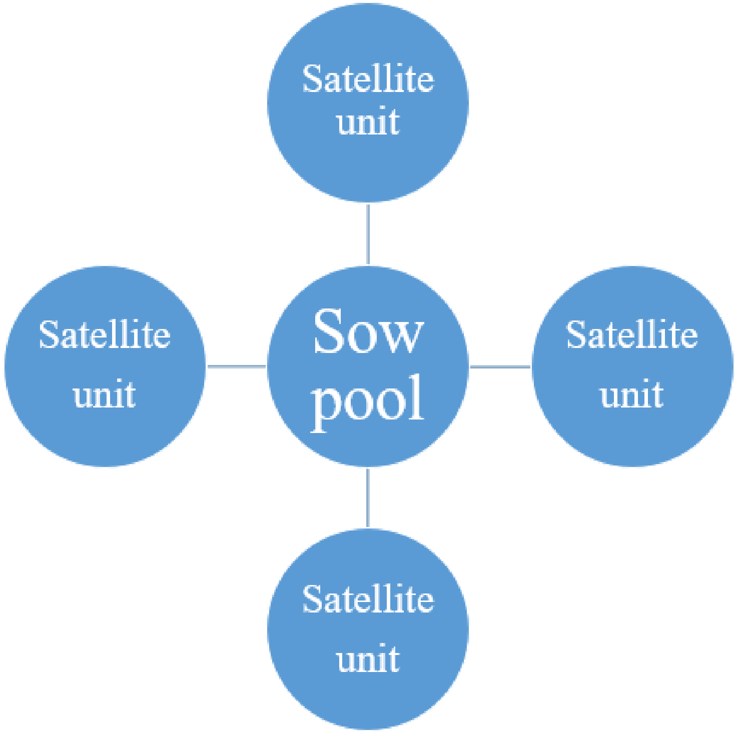

The sow pool production system consists of one large central gestation herd, supplying the cooperating producers (satellite units) with pregnant sows in a leasing system.

9.2. Example of supply chain for cattle

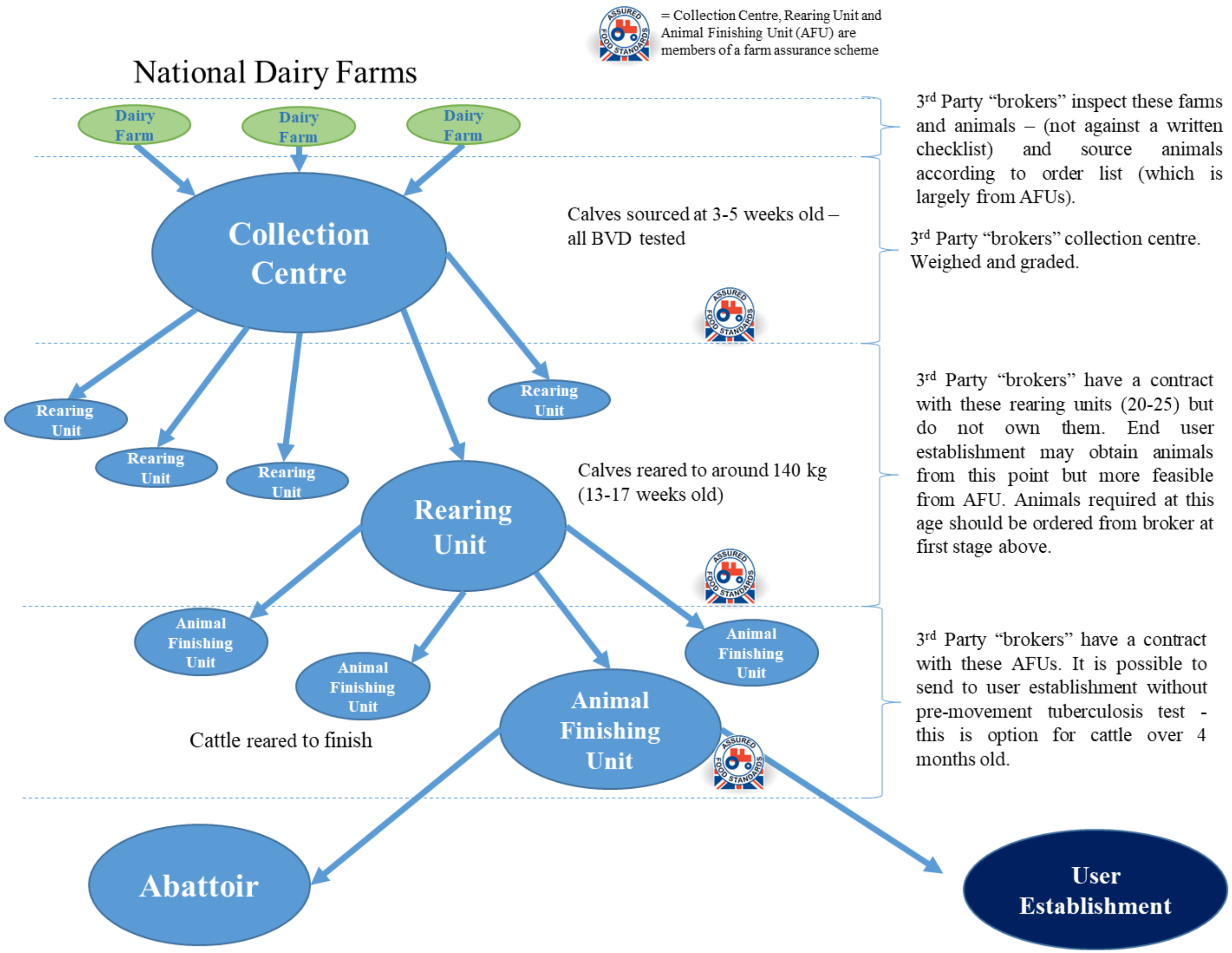

9.3. Example of supply chain for sheep

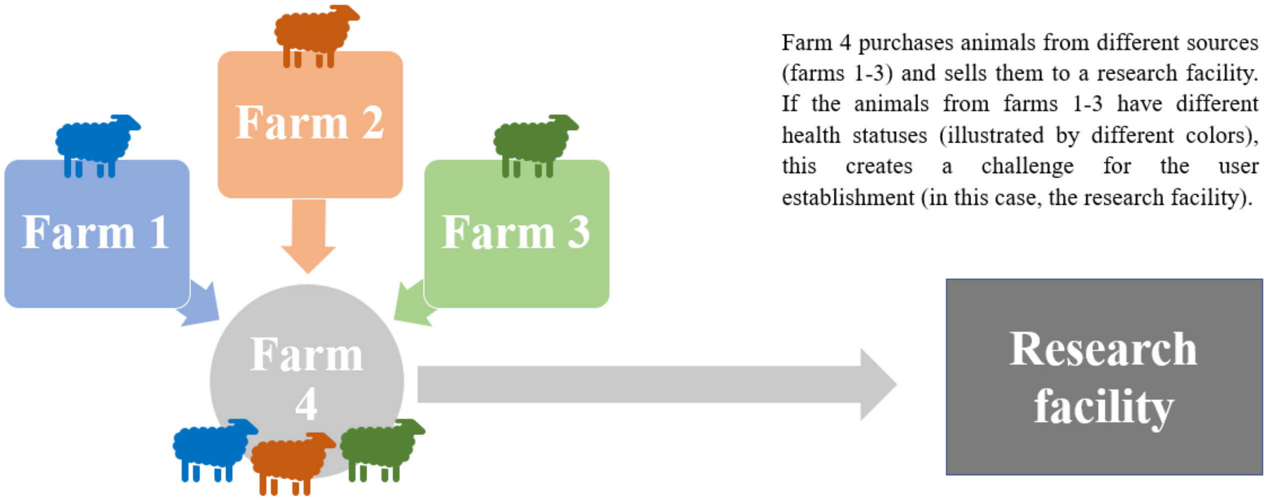

Supplement: sj-pdf-9-lan-10.1177_0023677220944461 - Supplemental material for Federation of European Laboratory Animal Science Associations recommendations of best practices for the health management of ruminants and pigs used for scientific and educational purposes [file sj-pdf-9-lan-10.1177_0023677220944461.pdf]
