# Supplementary material for: Federation of European Laboratory Animal Science Associations recommendations of best practices for the health management of ruminants and pigs used for scientific and educational purposes
Source: Lab Anim. 2020 Aug 9;55(2):117–28. doi: 10.1177/0023677220944461 (PMC8044623; doi:10.1177/0023677220944461)
Supplement: sj-pdf-10-lan-10.1177_0023677220944461 - Supplemental material for Federation of European Laboratory Animal Science Associations recommendations of best practices for the health management of ruminants and pigs used for scientific and educational purposes [file sj-pdf-10-lan-10.1177_0023677220944461.pdf]

Appendix 10. Management in ABSL-3 conditions

Maintaining ruminants or pigs in high biocontainment level may lead to serious challenges, mainly related to effluent or solid waste collection and treatment for decontamination, and to occupational safety when housing and handling the animals.

When effluents and solid waste have to be decontaminated, they need to be collected and stored before the decontamination process. Due to the nature of these liquid and solid waste, thermal decontamination (autoclave) is the most reliable solution, especially in ABSL-3 when the environmental risk is elevated. This leads to avoiding or to minimizing the use of roughage (for feeding) and substrate (for litter or bedding), due to the volume of solid waste generated and to the risk of obstruction/clogging of the liquid waste system (ducts, pumps, tanks).

In addition to waste management issues, introduction, minimal storage and handling of large quantities of bulky roughage or substrate may also be an issue in high containment facilities. As priority is given as a rule to public and personnel health protection, acceptable solutions have to be identified and approved during the ethical review.

Replacement of roughage for ruminants feeding can be managed by special diets using chopped hay or straw or pellets containing the required amount of cellulosic fibers. Of course, the suitability of this type of replacement must be carefully assessed and tested.

Adequate room temperature should also be considered. At lower temperatures (than thermoneutral), bedding is important to help the animals to keep warm enough. Floor heating can be considered to provide more comfort. Sheep should be shorn before they are housed indoors to avoid the risk of overheating. With ruminants, straw litter in ABSL-3 is commonly replaced by rubber mats with an adapted cleaning frequency. With pigs, with a suitable pen design, substrate can only be used as bedding in the pen resting area (i.e. in small amounts, low replacement frequency) and not as litter, orienting animal defecation and urination in a dedicated pen area. Of course, using additional enrichment is also a classic approach with pigs.

Regarding environmental enrichment, several non-food/ non-bedding devices can be introduced to provide behavioral substrates for the animals (slow feeding devices, and for pigs also objects like balls, hanging chains etc. to manipulate).

If the risk assessment allows avoiding thermal / autoclave decontamination (lower risk and biocontainment level, e.g. ABSL-2), other solutions are sometimes applicable with manure. Chemical methods can be investigated depending on the biological agent. As an example, manure can be collected by gravity in a tank and slaked lime ($Ca(OH)_2$, lime hydrate, calcium hydroxide) can be used in a 40% solution with 40-60 L/m^3, a suitable stirring system and an exposure time of 4 to 7 days, even with temperatures between 0 and -10°C.[1] An advantage is that, depending again on the outcome of the risk analysis, the possibility to use agricultural spreading.

Last but not least, it is worth mentioning that the housing of animals of high health status (e.g. pig colonies) under strict bio-exclusion may present similar issues and solutions. With farm animals, personnel safety is another critical issue in biocontainment area as primary biocontainment is often implemented at room level furnished with open pens for the animals. Then, people have to do their work in the primary biocontainment, and animal approach and handling may present a significantly increased risk.

References

1. Haas B, Ahl R, Bohm R, et al. Inactivation of viruses in liquid manure. *Rev Sci Tech* 1995; 14: 435-445. 1995/06/01. DOI: 10.20506/rst.14.2.844.
