# Supplementary material for: Federation of European Laboratory Animal Science Associations recommendations of best practices for the health management of ruminants and pigs used for scientific and educational purposes
Source: Lab Anim. 2020 Aug 9;55(2):117–28. doi: 10.1177/0023677220944461 (PMC8044623; doi:10.1177/0023677220944461)
Supplement: sj-pdf-11-lan-10.1177_0023677220944461 - Supplemental material for Federation of European Laboratory Animal Science Associations recommendations of best practices for the health management of ruminants and pigs used for scientific and educational purposes [file sj-pdf-11-lan-10.1177_0023677220944461.pdf]

Appendix 11. Glossary

ANTIBODY-FREE	Some research or testing applications may require the provision and use of "antibody-free" animals in addition to other health status requirements, i.e. using animals free from predefined antibodies. When relevant, such a requirement may have to be added to the health status specifications.
COMMENSAL MICROBIOTA	A commensal or resident microbiota is defined as the normal, stable microorganism populations, mainly bacteria, found associated with a healthy host organism in different parts of the body (intestine, skin, mouth, vagina). Acquisition starts at birth and, depending on the environmental conditions, becomes enriched with various microorganisms originating from the environment, food, other animal species. A synergistic commensal microbiota can be defined as one having a demonstrable benefit to the host health, by improving digestion, metabolism, immunity or by causing an antibiosis phenomenon, i.e. a biological interaction between the host commensal microbiota and undesirable microorganism(s), detrimental to the latter. As an example, intestinal or skin bacteria ecologically adapted to the host organism may prevent stable intestinal colonization by other less adapted microorganisms, including pathogens and opportunists and contribute significantly to the stability of a healthy condition. Such a microbiota "barrier" effect is one of the reasons why one should pay attention to the microbiota.
EXCLUSION LIST and SCREENING LIST	A health management and monitoring programme is based on an exclusion list, i.e. a negative definition detailing all organisms to be excluded from a defined animal group. The exclusion list should be predetermined (according to research and animal-related requirements) to include all undesirable parasites (uni- or multicellular), bacteria and viruses and, for very specific applications, from ecotropic retroviruses or unconventional transmissible agents (prions). The list should match the specific expectations and research activities, with a clear reference to the methods used to monitor the colony. An exclusion list should be adapted to the type of research activities envisaged and include at least primary species pathogens, zoonotic agents and major interfering agents.<br>When defining a health standard and related monitoring techniques, it is critical to keep in mind the difference between the "screening list" and the "exclusion list". The first can be purely informative, i.e. aiming at monitoring components of the resident microflora in order to assess the global efficiency of the bioexclusion system. The second would, in the case of a positive result, lead to a decision to invalidate experimental results obtained using such animals and for the breeder, to consider recycling the "contaminated" colony. In an agricultural setting, the breeder/supplier may decide otherwise unless he wants to continue supplying this special customer. Only in case of a notifiable disease, the recycling would be mandatory for the supplier. In both cases, in addition to the research impact, ethical, practical and economic consequences are generally severe. Some screening lists may include a large number of agents, as they are

	established by testing laboratories for large-scale "one fits all" automated methods, such as qPCR (Real-Time / Taq-Man) with high-density PCR arrays, or as multiplex fluorometric immune-assays (MFIA).[1]
GNOTOBIOTIC or GNOTOXENIC	Refers to animals living in the absence of any known and detectable microorganism (axenic or germfree animals) or associated with a well-defined microflora, mainly in the digestive tract (that harbours the highest number of associated microorganisms), on the skin and in various body cavities.
HEALTHY CARRIERS	Animal pathogenic, parasitic or zoonotic agents can be harboured, carried, shed and transmitted by "healthy carrier" animals, i.e. by individuals not displaying any clinical sign or lesion, hence the importance of health monitoring.
HEALTH MONITORING	Health monitoring is a component of the health management programme, aiming at demonstrating the absence of disease, infection or non-suitable biological agents, and detecting them as early as possible.
HEALTH PROGRAMME	A health programme can be defined as the policies, procedures, standards, roles and responsibilities, work organization, facilities, equipment, all resources and practices defined and implemented by a site or an institution to achieve an effective and comprehensive health management of animals. It is designed to allow maintaining a predefined animal health status. It should include all activities conducted by and at the site / institution that may have a direct impact on the health and well-being of animals. It primarily oversighted by the designated veterinarian, with the contribution of the animal welfare officer, the animal welfare body and personnel in charge of study design and conduct, and animal care. Health monitoring is a component of the health management programme.
HEALTH STATUS and STANDARD	For laboratory animals, the health definition of an animal breeding colony of experimental group, is generally based on the "exclusion list".<br>The "health standard" rather refers to the selected definition and objective of the health management programme, and "health status" is generally related to the actual health situation of the colony or group (current health monitoring results).<br>Increasingly, breeding colonies or animal groups are also defined with a "positive list" including a minimum microbiota definition. In gnotobiology, gnotoxenic animals are mainly defined by their microbiota (bacteria), and should be free from any other known and detectable biological agents (virus, fungi, yeast and parasites) and live in a mold-free environment.
INTERFERING AGENTS	Agents which may or may not have any pathogenic or opportunistic capacity but which have the potential to interfere with the study and the scientific aims.[2]
MICROBIOTA and MICROBIOME	The microbiota is defined as all microscopic organisms harbored by a living organism, with focus on bacteria. It consists of a wide variety of bacteria, viruses, fungi, and other single-celled animals that live in or on the body. Only "germ-free" or "axenic" animal are deprived from microbiota. The microbiota can be more or less complex, depending on the type of study and animals monoxenic, oligoxenic, polyxenic. Larger microbiota are used for studies on bacteria

	cooperation, synergies, or co-implantation phenomena. Some “standard” microbiota are defined with 10 or 12 selected bacteria. More complex microbiota from rodent or human donors are used for fecal microbiota transplantation).<br>The microbiome is either synonymous to microbiota or is the name given to the collective genomes of all microorganisms making the microbiota.
OPPORTUNISTIC AGENTS	Some components of the microbiota, not belonging to the exclusion list can be potential opportunistic agents, able to cause either pathological condition or experimental interference under special conditions such as immunodeficiency, stress, poor environmental conditions or fragilization (such as poor general conditions or ageing). It is the responsibility of the breeder and the investigator to decide on their relevance and to add them to the exclusion list or the screening list when relevant. Agents interfering with a specific research application or playing a synergistic role when associated with other viruses or other bacteria should be addressed in a similar way. The concepts of both “interfering” and “opportunistic” agents are relative, varying with the type of research and animal environment.
PATHOGENIC AGENTS and PATHOGENICITY	A pathogen is an agent (virus, bacterium, fungus, protozoan or metazoan endo- and ectoparasites) capable to induce a disease, even with a low infectious load, in a defined species, in immuno-competent animal, whatever the experimental conditions, sometimes with a trend to persist in the host.<br>Minor pathogens may cause a disease with a low severity (low or moderate morbidity and virulence, and recovery without sequela. Major pathogens have the potential to combine a high morbidity, mortality and / or sequelae.<br>As an example, the most common coagulase positive staphylococcus isolated from the cow's mammary gland are *Staphylococcus aureus.* They are considered as major mastitis pathogens because many strains are contagious, harbored in the cow's mammary gland, and spread from cow-to-cow at the time of milking. In contrast, coagulase negative staphylococci have historically been classified as minor mastitis pathogens and are rarely, if ever, further differentiated when diagnosing an intra-mammary infection.[3, 4]<br>Pathogenicity refers to the capacity of an organism to cause disease and overt damage to a host. It can be strain or bio-type dependent. It is related to its transmissibility from one host or reservoir to a new host, to its survival in the new host, its infectivity and its ability to resist host defenses. The latest is also depending on the host resistance. Influenza virulence and pathogenicity can be measured using parameters of morbidity and mortality within animal models.<br>Infectivity is the ability to infect the host, that can be infected but not sick. Some organisms are harmless and can live without any risk (commensal or symbiotic organism). The virulence is the degree of disease severity. Some very virulent influenza viruses generate severe lesions and clinical signs and can lead to death. Low virulence strains cause a disease with a lower severity: they are infectious, pathogenic and provoke a defense reaction from the host. In this context, the morbidity refers to the percentage of sick individual within a group or a population and mortality is the number of individuals dying within a group or a population. Another feature of is the potential to cause sequelae, which are defined as a chronic lesion due to and following the acute stage of the infection.

PREBIOTICS and PROBIOTICS	At the time of this early intestinal microbiota implantation, or at later stages, it might be useful to consider using probiotic microorganisms, defined as "live microorganisms, which if administered in adequate amounts and with adequate feeding, confer a health benefit on the host".<br>Probiotics should not be confused with prebiotics, which are defined as "food ingredients (e.g. oligo- and polysaccharides) that stimulate the growth and / or activity of bacteria in the digestive system and are beneficial to the health of the body", i.e. that are used as a substrate by microorganisms already in the host's intestine. Therapeutic or prophylactic use of probiotics and/or prebiotics may help to stimulate the production of IgA antibodies and macrophage activity phagocytosis, to decrease the number of inflammatory mediators and to decrease intestinal pH and thus the growth and activity of undesirable intestinal bacteria. However, it appears that the composition of the microbiota of most individuals is quite stable especially in the large intestine. It is easier to modify the microflora of the small intestine, which harbours a much lower number of bacteria. Influencing an established small intestine microflora may require 10^4–10^5 bacteria per dose and repeated doses, with no guarantee of lasting effect. A much higher dosage is required for the large intestine.
REDERIVATION	When an animal colony is declared "contaminated", i.e. positive for unwanted agents not compatible with the expected health standard or not complying with the exclusion list), several techniques can be used to eliminate these non-acceptable agents or, to use a common wording, to "rederive" the animals. The selection of an appropriate technique depends on many factors such as the animal species, the number and the nature of the agent(s) to eliminate. The term "re-derivation" is generally used for the most efficient and sophisticated techniques involving "aseptic hysterectomy or hysterotomy" or "embryo transfer", especially for pig colonies. These are based on a very simple (but not absolute) principle: the sterility of the reproductive tract during pregnancy until the onset of parturition and opening of cervix. Failure may occur if there is "false" vertical transmission, resulting from contamination during the process or a "real" vertical transmission when the pathogen infects the offspring *in utero*, before or after embryo implantation. The aseptic hysterotomy / caesarean section has been widely used of obtain contaminant-free piglets or calves. Before cervix opening at the end of pregnancy, fetuses are removed from the uterus by caesarean section and transferred aseptically into a sterile housing entity, generally an isolator or a clean room. When antibody-free status is also expected since delivery, the newborns can be hand-fed on artificial milk until weaning age. They are kept isolated until health monitoring results are available. These techniques are used to obtain colostrum-deprived and antibody-free ruminants, with no or very limited contact with environmental microorganisms.<br>Important notice: these procedures should be included and described in the research project, and conducted under the responsibility of competent personnel. Other rederivation techniques, less efficient and / or limited to certain agents, may also be used.
SCREENING LIST	See EXCLUSION LIST

TRANSIT MICROFLORA	Unlike stable commensal or resident microbiota, some environmental or human-borne microorganisms can be detected over a short period of time before disappearing or reaching a non-detectable level.
ZOONOTIC AGENTS	Zoonotic agents are agents causing zoonotic diseases or zoonoses, which are infections transmitted from vertebrate animals to humans. Zoonoses may be bacterial, viral, or parasitic, or involve unconventional agents. The absence of these animal-borne human pathogens should always be guaranteed by the strict implementation of biosecurity and health monitoring programmes. Any suspicion should be immediately reported and addressed. Biocontainment facilities and practices may be used if available at a suitable level, based on a preliminary risk assessment and mitigation. Practices may also include vaccinations of staff. All legal requirements regarding reportable diseases and biosafety shall be strictly implemented.

References

1. Reuter JD and Dysko RC. Quality Assurance / Surveillance Monitoring Programs for Rodent Colonies. In: Reuter JD and Suckow MA, (eds.). *Laboratory Animal Medicine and Management*. Ithaca, New York, USA: International Veterinary Information Service (www.ivis.org), 2003.

2. Balansard I, Cleverley L, Cutler KL, et al. Revised recommendations for health monitoring of non-human primate colonies (2018): FELASA Working Group Report. *Lab Anim* 2019; 53: 429-446. 2019/05/10. DOI: 10.1177/0023677219844541.

3. De Vliegher S, Fox LK, Piepers S, et al. Invited review: Mastitis in dairy heifers: nature of the disease, potential impact, prevention, and control. *J Dairy Sci* 2012; 95: 1025-1040. 2012/03/01. DOI: 10.3168/jds.2010-4074.

4. Pyorala S and Taponen S. Coagulase-negative staphylococci-emerging mastitis pathogens. *Vet Microbiol* 2009; 134: 3-8. 2008/10/14. DOI: 10.1016/j.vetmic.2008.09.015.
