# Supplementary material for: Federation of European Laboratory Animal Science Associations recommendations of best practices for the health management of ruminants and pigs used for scientific and educational purposes
Source: Lab Anim. 2020 Aug 9;55(2):117–28. doi: 10.1177/0023677220944461 (PMC8044623; doi:10.1177/0023677220944461)
Supplement: sj-pdf-12-lan-10.1177_0023677220944461 - Supplemental material for Federation of European Laboratory Animal Science Associations recommendations of best practices for the health management of ruminants and pigs used for scientific and educational purposes [file sj-pdf-12-lan-10.1177_0023677220944461.pdf]

Supplementary Material – Table of Contents

Appendix 1. Legal requirements and recommendations

Appendix 2. Roles and responsibilities in the user establishment

Appendix 3. Quality and Technical Agreement

Appendix 4. Herd health plan and farm biosecurity policy

Appendix 5. Examples of agents for pigs

Appendix 6. Examples of agents for cattle

Appendix 7. Examples of agents for sheep

Appendix 8. Examples of agents for goats

Appendix 9. Examples of chain supplies

Appendix 10. Management in ABSL-3 conditions

Appendix 11. Glossary
